# Incorporating variant frequencies data into short-term forecasting for COVID-19 cases and deaths in the USA: a deep learning approach

**DOI:** 10.1016/j.ebiom.2023.104482

**Published:** 2023-02-21

**Authors:** Hongru Du, Ensheng Dong, Hamada S. Badr, Mary E. Petrone, Nathan D. Grubaugh, Lauren M. Gardner

**Affiliations:** a Center for Systems Science and Engineering, Johns Hopkins University, Baltimore, MD, 21218, USA; b Department of Civil and Systems Engineering, Johns Hopkins University, Baltimore, MD, 21218, USA; c Department of Earth and Planetary Sciences, Johns Hopkins University, Baltimore, MD, 21218, USA; d Department of Epidemiology of Microbial Diseases, Yale School of Public Health, New Haven, CT, 06510, USA; e Department of Ecology and Evolutionary Biology, Yale University, New Haven, CT, 06510, USA; f Department of Epidemiology, Johns Hopkins Bloomberg School of Public Health, Baltimore, MD, 21205, USA

**Keywords:** Deep learning, LSTM, COVID-19, SARS-CoV-2, Coronavirus, Pandemic, Forecast, Prediction, US, State-level, Variant frequencies data

## Abstract

**Background:**

Since the US reported its first COVID-19 case on January 21, 2020, the science community has been applying various techniques to forecast incident cases and deaths. To date, providing an accurate and robust forecast at a high spatial resolution has proved challenging, even in the short term.

**Method:**

Here we present a novel multi-stage deep learning model to forecast the number of COVID-19 cases and deaths for each US state at a weekly level for a forecast horizon of 1–4 weeks. The model is heavily data driven, and relies on epidemiological, mobility, survey, climate, demographic, and SARS-CoV-2 variant frequencies data. We implement a rigorous and robust evaluation of our model—specifically we report on weekly performance over a one-year period based on multiple error metrics, and explicitly assess how our model performance varies over space, chronological time, and different outbreak phases.

**Findings:**

The proposed model is shown to consistently outperform the CDC ensemble model for all evaluation metrics in multiple spatiotemporal settings, especially for the longer-term (3 and 4 weeks ahead) forecast horizon. Our case study also highlights the potential value of variant frequencies data for use in short-term forecasting to identify forthcoming surges driven by new variants.

**Interpretation:**

Based on our findings, the proposed forecasting framework improves upon the available state-of-the-art forecasting tools currently used to support public health decision making with respect to COVID-19 risk.

**Funding:**

This work was funded the 10.13039/100000001NSF Rapid Response Research (RAPID) grant Award ID 2108526 and the 10.13039/100000030CDC Contract #75D30120C09570.

**Research in context**

**Evidence before this study**

A systematic review of the COVID-19 forecasting and the EPIFORGE 2020 guidelines reveal the lack of consistency, reproducibility, comparability, and quality in the current COVID-19 forecasting literature. To provide an updated survey of the literature, we carried out our literature search on Google Scholar, PubMed, and *medRxi*, using the terms “Covid-19,” “SARS-CoV-2,” “coronavirus,” “short-term,” “forecasting,” and “variant frequencies data.” Although the literature includes a significant number of papers, it remains lacking with respect to rigorous model evaluation, interpretability and translation. Furthermore, while SARS-CoV-2 genomic surveillance is emerging as a vital necessity to fight COVID-19 (i.e., wastewater sampling and airport screening), there is a clear gap between the development of COVID-19 forecasting tools and genomic epidemiology. Coupling these efforts and the respective research teams is critical for maximizing the value of variant frequencies data within modeling tools to aid pandemic preparedness efforts.

**Added value of this study**

We propose a multi-stage deep learning model to forecast COVID-19 cases and deaths with a horizon window of four weeks. The data driven model relies on a comprehensive set of input features, including epidemiological, mobility, behavioral survey, climate, and demographic. We present a robust evaluation framework to systematically assess the model performance over a one-year time span, and using multiple error metrics. This rigorous evaluation framework reveals how the predictive accuracy varies over chronological time, space, and outbreak phase. Further, a comparative analysis against the CDC ensemble, the best performing model in the COVID-19 ForecastHub, shows the model to consistently outperform the CDC ensemble for all evaluation metrics in multiple spatiotemporal settings, especially for the longer forecasting windows. We also conduct a feature analysis, and show that the role of explanatory features changes over time. Specifically, we note a changing role of climate variables on model performance in the latter half of the study period. Lastly, we present a case study that reveals how incorporating variant frequencies data may improve forecasting accuracy compared to a model without variant frequencies data.

**Implications of all the available evidence**

Results from the robust evaluation analysis highlight extreme model performance variability over time and space, and suggest that forecasting models should be accompanied with specifications on the conditions under which they perform best (and worst), in order to maximize their value and utility in aiding public health decision making. The feature analysis reveals the complex and changing role of factors contributing to COVID-19 transmission over time, and suggests a possible seasonality effect of climate on COVID-19 spread, but only after August 2021. Finally, the case study highlights the added value of using variant frequencies data in short-term epidemiological forecasting models, especially during the early stage of new variant introductions.

## Introduction

By January 31st, 2022, over 55 million cases and 850 thousand deaths have been attributed to SARS-CoV-2 virus in the US.^1^^,^^2^ Since the start of the pandemic, and in response to the need to allocate (often limited) resources and help guide policy making, the scientific community has sought to predict the spread of COVID-19.^3^, ^4^, ^5^, ^6^ Various prospective modeling efforts exist to forecast short-term (i.e., weeks) epidemiological outcomes (cases, deaths, and hospitalizations), as well as conduct longer term (i.e., months) scenario analysis.

The approaches applied by researchers to generate short-term COVID-19 forecasts can broadly be categorized into three approaches: mechanistic, statistical, and hybrid modeling. Multiple mechanistic modeling approaches have been applied to COVID-19 forecasting, which explicitly represent transmission dynamics in a population through the use of compartment models such as Susceptible-Infected-Recovered (SIR) and extensions.^7^, ^8^, ^9^, ^10^ An alternative to the mechanistic approach is statistical modeling, which estimates the mathematical representation of observed behavior directly from available data. These methods typically rely upon machine learning techniques for forecasting, which most commonly include time series,^11^^,^^12^ decision tree,^13^ and deep learning approaches.^14^ The long short-term memory network (LSTM) occupies an important position among all deep learning methods due to its advantages in processing time series data. Researchers have applied various frameworks of LSTM to forecast COVID-19 epidemiological outcomes for the U.S. at different spatial resolutions.^15^, ^16^, ^17^, ^18^ The third modeling approach merges mechanistic and statistical methodologies, here referred to as hybrid models, which take advantage of the strengths of each method to improve model performance.^19^ For example, the DeepGLEAM model combines a stochastic compartmental simulation model with deep learning for COVID-19 forecasting.^20^ All approaches utilized to date have their own strengths and weaknesses. Mechanistic models are good at providing epidemiological explanations for observed behavior, and are capable of explicitly analyzing different policies such as mask mandate and other social distancing measures through model parameterization; however, these modeling frameworks are limited in their ability to capture rapid changes in disease spreading behavior or consider potential risk factors other than those represented within the compartmental framework.^15^ In contrast, statistical models, while flexible enough to include any potential variable of interest, heavily rely on the quality and availability of the required input data, and critically, the outputs are not constrained to adhere to feasible viral dynamics. One approach to mitigate the method-specific weaknesses is to use ensemble models, such as the CDC COVID-19 Forecast Hub model, which compile multiple models of various approaches within a single prediction framework.^21^ This approach has consistently proven to be the most robust, and best performing approach for short term COVID-19 forecasting efforts, and thus why we evaluate our model against it.

Whatever the method, a recognized shortcoming in the existing COVID-19 modeling literature is the lack of rigorous and robust evaluation, which is critical to assess and compare model performance.^22^ On October 19th 2021, the CDC COVID-19 Forecast Hub published the EPIFORGE guidelines to attempt to improve the quality of models, highlighting the importance of consistency, interpretability, reproducibility, and comparability of models.^23^ However, most model evaluation presented in the published literature remains incomprehensive.^22^ Many models are evaluated for a single forecasting period, according to a single error metric, and sometimes not evaluated retrospectively at all.^22^

Furthermore, many of the existing studies do not account for critical factors or novel data sets, such as human behavior, which are available through mobility data and/or real-time survey data, or variant frequencies data,^24^ which is becoming increasingly available and of higher quality. Additionally, there is a substantial gap between model development and model implementation for real-time forecasting, and many of the models mentioned above lack guidance on when and where each model would be most suitable, let alone information on if, when and where they were applied.

In this study we address these existing gaps in the literature and provide a more reliable source of COVID-19 forecasts for policymakers and the public. We proposed a deep learning model to forecast the US COVID-19 cases and deaths for all 50 states, for 1- to 4-week forecasting windows. The model incorporates epidemiological (cases, deaths, hospitalizations, vaccinations), mobility, survey, climate, demographic, and variant frequencies data. Our work complements a recent study that incorporates viral variant data among other novel data sets into an LSTM framework for forecasting COVID-19, applied to three cities in Japan during the Delta wave.^25^ We assess the model performance based on multiple error metrics, as well as for varying time periods, regions, and as a function of different outbreak phases, namely periods of intense growth, decline and stability. Lastly, we implement a retrospective case study incorporating variant frequencies data for the Delta and Omicron waves to demonstrate the value of incorporating new variant introductions within forecasting tools. A critical contribution of this case study is bridging the typically disparate efforts and groups that generate raw genomic sequence data (from GISAID) and develop real-time forecasting tools. The incorporation of this GISAID data, even in aggregate population level form, provides critical evidence for an added value of long-term sequencing efforts. We conduct feature importance analysis to investigate the role of each feature in predicting COVID-19 cases, which further highlights the value of the variant frequencies data within the proposed modeling efforts.

## Methods

COVID-19 transmission patterns have proven complex over time. Thus, forecasting even near-term disease dynamics requires a robust predictive modeling framework and carefully selected input data streams. Critically, the framework must account for nonlinear interactions between the considered factors affecting the transmission dynamics and uncertainty in their time-dependent impact on observed transmission dynamics. We therefore propose a multi-stage deep learning framework, which, at each stage, forecasts a chosen target variable for the seven days ahead (e.g., one-week ahead forecast). The multi-stage model builds off the initial first stage prediction to forecast an additional week out and continues to implement this iterative approach one stage at a time, to predict further into the future. In this paper, we will focus on 4-stage forecasting, which generates 4-week ahead predictions, consistent with the CDC COVID-19 Forecast Hub.^26^^,^^27^ However, the framework can be applied to shorter- and longer-term horizons.

### Multi-stage LSTM network architecture

**Fig. 1:**
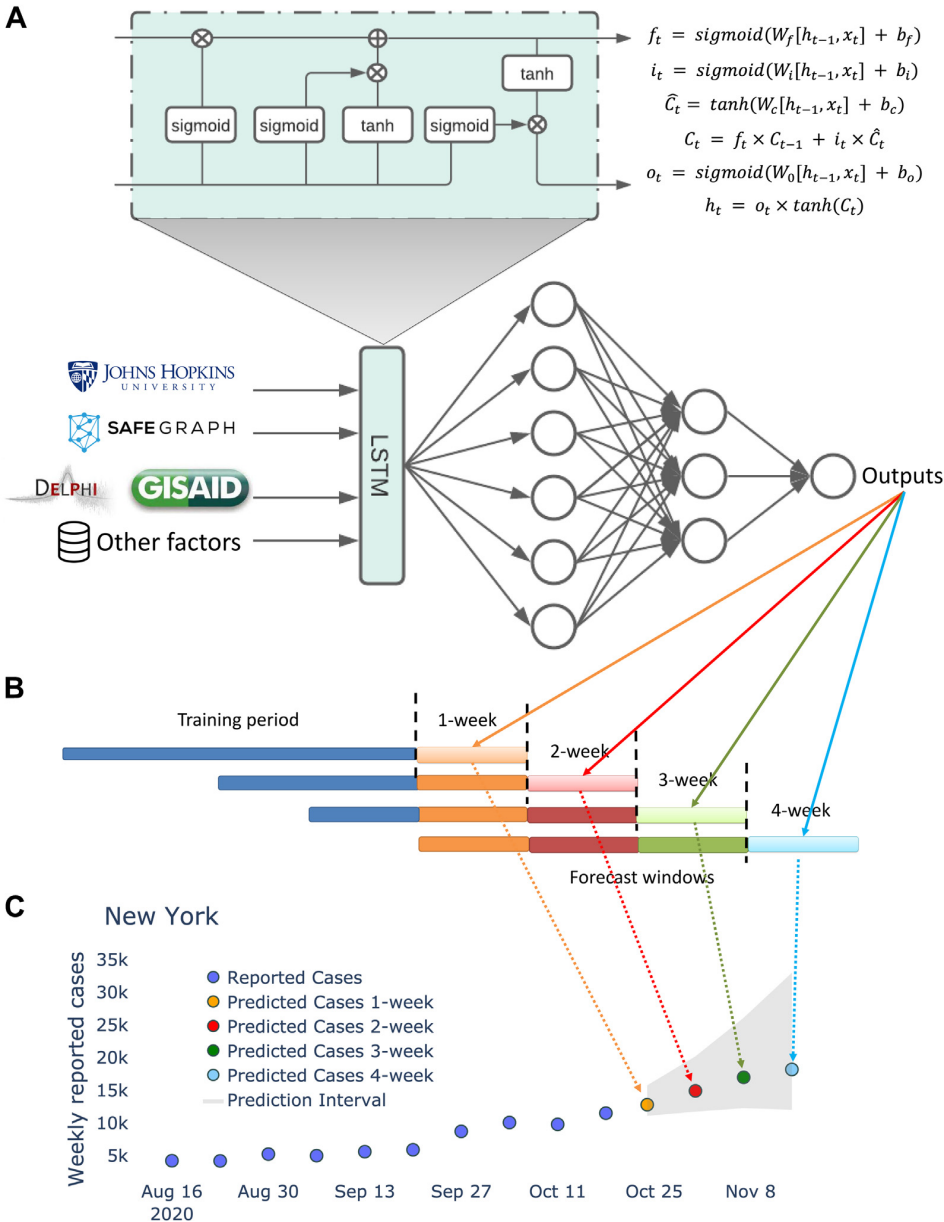
A) Network architecture of the multi-stage LSTM model. B) Prediction structure of the multi-stage LSTM model. At the initial stage, the model uses the most recent data as input, then at the later stage, the model adapts previous prediction as input to make further predictions. The transparent colors represent the model's output, and solid colors represents the model's inputs. C) An example forecasting of the multi-stage LSTM model.

The multi-stage framework consists of two neural network branches, connected in parallel, as illustrated in Fig. 1. The main branch (main model) predicts the target epidemiological variables of interest, while the secondary branch (feature model) predicts the features to populate the data streams used as input in the main model. The target variable for the main model is either weekly incident cases or weekly mortality rate; for the features model, target variables are all other independent time-varying features that serve as predictors for the main model, e.g., mobility and survey data. The only variables that we do not predict in the features model are static variables such as demographics. An example of a model output is shown in Fig. 1C, for New York state, specifically, the forecasted weekly cases for each of the four weeks following October 17th, 2020. Additional implementation of the multi-stage framework, details of model formulations, and model parameterization are described in detail in Appendix Section 2.1–2.3.

### Model evaluation

We conduct a robust evaluation of the model performance, explicitly assessing its performance as a function of space, time, and outbreak phase. All assessment is conducted over a long horizon (52 weeks, spanning all epidemiological weeks from August 2020 to August 2021), and evaluated using three different error metrics: a) Absolute Error (AE), b) Percentage Absolute Error (PAE), and c) Weighted Interval Scores (WIS).^19^ The definition of each error metric is described in Appendix Section 2.4. The first two metrics measure the accuracy of point predictions, while the last metric is intended to evaluate the model predictions as a probability distribution. For all experiments, we use JHU CSSE actual weekly reported cases and deaths^1^ as the ground truth data to compute the error metrics. While this analysis is retrospective, the evaluation is based on data that would have been available at the time of prediction, to align with the real-time forecasting constraints. For space constraints, the PAE results are presented throughout this section, and the WIS and AE results, when relevant, are provided in relevant sections throughout the Appendix. We compare our results to the CDC ensemble model,^19^ which we use as the benchmark because it has consistently proven to be the top performing model in the CDC COVID-19 Forecast Hub,^21^ among dozens of individually contributed models (ensemble members).

We also conduct sensitivity analysis to assess the contribution of each variable to the model performance, by evaluating different combinations of input features (Appendix Section 2.5). Due to time constraints and computational cost, the sensitivity analysis only applies to PAE and AE.

### Feature importance

We utilize an integrated gradients (IG) approach to investigate the role of each feature in predicting COVID-19 cases. IG assigns importance to features as attributions.^28^ It achieves this by integrating the gradients of the output with respect to the input along an arbitrary path from the baseline to the input data point. We apply the IG for the model with variant frequencies data and calculate the feature importance for each state at selected time points. The formulation of IG is described in detail in Appendix Section 2.6.

### Data

The proposed LSTM model is heavily data driven and trained using multiple disparate categories: epidemiological, mobility, survey, climate, demographic, and variant frequencies data. The time-varying data are available at a daily resolution for each US state. We rely on a combination of raw and derived metrics as inputs, which are listed in Table 1, and each is described in detail in Appendix Section 1.

**Table 1:** Summary of input data.

State-level data	Data processing	Data smoothing	Sources
**Epidemiological data**			
COVID-19 cases/deaths	Raw	7-day moving average	^1^
Growth rate of cases/deaths	Derived	7-day moving average	^1^
Vaccination coverage	Raw	7-day moving average	^32^
Hospitalization data	Raw	7-day moving average	^33^^,^^34^
**Mobility data**			
Importation risk	Derived	7-day moving average	^1^^,^^35^
Mobility ratio	Derived	7-day moving average	^35^
Visits ratio for 21 different destinations	Derived	Principal component analysis	^35^
**Survey data**			
COVID-like symptoms in community	Raw	Raw data has already been smoothed	^34^
**Climate data**			
Temperature (°C)	Raw	7-day moving average	^36^
Precipitation (mm/day)	Raw	7-day moving average	^36^
**Demographic data**			
Population	Raw	–	
Proportion of people over 65	Raw	–	^37^
**Variant frequencies data**			
Variant cases	Derived	7-day moving average	^38^

### Ethics

No animal or human experimentations involved in this study.

### Role of funders

The funders were not involved in study design, data collection, data analyses, interpretation of data, or writing of the manuscript.

## Results

Results for the LSTM model forecasted cases for 1-, 2-, 3- and 4-week forecasting windows, for every state in the US are presented in this section. Equivalent results for deaths forecasts are described in Appendix Section 3.8. We present our model performance as a function of time, space and different outbreak phases. We then conclude this section with results from a case study that supplements the input data streams with variant cases from available SARS-CoV-2 genomic surveillance data. The case study is conducted for a subset of states with the highest quality variant frequencies data, and the 2021 summer period, to align with the delta wave in the US. In Appendix Section 2.5 we present results from a sensitivity analysis conducted to assess the contribution of each variable in prediction. Finally, a feature importance analysis is included in Appendix Section 2.6 where we present the contribution of each feature at several critical time points, namely at the introduction of a new variant, the period of transition between dominant variants, and when the dominant variant reaching maximum proportion.

### Model performance across time

**Fig. 2:**
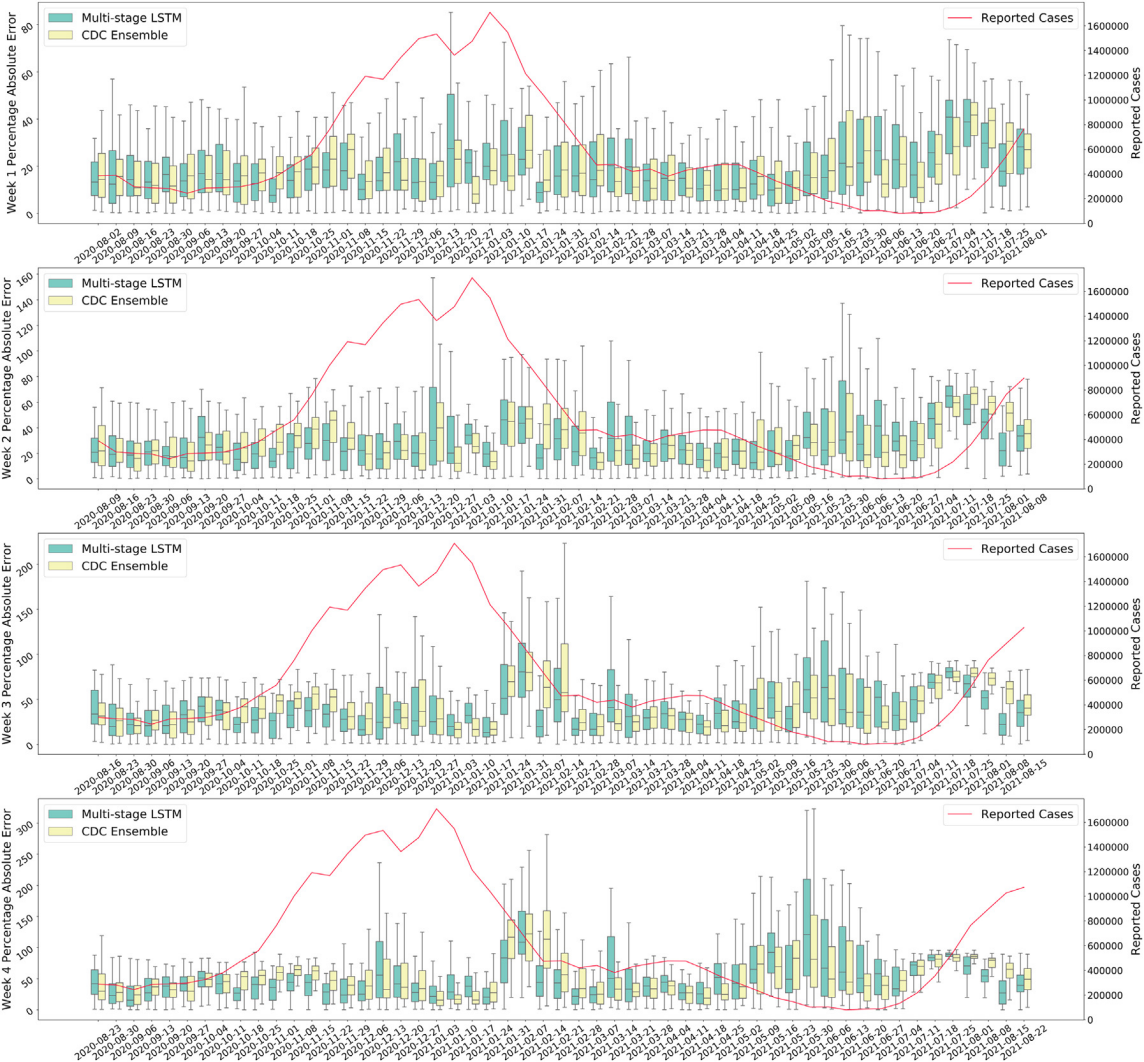
Comparison of model performance between the multi-stage LSTM Model and the CDC ensemble model based on PAE. Each pair of bar plots represents PAE distribution for all the states at a given week, where the green bar represents the error distribution for the multi-stage LSTM model, and the yellow bar represents the error distribution for the CDC ensemble model. The red curve represents the weekly reported cases at the national level. The left y-axis represents the PAE by different forecasting windows and right y-axis represents national level reported cases.

Fig. 2 illustrates the relative performance of the LSTM against the CDC ensemble model for each of the 52-week periods evaluated, for 1–4 week forecast windows, highlighting the performance variability over time. Each pair of bar plots represents PAE distribution for all the states at a given week, where the green bar represents the error distribution for the multi-stage LSTM model, and the yellow bar represents the error distribution for the CDC ensemble model. The red curve represents the weekly reported cases at the national level. The left y-axis represents the PAE by different forecasting windows and right y-axis represents national level reported cases.

For the time period evaluated the model consistently outperforms the CDC ensemble, especially during case surges, and for longer (3 and 4 weeks ahead) forecast windows. The average PAE across all states and weeks is 22%, 32%, 44% and 57% for the 1–4 week forecast windows, respectively. As the forecasting window increases, the variability in performance across states further increases, as indicated by the wider bars. Fig. 2 also reveals how the model performance varies with respect to the different waves of the pandemic. The model performance is relatively stable for the first five months of the study period (August 2020 to November 2020), but much more variable in performance in January 2021 and May 2021, which both correspond to periods when the cases transitioned from decreasing to more stable rates. The results for WIS and AE reveal consistent performance patterns, as illustrated in Appendix Section 3.1.

### Model performance across states

**Fig. 3:**
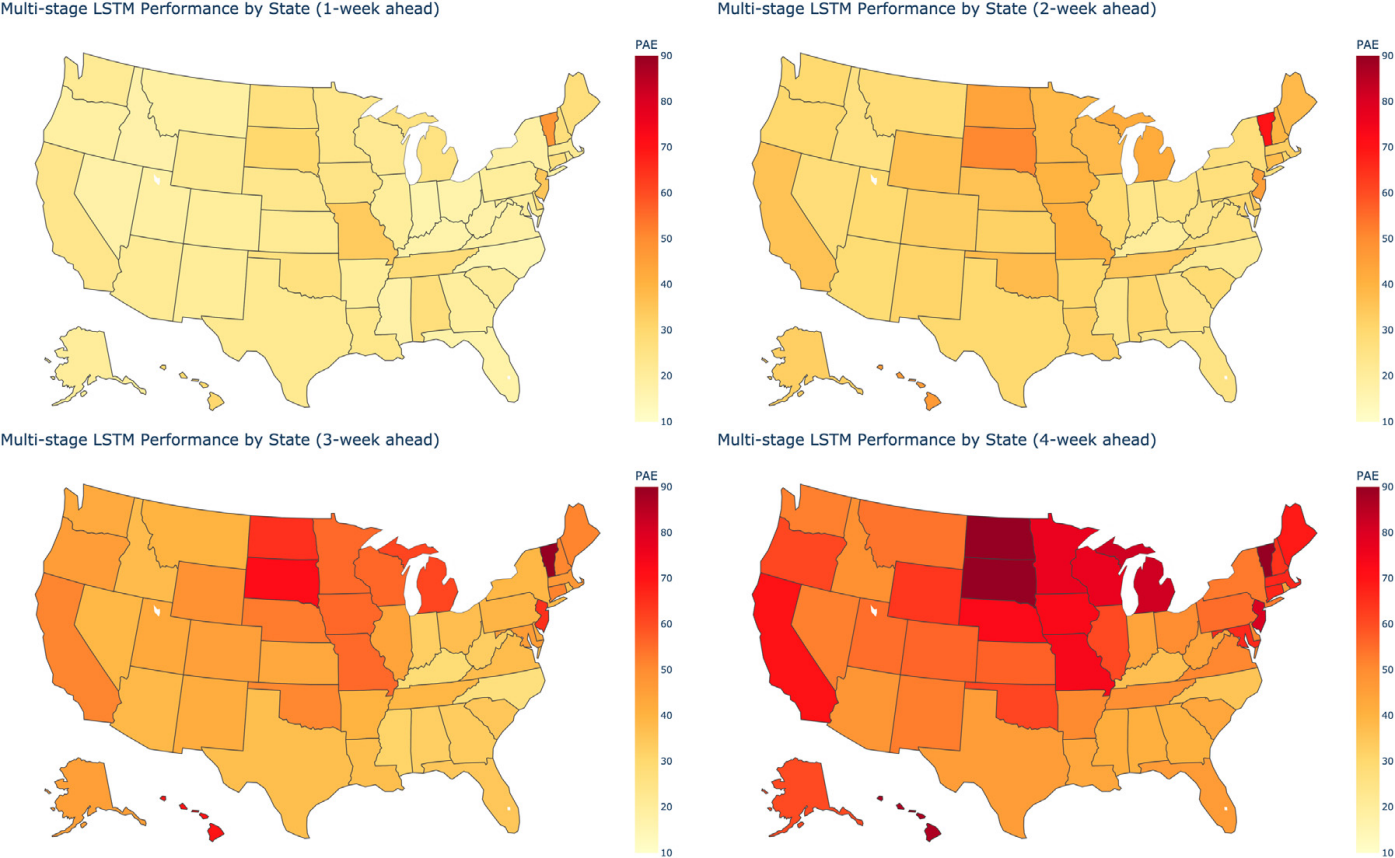
State-specific average model performance based on PAE (over all epidemiological weeks) for varying prediction windows of one-to four-week out predictions. The color scales represent the magnitude of the error metric; the scales of PAE are fixed in 10–90 range. The deeper color corresponds to larger error.

Fig. 3 illustrates the average performance over all 52 weeks, for each state, highlighting the performance variability across space. The color scales represent the magnitude of the error metric; the scales of PAE are fixed in 10–90 range. The deeper color corresponds to larger error. Equivalent evaluations for AE and WIS are included in Appendix Section 3.2. While there are no clear spatial patterns of model performance for 1-week ahead forecast, a spatial pattern becomes evident as the forecast window increases. For the 2 to 4-week forecast windows, the PAE is relatively larger for midwestern states and smaller for southeastern states. Reasons for this are addressed in the discussion section.

### Model performance by outbreak phase

In addition to examining performance variability over fixed space and time, we also evaluate the model performance as a function of the outbreak phase. To do this, we generate five outbreak phases based on the weekly average incidence growth rates and assign each state–week pair accordingly. We apply 5-quantiles clustering according to the relative magnitude of growth rate, the five groups are classified as: 1) fast increasing (growth rate above 0.017); 2) slightly increasing (growth rate between 0.005 and 0.017); 3) flat (growth rate between −0.004 and 0.004); 4) slightly decreasing (growth rate between −0.016 and −0.004); and 5) fast decreasing (growth rate below −0.016). The assignment of the weeks to categories is presented in Appendix Fig. S23. After the phase category assignment, we evaluate the performance for all state–week pairs in each of the five phase groups independently.

**Fig. 4:**
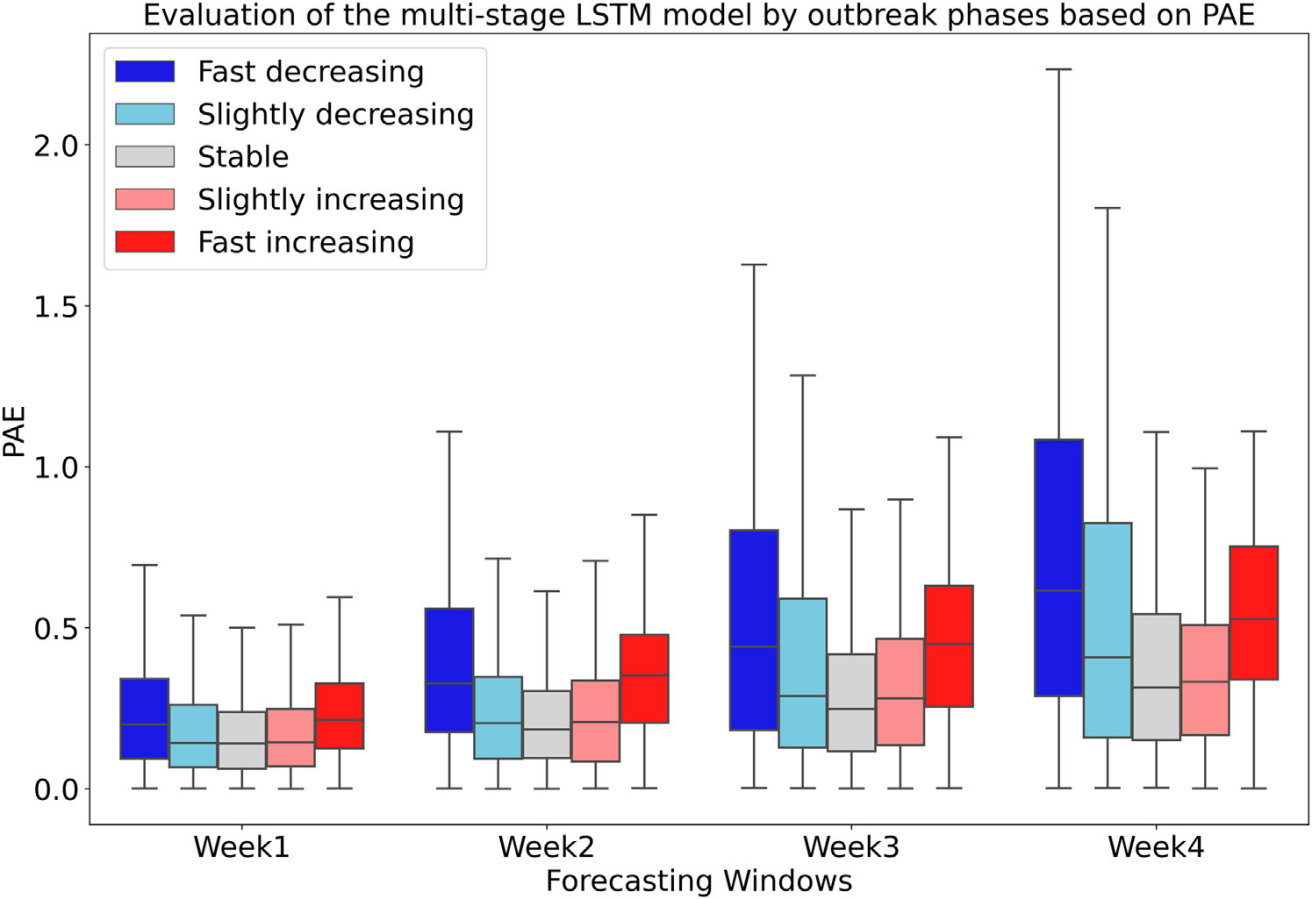
Evaluation of the multi-stage LSTM model by outbreak phases based on PAE. The colors represent different outbreak phases, and each bar represents the distribution of PAE in corresponding outbreak phases.

Fig. 4 shows the model performance of the multi-stage LSTM model by different outbreak phases, the colors represent different outbreak phases, and each bar represents the distribution of PAE in corresponding outbreak phases. This result reveals that the model performs best in the stable period and has the highest variability when cases change rapidly, consistent with the same evaluation for the CDC Ensemble model (Appendix Fig. S24). Equivalent evaluation based on WIS are shown in Appendix Figs. S25 and S26. In addition to evaluating the LSTM and CDC Ensemble model separately, we also compare both models under each outbreak phase (see Appendix Section 3.6). As shown in Appendix Figs. S27 and S28, when growth is classified as *fast increasing,* the multi-stage LSTM model outperform the CDC ensemble model over 60% of the time for all forecast windows. For the *slightly increasing* and *fast decreasing* periods, our model slightly outperforms the CDC ensemble. However, the performance of the model is lower than the CDC ensemble during periods of outbreak stability and slight declines (e.g., December 2020 and May 2021).

### Case study with variant frequencies data

The US has experienced multiple waves of incident cases, often driven by new variants. In this case study, we conduct a retrospective analysis to explore the value of including variant cases from available SARS-CoV-2 genomic surveillance data in improving COVID-19 outbreak prediction using our proposed modeling framework, based on the hypothesis that variant frequencies data may act as a signal for forthcoming changes in transmission patterns and therefore help improve prediction accuracy.^29^ Here we focus on forecasting state-level confirmed cases in the US, capturing the wave caused by the Delta and Omicron variant. We implement the analysis for the 39 selected states that sequenced at least 5% of reported cases from May 1 to August 31, 2021. We generate new variant-specific case time series (as the product of the daily proportion and total daily cases reported), which are used as inputs in the model. Details of the variant frequencies data preprocessing are documented in Appendix Section 1.6. For Delta wave, we select the top three variants with the highest proportion during June and September 2021 as new variant-specific time series, i.e., Delta, Gamma, and Alpha. In addition, we also create a fourth time series (“other”) representing the sum of all other circulating SARS-CoV-2 lineages. The inclusion of “other” category enables us to capture the introduction of new variants, in addition to other known circulating variants. We apply the same approach to generate variant-specific time series for the Omicron wave between December 1, 2021, and January 1, 2022. When applying the model, the selection of the variant-specific time series can be adjusted dynamically, based on the most recent data.

**Fig. 5:**
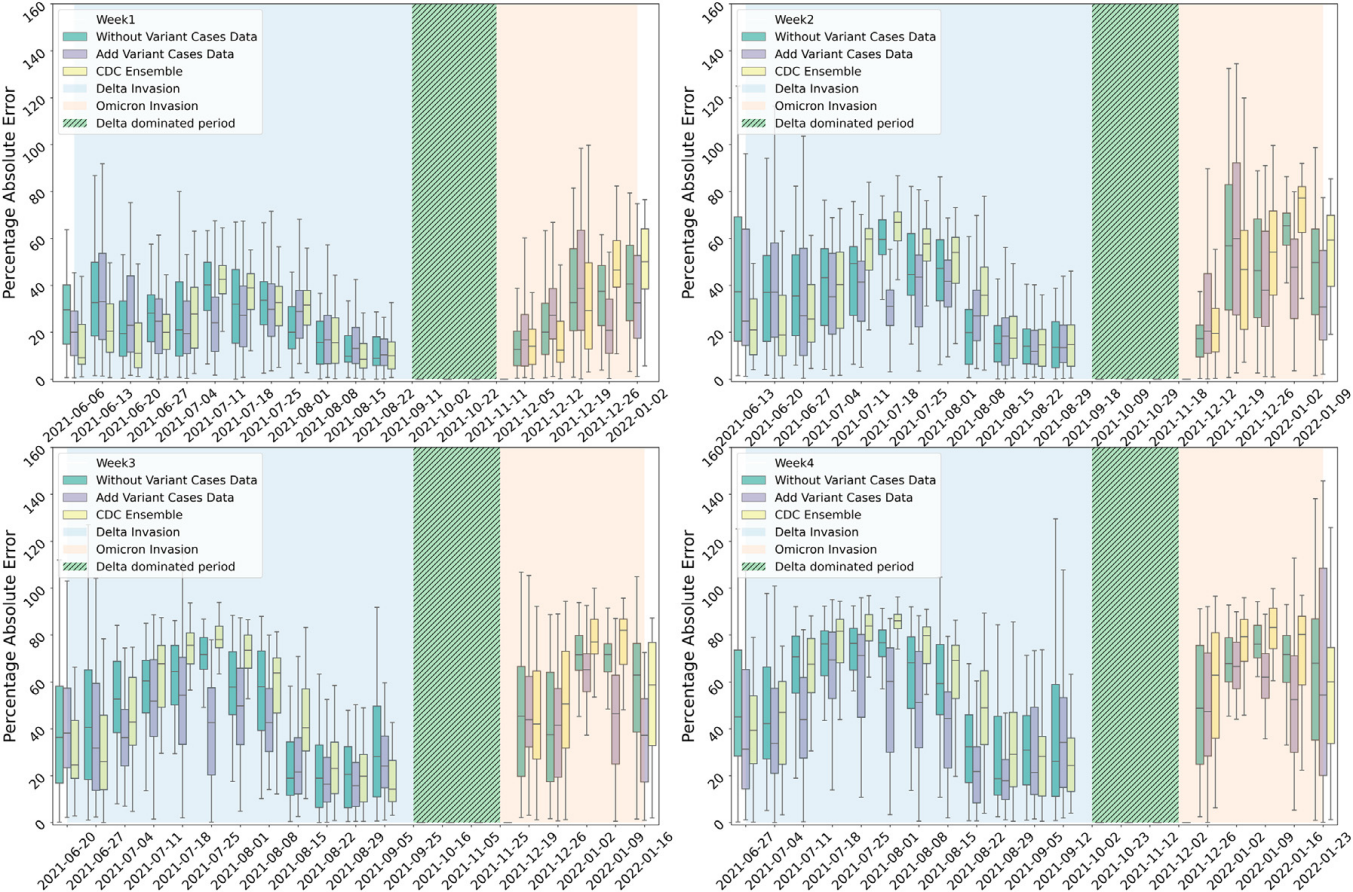
Model performance based on PAE for three different models: (a) Multi-stage LSTM model without variant cases data, (b) Multi-stage LSTM model with variant cases data and (c) CDC Ensemble model. The x-axis is the week that the predictions are made on. Each pair of bar plots represents PAE distribution for the selected states at a given week, where the green bar represents the error distribution for the multi-stage LSTM model without variant cases data, purple bar represents the error distribution for the multi-stage LSTM model with variant cases data, and the yellow bar represents the error distribution for the CDC ensemble model. The blue region represents the period of Delta invasion, the shaded green region represents Delta dominated period (proportion of Delta reaches 100%), and the orange region represents the period of Omicron invasion.

Fig. 5 illustrates the results for three different models: (a) Multi-stage LSTM model without variant cases data, (b) Multi-stage LSTM model with variant cases data and (c) CDC Ensemble model. The x-axis is the week that the predictions are made on. Each pair of bar plots represents PAE distribution for the selected states at a given week, where the green bar represents the error distribution for the multi-stage LSTM model without variant cases data, purple bar represents the error distribution for the multi-stage LSTM model with variant frequencies data, and the yellow bar represents the error distribution for the CDC ensemble model. The blue region represents the period of Delta invasion, the shaded green region represents Delta dominated period (proportion of Delta reaches 100%), and the orange region represents the period of Omicron invasion. Results from the case study suggests that the inclusion of variant cases data have varying levels of impact on the model performance, dependent on the time period, but critically, are shown to improve performance in the early growth stage of a newly introduced variant. Furthermore, results from the feature analysis present in Appendix Figs. S11–S16 highlight the critical role these variant-specific time series play in these critical phases of the outbreak, specifically when a new variant is emerging to be the dominant variant in circulation, the variant-specific data input is the most significant feature in the model. This holds true for both the Delta and Omicron introductions. Other specific performance trends are noted in the discussion section. The results based on AE and WIS are shown in Appendix Section 3.7.

Notably, this study is retrospective, and therefore is not subject to the real-time reporting limitations of SARS-CoV-2 variant frequencies data from sequences COVID-19 cases. Specifically, the average time lag in variant frequencies data reporting is 26 days,^30^ whereas we assume data is available with a seven day lag. While not feasible at present, this study highlights the potential value of timely and open virus genomic surveillance as a pandemic forecasting tool.

### Model selection

We conduct sensitivity analysis to assess the importance and contribution of various input features and training periods to identify the best performing model. We assign features into four categories (epidemiological, mobility, survey, and climate data). The complete set of features considered, and category assignment are listed in Table 1. Four models are constructed which include different combinations of available features, namely 1) a simple basis model with only epidemiological data, 2) a model with epidemiological and mobility data, 3) a model with epidemiological, mobility and survey data, and 4) a model with all features. We further conduct the equivalent model comparison for two discrete time periods aligning with pre and post available vaccines, specifically divided on February 1, 2021, approximately when vaccination roll out began in the US. The results comparing the performance of these four models for the entire period and two discrete periods are shown in Appendix Figs. S9 and S10, respectively. The results reveal that the model with epidemiological, mobility, and survey data has the best overall performance. However, the contribution of each input feature can vary across time; this is expanded upon in the discussion section. Finally, the analysis performed for COVID-19 deaths as a response variable is presented in Appendix Fig. S26, where model 3) and 4) have similar performance. Additional sensitivity analysis on model's input parameters is included in the Appendix Section 2.5.

## Discussion

### Spatiotemporal variability of model performance

Our analysis reveals a high variability in model performance as a function of the forecast window, chronological time and space. The performance over the 52 weeks evaluated is closely tied to the observed outbreak dynamics, and Fig. 2 highlights the impact of rapidly changing dynamics on the model performance. The model performs worse around the inflection period (especially when cases' trend changes from decreasing to stable), and gradually improves as case (and death) rates stabilize. In terms of spatial patterns, for the time period evaluated model is more accurate in eastern and southeastern states, compared with midwestern states. This pattern is further confirmed by comparing the model performance with the CDC ensemble model. This spatial pattern can be partially explained by the difference in case trends across these regions. Specifically, during October 2020 to December 2020, midwestern states experienced the fall COVID-19 wave ahead of most of the country. Specifically, midwestern states started to show a decreasing trend while cases were increasing elsewhere (see Appendix Section 3.3). Because the model is trained using the data for all states for each prediction period, the predictions will be guided by the most dominant trend, and the model may underperform for any states not experiencing the same patterns. As an extension of this work, one could develop group-specific models through a cluster-based training setup or a more deliberate design of loss function, and as such, generate forecasts for each sub-group. Additionally, as expected, the model performance decreases as the forecasting window increases. This outcome is partially an artifact of the multi-stage nature of the modeling framework, which is sensitive to accumulative uncertainty in the input data and error propagation in the model outputs; e.g., predictions generated for each week are used as inputs for the following week's prediction. Therefore, in periods of high instability, the one-week ahead predictions can be more erroneous, thus the error will be larger for longer forecast windows relative to the same forecast window in more stable periods. Overall, the observed spatial and temporal variability in model performance highlights the importance of identifying and communicating the optimal performance conditions for a given model before it is shared publicly or relied upon by decision makers.

### Model performance varies by outbreak phase

In Fig. 2, the LSTM model is shown to perform consistently better than the CDC ensemble model in the periods of rapid outbreak growth (e.g., October 2020–November 2020, July 2021) and decline (e.g., January 2021). To further explore model applicability, we evaluated model performance as a function of the outbreak phase, namely periods of growth, decline or stability, which were designated by five discrete categories. For the nine most populated states, most of the weeks in fall 2020 and summer 2021 are assigned to either fast or slightly increasing phase categories (Appendix Fig. S23). The results highlighted in Fig. 4 reveal the LSTM model to perform best in stable periods, and poorest in periods of extreme growth and decline. However, critically, the comparison of our LSTM model against the CDC Ensemble as a function of the outbreak phase, presented Appendix Figs. S27 and S28, reveals that the multi-stage LSTM model performs relative better during the most critical phases of fast growth and fast decreases. This variation in forecasting accuracy during the rapidly changing outbreak phases is consistent with COVID-19 forecasting literature.^21^ Future work should consider relaxing continuous forecasting outputs, and focusing on categorical predictions, which may be able to be generated more accurately and reliably. Our analysis also highlights that model selection should consider model performance relative to the phase of the outbreak, in addition to the fixed time and location the model is applied to.

### Model evaluation is sensitive to performance metric chosen

A major focus of this analysis is to explore the how model performance relates to the metrics chosen for evaluation. As illustrated in the Appendix Section 3.1, the performance of the LSTM and CDC ensemble model can vary significantly, dependent on the error metric selected. This occurs due to the way the metrics are mathematically defined (Appendix Section 2.4), in particular, whether they are normalized to account for potentially large variations in the magnitude of the predictor variable or not, as well as how they account for uncertainty bounds. For example, AE has a positive correlation with confirmed case counts, therefore the states and outbreak periods with the highest reported case values will have higher AE scores; this is the case for California, New York, and Florida (Appendix Fig. S20). In contrast, PAE is normalized by case levels, and is therefore more likely to have a higher relative value when case rates are low because small variabilities in the estimated versus observed incidence rate will be amplified. This behavior is illustrated during summer 2021 in states with lower populations like Maine, New Hampshire, and Vermont, when the weekly confirmed cases are below 50 (Fig. 3). For all forecasting windows, the results are shown to be sensitive to the error metric chosen, and critically, the selection of the best performing model for a given state is dependent on the metric chosen for evaluation. However, as the forecasting window increases, the LSTM model appears to consistently outperform the CDC ensemble model for the southeastern states (i.e., Virginia, North Carolina, South Carolina) according to all metrics. This analysis highlights the need to consider multiple metrics in evaluating models, in order to improve model selection and robustly assess model performance.

### Model sensitivity to input data streams

Results from the sensitivity analysis to assess the importance and contribution of various input features revealed the best performing model included all the features except climate data. Our analysis reveals that a model solely reliant on epidemiological data performed worst, while adding mobility and survey data reliably improved model performance, especially for longer forecasting windows. These results support the inclusion of preprocessed mobility variables and real-time survey variables in learning model frameworks such as the proposed LSTM model. While the epidemiologic, survey and mobility variables revealed similar roles across the entire study period, and each of the separate periods evaluated, the role of climate variables is less clear. The inclusion of climate variables did not initially appear to improve predictive capability (when considered across the entire study period), however, when we divided the study period into two discrete periods, the role of the climate data changed. For the period between August 2020 and February 2021, the inclusion of climate data did not improve the model performance, however during the second phase of the study period, between February and August 2021, the inclusion of climate variables increased the model performance (Appendix Fig. S9). These results suggest a differing role of climate on COVID-19 transmission in the first and second year of the pandemic, which aligns with other literature.^31^ We hypothesize in the first year of the pandemic factors other than climate, such as behavior and underlying population immunity, dominated the role of climate, and/or the role of climate is being captured indirectly through other predictors (e.g., higher temperatures lead to behavioral changes which can be captured through the survey and mobility data sets). While this preliminary analysis sheds some light on the possible role of climate and seasonality of COVID-19, this is an area in need of further research.

### Inclusion of variant frequencies data improves model performance

The case study, designed to capture the impact of new variant introductions on outbreak dynamics, highlights the value of using variant frequencies data in short-term epidemiological forecasting, specifically with regards to early identification of inflection points. Due to differences in relative infectivity and underlying population immunity, the Delta and Omicron waves occurred over different timescales; the Delta variant took around two months to increase from 0% to 100% of the reported variant proportion, while the Omicron variant reached 100% in half this time. These differences led to variable model performance patterns, however for both, the variant data provided clear benefit for model performance during the emerging period. For the Delta wave, the added value of including variant case data was evident within two weeks after the average proportion of the Delta variant was above 15% for most of the 39 states included in the cases study. Specifically, the LSTM model with variant cases data performed better than both the reference LSTM model (without the variant cases data) and the CDC ensemble model for predictions between epidemiological weeks June 20, and July 25, 2021, especially for the longer three- and four-week forecasting windows. This is approximately the period when the dominant variant switches from Alpha to Delta (Appendix Fig. S6). The results for the Omicron wave further confirm this performance pattern. The multi-stage LSTM model with variant cases data begins to outperform the other two reference models just two weeks after the majority of states first reported Omicron cases. However, the model with variant case data is not always superior; for both the Delta and Omicron waves the model with the variant data lagged the other reference models once the variant proportion reached 100%, respectively. A possible explanation for this is that when the Delta or Omicron variant proportion reached 100%, the proportion of other variant specific cases suddenly dropped to zero, and the multi-stage LSTM model requires a learning period to adapt to this change in the input data stream (Fig. 5).

Results from the feature importance analysis provides additional evidence for the significant role of variant data during critical windows of the COVID-19 pandemic. During the periods when there is a transition between dominant circulating variants (Appendix Figs. S12 and S15), the emerging variant cases become the most dominant feature guiding the model predictions. However, outside of this window the variant data is not as important. Immediately after a new variant is identified (Appendix Figs. S11 and S14) and after the new variant proportion reaches 100% (Appendix Figs. S13 and S16), the new variant cases have minor contributions compared to other features. Additionally, the feature analysis results more broadly highlight that the contribution of each feature varies substantially from week to week, with no predictable pattern for feature contribution. This finding highlights the complexity of COVID-19 forecasting and further justifies the non-linear, deep learning methodology we chose in this work.

### Limitations

There are several limitations to this study, primarily resulting from data issues, and imposed methodological constraints. Most critically, there are challenges posed by the quality and availability of the data relied upon, for both the health outcomes data sets used to represent ground truth, as well as the input data streams. Given the intended real time use of this framework, the best available data at the time of generating the forecast were used to both train and evaluate the model, and as such, unresolved anomalies, biases and inaccuracies in the data directly affect performance. Further data quality issues such as spatiotemporal biases, sample size and data gaps also posed challenges, and were more prevalent in the data sets used to capture human behavior, e.g., survey data. In addition to quality of the data used, certain critical features are excluded from the model, such as government policies and policy compliance rates, as well as other behavioral data. Future work should explore the inclusion of these additional data sources to further enhance model performance. In addition to data issues, the LSTM model is fully empirical, i.e., it does not have a mechanistic component, therefore the actual infection dynamics are not constrained by feasible outbreak scenarios, which can result in unrealistic predictions. The empirical nature of the model also constrains the forecasts to previously observed transmission patterns (within the training time window); thus, the model will perform poorly when the transmission dynamics dramatically differ (exceed) from prior behavior.

### Conclusion

We introduced a flexible deep learning framework that utilizes a broad set of data types (epidemiological, mobility, survey, climate, demographic, and variant frequencies) to forecast COVID-19 cases and deaths in real time. The novel multi-stage forecasting routine uses an iterative approach, building on one stage's outputs to generate the next stage's predictions. We applied our framework for the United States at a weekly temporal resolution and state-level spatial resolution, for a four-week planning horizon. We evaluated our model at each epidemiological week over the 52-week period between August 2020–August 2021, and quantified performance using three different error metrics. We further break down the performance as a function of outbreak phases, location, time, and forecasting window. While the model is shown to perform well in multiple settings, the results from this analysis illustrate a variable performance of the model across the considered dimensions. This variability is driven by the complex, uncertain and evolving role of the critical contributing factors that drive COVID-19 transmission dynamics. This includes, for example, changes behavior, immunity, climate, the environment, and viral dynamics. Additionally, our case study reveals that the model can learn from simple variant proportion data within two weeks after a new variant is first reported. During this critical window the model heavily relies upon variant cases data and performs better, highlighting the value of using variant frequencies data in short-term epidemiological forecasting. However, as the new variant proportion reaches 100%, the variant cases data gradually lose their additional value. Based on these findings, forecasting models should be accompanied with specifications on the conditions under which models performs best (and worst), in order to maximize their value and utility in aiding public health decision making. Extensions of this work include applying it at higher spatial resolutions (e.g., at the county level), and for predicting other response variables (e.g., hospitalization rates). Further, we selected a simple LSTM as the model's building block since it is a state-of-art framework for processing time dependent data, however, rigorous inter-comparisons with other deep learning techniques should be conducted.

## Contributors

LG, HD, and ED contributed to the conceptualization and design of the study. HD and ED collected the data and conducted the analysis. HD led the writing of the original draft. HD, ED, HB, MP, NG, and LG edited the manuscript, discussed results, and provided feedback regarding the manuscript. LG supervised the study and acquired funding. HD and ED have verified the underlying data. All authors had full access to the data and approved the manuscript for publication.

## Data sharing statement

Code for training and the trained models are publicly available on GitHub at https://github.com/hongru94/multi_stage_LSTM.

## Declaration of interests

The authors declare no potential conflicts of interest.

## Appendix A Supplementary data

Supplementary_Appendix_revision_clean Figs. S1–S34 and Tables S1 and S2
