# Supplementary material for: Incorporating variant frequencies data into short-term forecasting for COVID-19 cases and deaths in the USA: a deep learning approach
Source: eBioMedicine. 2023 Feb 21;89:104482. doi: 10.1016/j.ebiom.2023.104482 (PMC9943054; doi:10.1016/j.ebiom.2023.104482)
Supplement: Supplementary_Appendix_revision_clean Figs. S1–S34 and Tables S1 and S2 [file mmc1.docx]

**Supplementary Appendix**

Table of Contents

[1. Data and Preprocessing 2](#_Toc120803859)

[**1.1 Epidemiological data** 2](#_Toc120803860)

[**1.1.1 Cases and Deaths** 2](#_Toc120803861)

[**1.1.2 Case and Death Growth Rate** 2](#_Toc120803862)

[**1.1.3 Vaccination data** 2](#_Toc120803863)

[**1.1.4 Hospitalizations data** 2](#_Toc120803864)

[**1.2 Mobility derived metrics** 2](#_Toc120803865)

[**1.2.1 Mobility Ratio (MR)** 3](#_Toc120803866)

[**1.2.2 Importation risk (IR)** 3](#_Toc120803867)

[**1.2.3 Purpose-specific visits (VR)** 4](#_Toc120803868)

[**1.2.4 Principal component analysis of purpose-specific visits metrics** 5](#_Toc120803869)

[**1.3 COVID-19 symptoms survey data** 7](#_Toc120803870)

[**1.4 Climate data** 8](#_Toc120803871)

[**1.5 Demographic data** 8](#_Toc120803872)

[**1.6 SARS-CoV-2 variant frequencies data** 8](#_Toc120803873)

[2. Supplementary method 12](#_Toc120803874)

[**2.1 An example implementation of the multi-stage LSTM model** 12](#_Toc120803875)

[**2.2 Formulations of LSTM model** 12](#_Toc120803876)

[**2.3 Model parameterization** 13](#_Toc120803877)

[**2.4 Model evaluation metrics** 13](#_Toc120803878)

[**2.5 Model Selection** 13](#_Toc120803879)

[**2.6 Feature Importance** 15](#_Toc120803880)

[3. Supplementary results 22](#_Toc120803881)

[**3.1 Model performance across time by AE and WIS** 22](#_Toc120803882)

[**3.2 Model performance across states by AE and WIS** 24](#_Toc120803883)

[**3.3 Reported Cases Trend by Region** 25](#_Toc120803884)

[**3.4 An example of classification for different outbreak phases** 27](#_Toc120803885)

[**3.5 Model Performance by Outbreak Phase** 28](#_Toc120803886)

[**3.6 Compare model performance by outbreak with the CDC Ensemble model** 30](#_Toc120803887)

[**3.7 Comparing model performance after adding genomic cases data by AE and WIS** 32](#_Toc120803888)

[**3.8 Results of weekly deaths forecasting** 34](#_Toc120803889)

[Reference 39](#_Toc120803890)

# **1. Data and Preprocessing**

The proposed LSTM model is trained using multiple disparate categories of data including epidemiological, mobility, survey, climate, vaccine coverage, demographic, and genomic data. The time-varying data are all available at a daily resolution, and state spatial resolution. We use a mixture of preexisting and generated metrics as input; all the variables and their corresponding categories are summarized in the table 1 in the main manuscript, and described in detail below:

## **1.1 Epidemiological data**

Previous COVID-19 modeling studies have relied upon a wide range of data types, with epidemiological data being the most central to the efforts. Potential epidemiological variables include reported cases and deaths, unreported or undetected infections and fatality, incidence rate, mortality rate, case-fatality ratio, growth rates, testing data, vaccination coverage, and hospitalization data. ^1–3^

## **1.1.1 Cases and Deaths**

Our study utilizes the county-level, daily reported COVID-19 case, death and vaccination data ranging from April 1, 2020 to August 31, 2021 as its primary epidemiological inputs. The data is sourced from the Center for Systems Science and Engineering (CSSE) at Johns Hopkins University. ^4^ This dataset serves as the gold standard for reliable and official reported state- and county-levels cases and deaths for the US. The start date of May 1, 2020 was chosen to minimize the possible effect of underreporting at the early stages of the pandemic. The raw case and death data are aggregated to the state level. A 7-day moving average is used to address noise due to reporting issues and variable day-of-week patterns.

## **1.1.2 Case and Death Growth Rate**

The smoothed timeseries are also used to derive additional epidemiologic parameters used as latent variables in our modeling framework, namely growth rates and incidence rates. The growth rates (GR) for cases and deaths are calculated as follows:

$${GR}_{i}^{t}=\log\left( C_{i}^{t} \right)-log(C_{i}^{t-1})$$

Where $C_{i}^{t}$ represents the smoothed cases or deaths for state $i$ on day $t$. The case and death incidence rates are computed by normalizing the data by population, to generate daily cases and deaths per 100,000 persons, for each state.

## **1.1.3 Vaccination data**

Vaccine induced immunity is considered to be an essential strategy for reducing COVID-19 harm. In our model we utilize state-level vaccination data from Johns Hopkins CRC,^5^ which is collected from the US CDC Vaccine Tracker ^6^ and local health agencies. We adopt its daily state-level complete vaccination data normalized by population as one of the inputs.

## **1.1.4 Hospitalizations data**

The U.S. Department of Health & Human Service (HHS) publishes datasets “COVID-19 Reported Patient Impact and Hospital Capacity by State” via healthdata.gov. ^7^ The original dataset contains multiple columns that break the patient and hospital resources into several categories. We use cleaned COVID-19 hospitalization provided by the Delphi group at Carnegie Mellon University API. ^8^ We used 7-day moving average smoothed “inpatient_bed_used_covid” time series for our death’s prediction model.

## **1.2 Mobility derived metrics**

Previous studies have shown that aggregate human mobility patterns can be used to evaluate the impact of certain non-pharmaceutical interventions on the spread of COVID-19. ^9–12^ However, the role of such aggregate mobility data in predicting COVID-19 transmission patterns is complex, highly variable over time and space, and notably diminishing since Spring 2020. ^13,14^ Therefore, we conduct extensive data analysis and modeling, to generate novel mobility related variables that explicitly consider trip purpose in addition to broader mobility patterns and incorporate these new mobility-derived metrics into our modeling framework.

For the purposes of this study we obtained aggregated and anonymized mobility data from Safegraph,^15^ a company that provides location data from mobile applications. We generate multiple mobility metrics from the provided weekly patterns and places datasets ^16,17^ as described below.

### **1.2.1 Mobility Ratio (MR)**

We compute a mobility ratio (MR) as a proxy for aggregate mobility movement at population level.^9^ To generate MR, we utilize the following raw point of interest (POI) variables:

$v_{i}^{t}$: the number of visits to POI $i$ on day $t$.

$v_{i}^{w}$: the number of visits to POI $i$ during week $w$.

$r_{i}^{w}$: the number of visitors to POI $i$ during week $w$.

$r_{ji}^{w}$: the number of visitors to POI $i$ with home location in census block (CBG) $j$ during week $w$.

$D_{i}^{w}$: the number of devices residing in given CBG $i$ during week $w$.

where $t$ to represent daily resolution and $w$ to represent weekly resolution. The raw data include the number of visits to each POI at daily resolution. However, there is a gap in that the origins of those visits are missing. Hence, additional data preprocessing is needed to estimate origin-destination metrics. For each POI, we first compute the number of visits per visitor $\bar{v}_{i}^{w}$ as $v_{i}^{w}$ divided by $r_{i}^{w}$, and we assume that $\bar{v}_{i}^{w}$ is a constant for all visitors to POI $i$ during week $w$. Then we aggregate the visitor’s home location to state-level, and normalize the counts by the state population (${pop}_{c}$) as:

$$\hat{r}_{ci}^{w}=\left( \sum_{j \in c} r_{ji}^{w} \right)\times\frac{{pop}_{c}}{\sum_{j \in c} D_{j}^{w}}$$

Here $\hat{r}_{ci}^{w}$ indicates the normalized number of visits from state $c$ to POI $i$ during week $w$. The probability $p_{i}^{t}$ that a visit during week $w$ happens during day $t$ is calculated as $v_{i}^{t}$ divided by $v_{i}^{w}$, and we assume this distribution holds for visitors from any state. The daily mobility metric $\hat{r}_{cs}^{t}$ from state c to state s can be estimated as:

$$\hat{v}_{cs}^{t}=\sum_{i \in s} (\hat{r}_{ci}^{w} \times\bar{v}_{i}^{w} \times p_{i}^{t})$$

Note that SafeGraph’s data is collected based on device’s home location, so more rigorously, $\hat{v}_{cs}^{t}$ should be interpreted as number of visits with visitors’ home location in state $c$ to state $s$ on day $t$.

The MR is then defined as:

$${MR}_{c}^{t}=\frac{\sum_{c\neq s} \hat{v}_{cs}^{t}+\sum_{c\neq s} \hat{v}_{sc}^{t}+\hat{v}_{cc}^{t}}{\sum_{c\neq s} \hat{v}_{cs}^{t_{0}}+\sum_{c\neq s} \hat{v}_{sc}^{t_{0}}+\hat{v}_{cc}^{t_{0}}}$$

Where $\hat{v}_{cs}^{t}$ represents the number of trips from location state $c$ to state $s$ on day $t$. $t_{0}$ represents the baseline time period, which is chosen as the average day of week (e.g., Monday) over the month of February 2020, and $\hat{v}_{cs}^{t_{0}}$ represents the baseline trip rate between locations $c$ and $s$.

### **1.2.2 Importation risk (IR)**

In addition to the general mobility trend variable (MR), we generate an importation risk (IR) variable to capture the potential risk of infected visitors arriving at a given destination. This variable combines the real time mobility data and regional case incidence rates at the origin of travel to generate an incidence-weighted travel risk posed to the destination location. The formulation is defined as follow:

$${IR}_{s}^{t}= \sum_{c} I_{c}^{t}\hat{v}_{cs}^{t}, i\neq j$$

$I_{s}^{t}$ represents the 7-day moving average of reported case incidence rate in trip origin state $i$ on day $t$, and $\hat{v}_{cs}^{t}$ is the same as described above.

### **1.2.3 Purpose-specific visits (VR)**

For each POI, SafeGraph also provides a NAICS (North American industrial classification system) code, which clusters the POIs into different categories based on their primary activity. Previous study^11^ has listed top 50 categories accounting for the largest fraction of visits, we select top 21 as our target destinations, where each type of POI consists at least 1% of overall visits. We generate ${VR}_{cp}^{t}$ for 21 types of POI ($p=21$), each one of them is a time series on a daily basis.

For each selected type of POIs ($p$), we estimate the mobility metric $\hat{v}_{cp}^{t}$ from state c to POI type p as:

$$\hat{v}_{cp}^{t}=\sum_{i \in p} (\hat{r}_{ci}^{w} \times\bar{v}_{i}^{w} \times p_{i}^{t})$$

All the selected POI categories are listed below:

Supplementary Table 1: The 21 selected POI categories and their NAICS code.

| **POI categories** | **NAICS code** |
| --- | --- |
| Full-Service Restaurants | 722511 |
| Limited-Service Restaurants | 722513 |
| Elementary and Secondary School | 611110 |
| Other General Merchandise Store | 452319 |
| Gas Station | 4471 |
| Fitness and Recreational Sports Center | 713940 |
| Grocery Store | 4451 |
| Cafes & Snack Bars | 722514, 722515 |
| Hotels and Motels | 721110 |
| Religious Organizations | 813110 |
| Nature Parks and Other Similar Institutions | 712190 |
| Hardware Store | 444130 |
| Department Store | 452210 |
| Child Day Care Service | 624410 |
| Offices of Physician | 6211 |
| Pharmacies and Drug Store | 446110 |
| Sporting Goods Store | 451110 |
| Automotive Repair and Maintenance | 8111 |
| Used Merchandise Stores | 453310 |
| Colleges, Universities, and Professional Schools | 6113 |
| Convenience Store | 445120 |

An example visualization of all purpose-specific visits metrics for New York State is shown below:


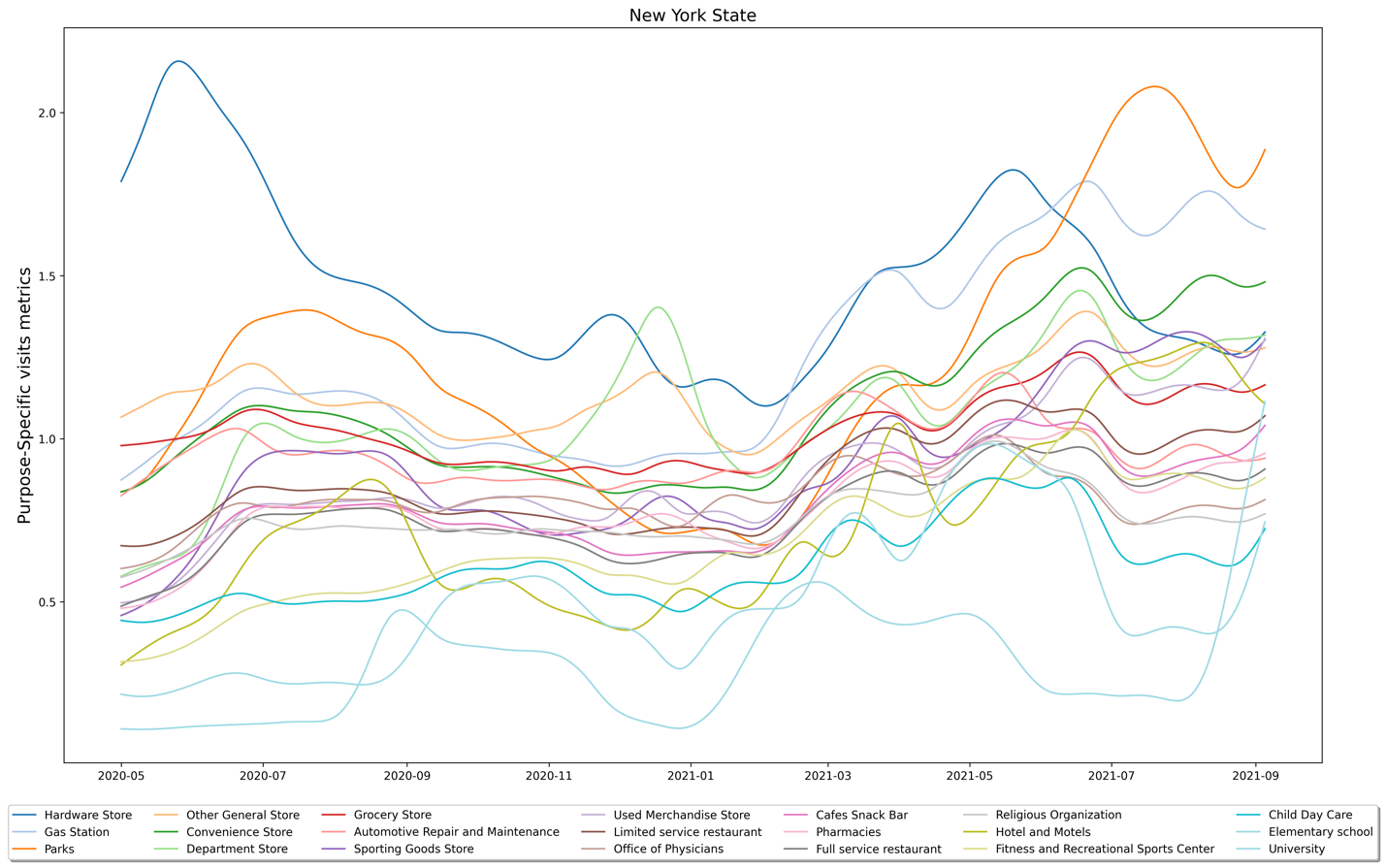


Supplementary Figure 1: GAM smoothed timeseries of ${VR}_{cp}^{t}$ for New York State from May 2020 to September 2021.

Similar to the definition of MR, we define a visit ratio (VR) for each pair of locations (states or counties) and select types of points of interest (POIs). This variable is designed to disaggregate the mobility data by trip purpose, and explicitly considered different travel purposes (work, school, restaurant visits, etc.) within the modeling framework:

$${VR}_{cp}^{t}=\frac{\hat{v}_{cp}^{t}}{\hat{v}_{cp}^{t_{0}}}$$

Here, $\hat{v}_{cp}^{t}$ are the estimated daily visits from location (a state or county) $c$ to selected POI $p$. Again, $t_{0}$ represents the baseline time period. ${VR}_{cp}^{t}$ indicates how frequent people visit certain types of destinations relative to the baseline. ${VR}_{cp}^{t}$ for New York State are shown in Appendix Figure 1, where ${VR}_{cp}^{t}$ are smoothed with Generalized Additive Model (GAM). The SafeGraph’s data was updated daily during 2020; however, in 2021, the data is updating once a week on every Wednesday.

### **1.2.4 Principal component analysis of purpose-specific visits metrics**

To avoid the highly correlated features and increase computational efficiency, we applied the principal component analysis (PCA)^18^ to all VR variables and select the first five principal components as inputs for the model. By doing this, we could first avoid using similar features that are highly correlated; second, the computational cost is reduced. Here we presented one example of this data preprocessing routine for the first week of September 2021. The average correlations between all the visits metrics across 50 states are show in the Appendix Figure 2 below:


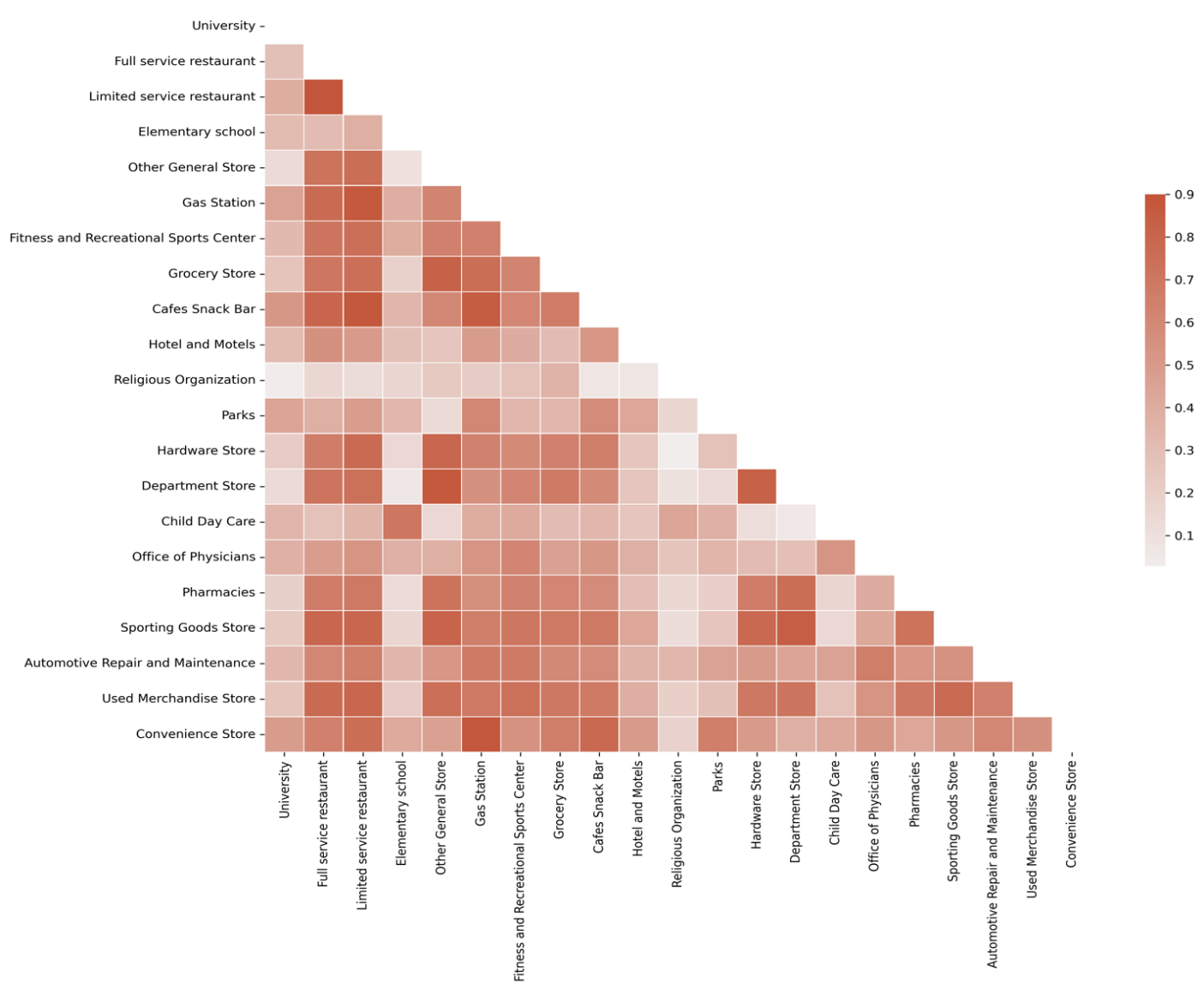


Supplementary Figure 2: The average correlations between all the visits metrics across 50 states. The color scales represent the magnitude of correlation coefficient, the deeper the color, the higher the correlation coefficient.

All the Pearson correlation coefficients are greater than 0, since all types of visits are more or less affected by lockdown and reopen policy. In addition, there are a few pairs of visits with a correlation above 0·8, which indicates that a feature selection step is necessary.

PCA is a technique to map a higher dimensional data to a lower dimension, and the variance in the lower dimension should explain most of the variations for the full data space. The principal component (PC) is a linear combination of all the features $X$, which can be expressed as:

$$PC=\boldsymbol{a}^{T}\boldsymbol{X}$$

And the covariance matrix $\boldsymbol{\Sigma}$ of PC is:

$$Var\left( \boldsymbol{a}^{T}\boldsymbol{X} \right)= \boldsymbol{a}^{T}\boldsymbol{\Sigma}\boldsymbol{a}$$

The goal of PCA is to preserve the original variance as much as possible, hence $Var\left( \boldsymbol{a}^{T}\boldsymbol{X} \right)$ should be maximized under the condition that $\boldsymbol{a}^{T}\boldsymbol{a}=\mathbf{1}$. Using the Lagrange’s multiplier, we could generate each PC as follow:

$$\mathcal{L=}\boldsymbol{a}^{T}\boldsymbol{\Sigma}\boldsymbol{a}- \lambda\left( \boldsymbol{a}^{T}\boldsymbol{a}-1 \right)$$

$$\frac{\partial\mathcal{L}}{\partial a}= 2\boldsymbol{\Sigma}\boldsymbol{a}- \mathbf{2}\lambda\boldsymbol{a}=0$$

$$\boldsymbol{\Sigma}\boldsymbol{a} = \lambda\boldsymbol{a}$$

Hence $\boldsymbol{\lambda}$ and $\boldsymbol{a}$ are the eigenvalues and eigenvectors of covariance matrix $\boldsymbol{\Sigma}$. Therefore, consider a dataset with $p$ variables, the $i\mathrm{th}$ principal component and its variance are:

$$PC_{i}=\boldsymbol{a}_{i}^{T}\boldsymbol{X}= \boldsymbol{a}_{1i}\boldsymbol{x}_{1}+ \boldsymbol{a}_{2i}\boldsymbol{x}_{2}+\ldots+ \boldsymbol{a}_{pi}\boldsymbol{x}_{p}$$

$$Var\left( PC_{i} \right)= \lambda_{i}$$

Here $\boldsymbol{a}_{i}$ are the desired weights assign to each feature. The total amount of variance explained by first $i$ components is:

$$\frac{\lambda_{1}+ \lambda_{2}+\ldots+ \lambda_{i}}{\lambda_{1}+ \lambda_{2}+\ldots+ \lambda_{p}}$$

The variance explained by the first five PCs are each state is shown in Appendix Figure 3 below, where x-axis is the percentage of variance explained and the red dashed line represents 80%.

**
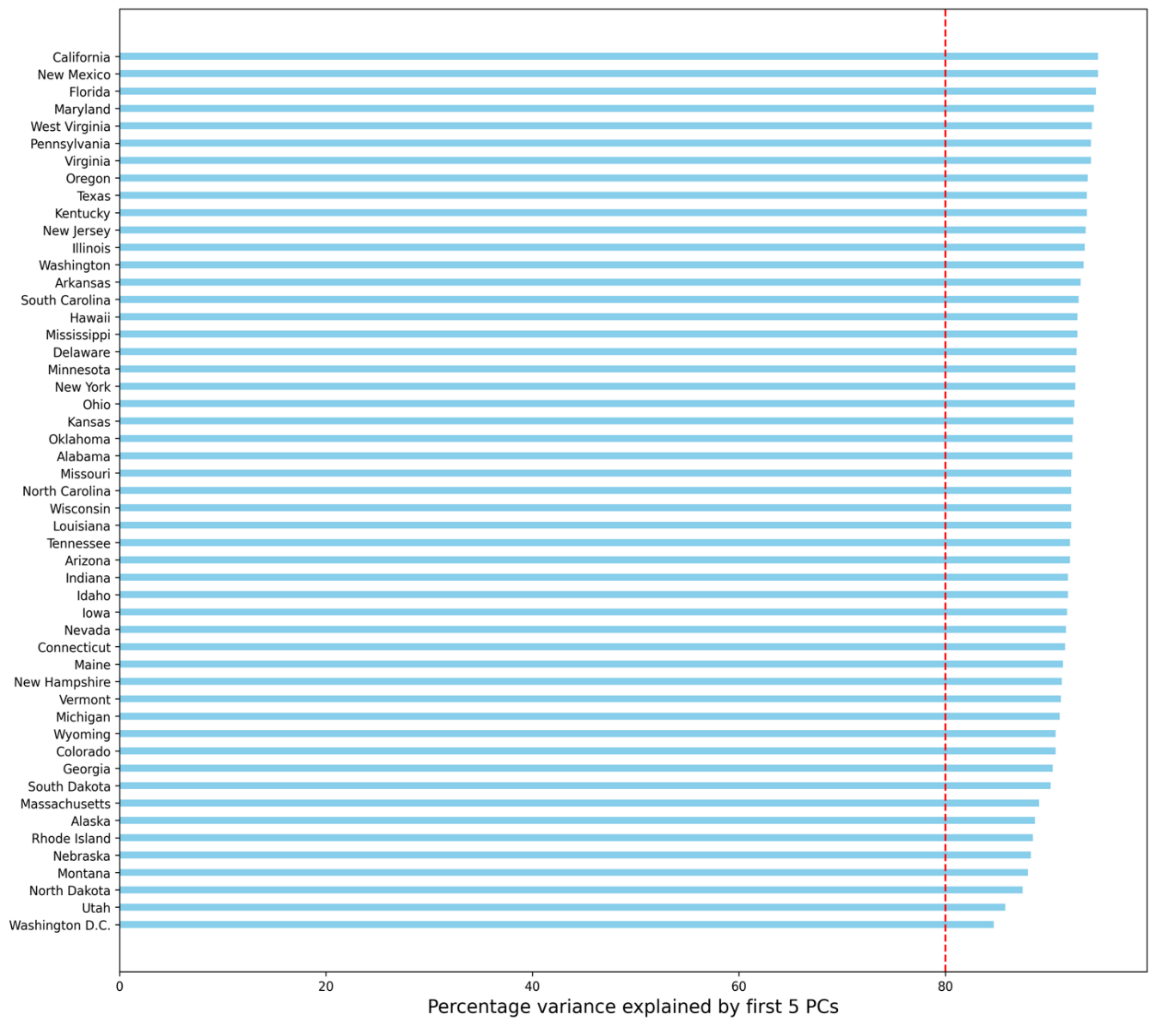
**

Supplementary Figure 3: Percentage variance explained by the first 5 PCs for all the states. The red dot vertical line represents 80% variance explained.

## **1.3 COVID-19 symptoms survey data**

Human behavior, while evasive to quantify, is thought to play a dominant role in the outbreak patterns observed for COVID-19. Various public survey efforts exist to generate behavioral indicators that can be used to better infer transmission dynamics, and these have been illustrated to improve predictive accuracy compared to those without them.^19^ In this work we utilize data from the COVID-19 symptoms survey. The survey is conducted through Facebook’s platform in collaboration with the Delphi group.^20^ From this survey we use “the estimated percentage of people who know someone in their community having Covid-like symptoms”. The timeseries data is provided at a daily resolution and smoothed using a 7-day moving averages to generate our input variable.

## **1.4 Climate data**

Some evidence points to climate and seasonality as potential factors associated with COVID-19 transmission,^21,22^ although its role remains unclear.^23^ To account for the possible impact of climate and seasonality on transmission risk, we include daily population-weighted hydrometeorological data, sourced from the JHU COVID-19_Unified-Dataset GitHub repository.^24^ The full set of variables we consider are near-surface air temperature (°C), and the total precipitation (mm/day).

## **1.5 Demographic data**

COVID-19 is known to have a disproportionate impact across demographic groups, specifically age and race.^25^ For this reason, we included total population and population percentage over 65 years of age as two separate static variables in our model. The population data were collected from the American Community Survey of the US Census Bureau.^26^ We use the 2019 Single Year of Age and Sex Population Estimates dataset to calculate the percentage of the population over 65 years old for each state.

## **1.6 SARS-CoV-2 variant frequencies data**

Of particular interest in this study is the potential value of SARS-CoV-2 variant frequencies data in forecasting models to predict surges that may be driven by new variants in a more accurate and timely manner, or more generally, the impact new variants might have on COVID-19 transmission patterns. To address this research questions, we conduct a case study that utilizes COVID-19 genomic data downloaded directly from GISAID.^27^ From the available set of sequences, we calculate the proportion of each variant (theoretically in circulation) each day over the course of the pandemic.

Based on the sample collection date (October 7th, 2021), we select the United States data at the state level between April 1, 2021, to end of August 2021. First, we calculated the coverage rate as the total number of genomic samples over the number of confirmed cases for each state during the selected period. Then, we filtered out the states with less than 5% of coverage. We selected all the State with at least 5% of overall sampling coverage. As shown in Appendix Figure 4, all the states with color have sampling coverage higher than 5%, while the states in grey have coverage less than 5%.


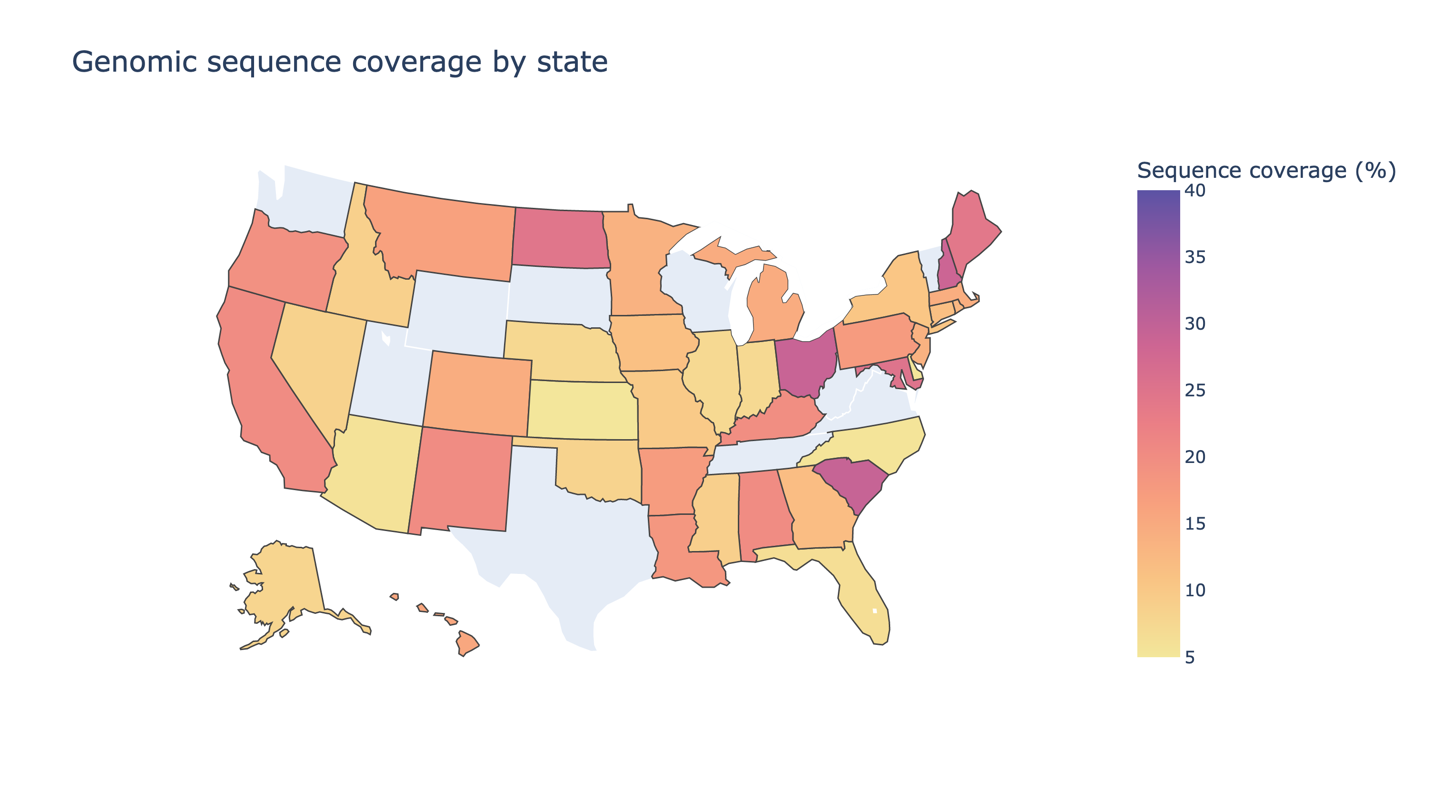


Supplementary Figure 4: Genomic sequence coverage by state between April 1st, 2021 and August 21st, 2021. The color scales represent the magnitude of the sequence coverage, the deeper the color, the higher the coverage.

For Delta wave, we generate time series of lineage proportion for the Delta, Gamma, and Alpha variants among all the virus lineages. Then, all the rest of the lineages are classified as others. The four proportion time series are smoothed with a 7-day moving average. We assume that this proportion of variants samples also apply to the proportion of variant cases within the confirmed cases. The genomic features are defined as:

$$g_{i,j}^{t}= {log(p}_{i,j}^{t}c_{j}^{t}+1) , i \in\{Delta, Gamma, Alpha and Others\}$$

Where $g_{i,j}^{t}$ is estimated logarithm of confirmed cases for variant group $i$ for state $j$ at time $t$, $p_{i,j}^{t}$ is the proportion of variant group $i$ for state $j$ at time $t$ and $c_{j}^{t}$ is the confirmed cases for state $j$ at time $t$.

We calculate the collection to submission time for all collected genomic data by October 7th, 2021. The distribution of CST is shown in Appendix Figure 5, with a median CST of 24 days. In our case study, we apply a scenario analysis to test the value of the genomic data, we assume all the genomic data will be available with a CST of 7 days. By doing this, we want to provide a proof of concept that the timely genomic data (7-days lag) is valuable for short-time COVID-19 forecasting.


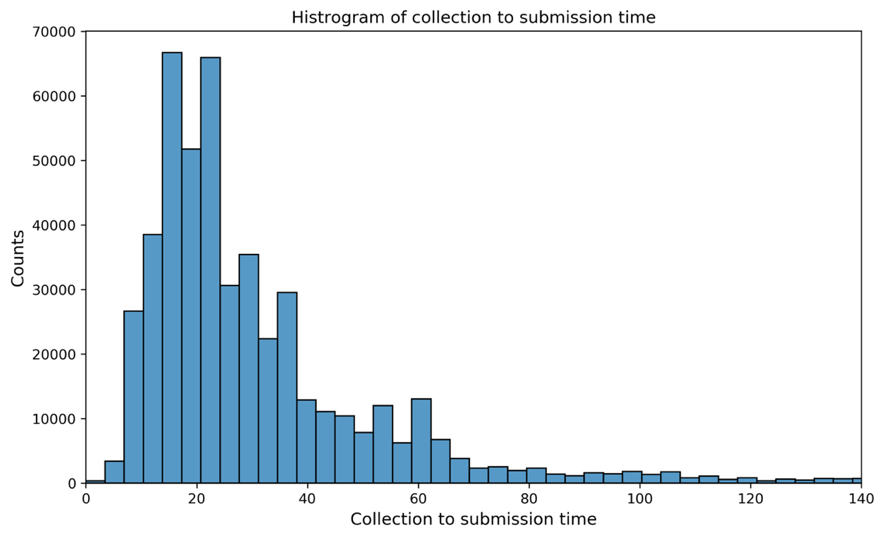


Supplementary Figure 5: Histogram of collection to submission time as October 7th, 2021

The proportion of selected variant for Delta wave is shown in Supplementary Figure 6. Each dot represents the raw proportion on a given day and each solid line represents the trend smoothed by Generalized Additive Model (GAM). The deeper the color, the earlier the Delta variant became dominant in that state. The timeseries illustrate the quick rise of the Delta variant from May 15 and July 15, at which time it because the dominant lineage across the U.S., soon converging to 100%. However, by the end of August, the Delta variant was already being replaced by the Omicron variant (which occurred outside this study period).


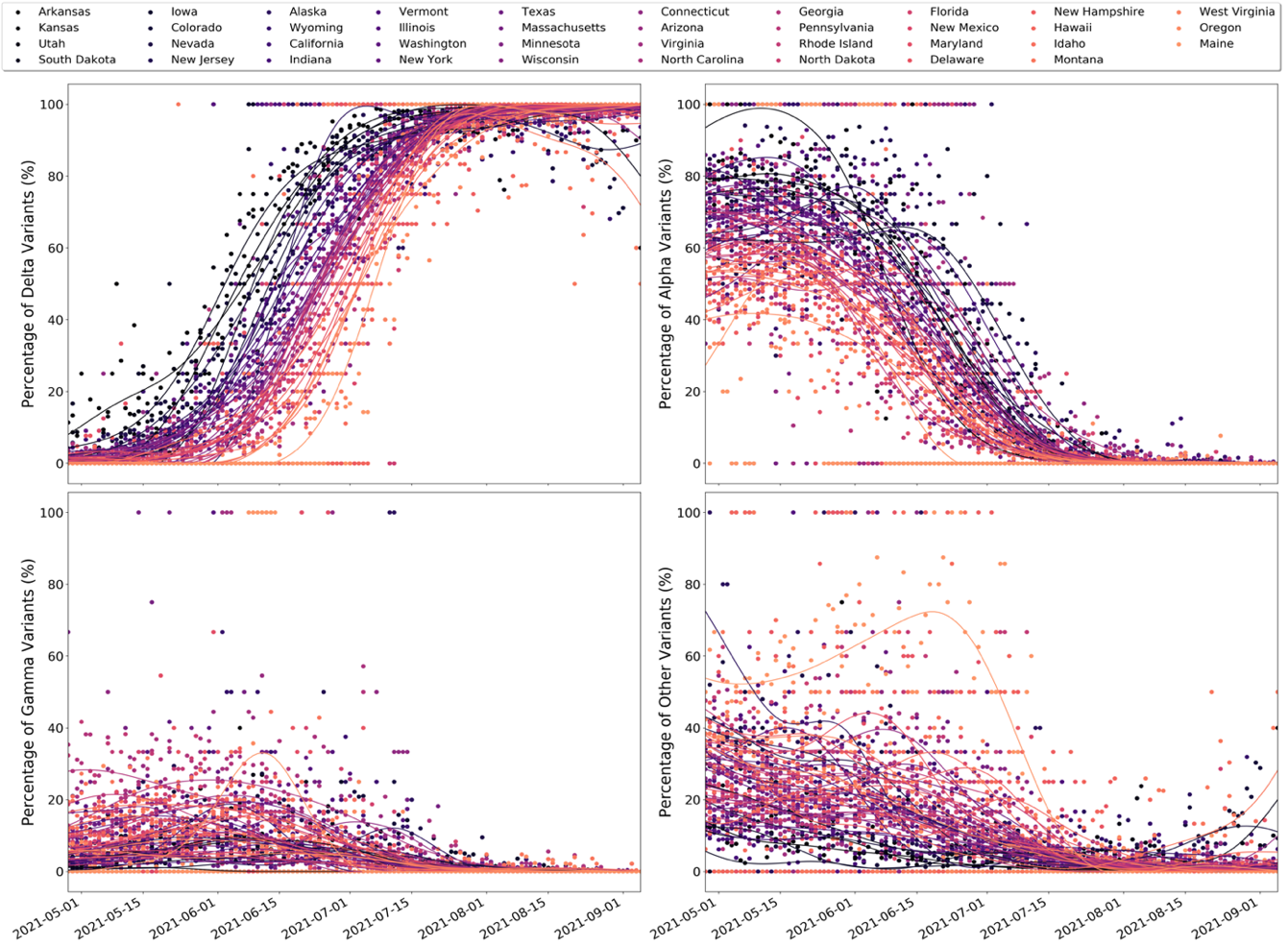


Supplementary Figure 6: Time varying proportion of selected variant for 39 states in the U.S between 2021-05-01 to 2021-09-01. Colors represent data for different state. The dots represent the raw variant frequencies data while the plotted lines are smoothed with a GAM.

We apply the same data preprocessing for Omicron waves and the proportions of selected variants for Omicron wave are shown in Appendix Figure 7 and 8.


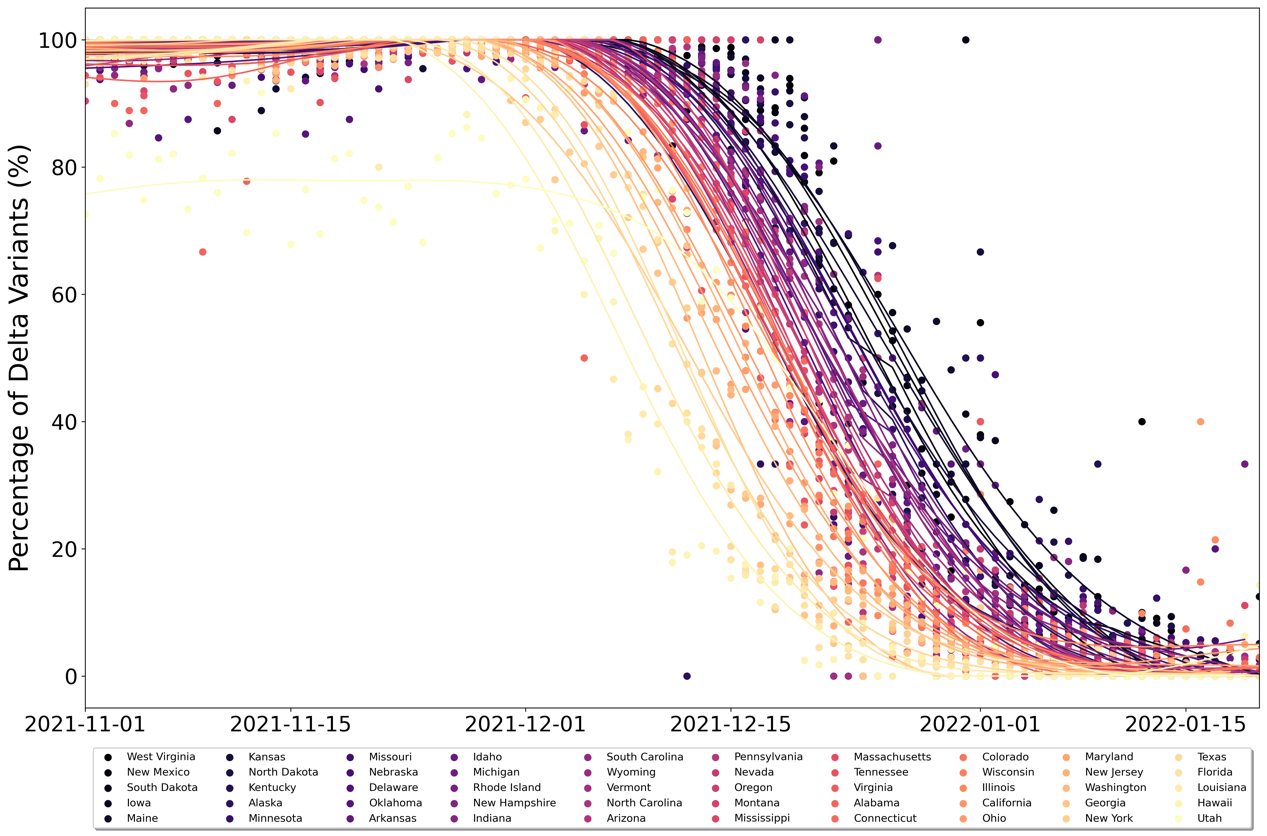


Supplementary Figure 7: Time varying proportion of Delta variant for states in the U.S between 2021-11-01 and 2022-01-20. Colors represent data for different state. The dots represent the raw variant frequencies data while the plotted lines are smoothed with a GAM.


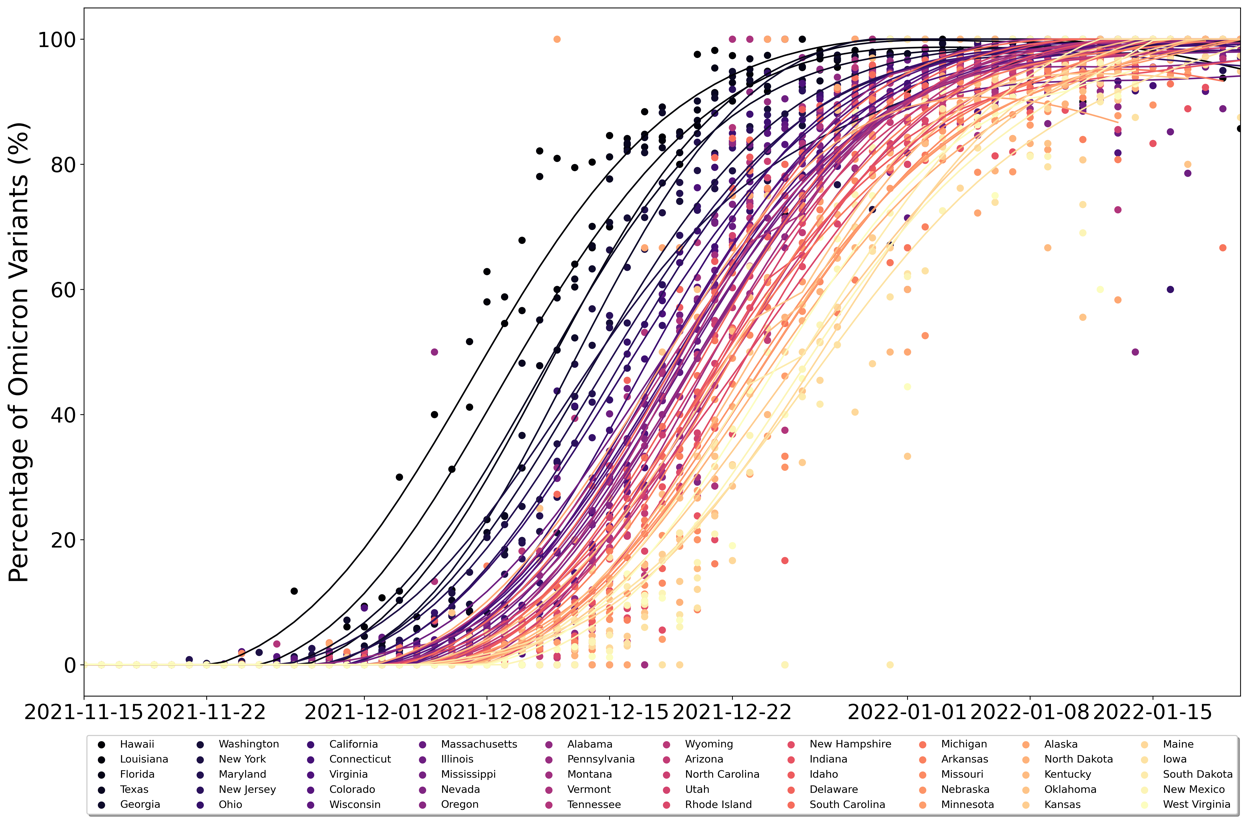


Supplementary Figure 8: Time varying proportion of Omicron variant for states in the U.S between 2021-11-01 and 2022-01-20. Colors represent data for different state. The dots represent the raw variant frequencies data while the plotted lines are smoothed with a GAM.

# **2. Supplementary method**

## **2.1 An example implementation of the multi-stage LSTM model**

The multi-stage frame train the framework to predict reported cases/deaths and other time series input for the next 7 days. At the initial stage, the model uses the most recent data as input, then at the later stage, the model adapts previous prediction as input to make further predictions. For example, if we wish to predict the number of new COVID-19 cases each week for the next 2 weeks using as input three time-varying features from the weeks prior: incident cases, cases growth rate, and MR, the model stages are set up as follows:

Stage 1: We use observed data from the last three weeks, specifically day $(t-21)$ to day $t$ as the length of the sequence of the input data for both the main and feature model. The outputs from the main model are incident cases in the week following, $t+1$to $t+7$ (total predicted cases for one-week ahead). The outputs from the feature model are the case growth rate and MR for the same one-week ahead window, $t+1$to $t+7$. Together, the forecasted values from the main and feature model provide the required input for the following stage (stage 2), which aims to predict incident cases for two weeks ahead.

Stage 2: The stage 2 outputs from the main model are incident cases from $t+8$ to $t+14$, which are then converted to our second week predicted cases, while the outputs from the feature model are cases growth rate and MR from $t+8$to $t+14$. To generate these 2-week ahead outputs, we require a timeseries from $t-14$(observed) to $t+7$ (unknown at time t), again, as the model requires a three-week length of the sequence of the input data. The stage one output enables the extension of each of the time series from the current time t to $t+7$. We use a combination of the observed data ($t-14$to day t) and outputs from stage 1 (time t to $t+7)$to generate the required timeseries as input for stage 2. This same process is repeated to generate inputs for each following stage. For any combination of target variables, both the main and feature models are trained on the same dataset and applied simultaneously to generate predictions.

## **2.2 Formulations of LSTM model**

Both the main and feature models introduced above have the same network structure, which is built by one layer of long-short term memory (LSTM) network ^28^ and two layers of Multilayer perceptron (MLP). LSTM is a special kind of recurrent neural network, which is capable of learning long-term dependencies. The key idea behind LSTM is the cell state ($C_{t}$) controlled by three gates named input gate ($i_{t}$), output gate ($o_{t}$) and forget gate ($f_{t}$). The cell state keeps the information that passes along the sequence, and those three gates help filter information in the cell state at each time point. The output $h_{t}$ from the LSTM layer will serve as input to pass through MLP layers. Then the finial predictions are the outputs from MLP layers. We apply the dropouts at the MLP layers to include randomness for the predictions.

The LSTM layer can be formalized as follow:

$$f_{t} = sigmoid(W_{f}[h_{t-1}, x_{t}] + b_{f})$$

$$i_{t} = sigmoid(W_{i}[h_{t-1}, x_{t}] + b_{i})$$

$$\hat{C_{t}}= tanh(W_{c}[h_{t-1}, x_{t}] + b_{c})$$

$$C_{t} = f_{t}\times C_{t-1} + i_{t}\times\hat{C}_{t}$$

$$o_{t} = sigmoid(W_{0}[h_{t-1}, x_{t}] + b_{o})$$

$$h_{t} = o_{t}\times tanh(C_{t})$$

where $x_{t}$ and $h_{t}$ denote, respectively, input data and hidden state at time $t$. $\hat{C}_{t}$ denotes a candidate new cell state that could be added to the cell state. $W_{j}$ and $b_{j}$ represent weights and bias terms at each gate or cell state $j$. The prediction function for the entire network model can be formulated as:

$$y(t+1, ..., t+7) = W_{2}(Relu(W_{1}(h_{t}) + b_{1})) + b_{2}$$

where $y(t+1, ..., t+7)$ are the predicted cases from $t+1$ to $t+7$, $W_{1}$,$W_{2}$,$b_{1}$ , and $b_{2}$ are weights and biases for the fully connected neural network, $Relu$ is the activation function:

$$Relu(x) = max(0,x)$$

## **2.3 Model parameterization**

The models are implemented using Python 3·8 with open sources packages such as PyTorch, Pandas and NumPy. In initial setting of the multi-stage Neural Network model, different size of hidden layers in LSTM were explored for model training and testing. The sensitivity analysis showed subtle differences of performance between different settings. Hence, we set size of hidden layers as constant for both main model and features model. The full dataset is randomly divided into two sets, 70% for training and 30% for testing, early stop will apply, if the testing error no longer improving. We use smoothed L1 loss as the model loss function, the formulation is described below:

$$l\left( x,y \right)= \left\{ \begin{aligned} \frac{0.5(x-y)^{2}}{\beta}, if \left| x-y \right|< \beta\\ \left| x-y \right|+0.5\beta, otherwise \end{aligned} \right.$$

The model performances are most sensitive to the training periods; we test different combinations of training periods for the main model and the features model. Finally, we use 1 layer of LSTM layer connected with two dense connected layers. For the main model, we set the hidden layer size as 256, and for the features model, we set the hidden layer size as 328.

## **2.4 Model evaluation metrics**

The formulations for AE, PAE, and WIS ^29^ are defined below. $F$ is the model prediction function;$u$, $m$ and $l$ are the upper bound, the median, and the lower bound of the predictions, respectively, and $y$ is the ground truth.

$$AE=|m-y|$$

$$PAE= \frac{|m-y|}{y} \times100\%$$

For each $\left( 1- \alpha\right)\times100\%$ prediction interval, the WIS is defined as:

$${IS}_{\alpha}\left( F,y \right)=\left( u-l \right)+ \frac{2}{\alpha}\times\left( l-y \right) \times1\left( y<l \right)+ \frac{2}{\alpha}\times\left( y-u \right) \times1(y>u)$$

$$W{IS}_{\alpha\{0:K\}}\left( F,y \right)=\frac{1}{K+1/2}\times(w_{0}\times\left| y-m \right|+ \sum_{k=1}^{K} \{w_{k} \times{IS}_{\alpha_{k}}\left( F,y \right)\})$$

where $w_{k}= \frac{\alpha_{k}}{2}$, in this paper we choose $K=3$ with $\alpha_{1}=0\cdot05$, $\alpha_{2}=0\cdot2$ and $\alpha_{3}=0\cdot5$.

## **2.5 Model Selection**

All the results in the main document are based on our best performance model. We apply sensitivity analysis for four alternative models under two time period separately. From August 2020 to Feb 2021, the epidemiological data do not contain vaccination data, while since February 2021 to August 2021, we add the vaccination data to the epidemiological data category. Static variables are automatically included for all the models. The category assignment for each variable can be found in main document table 1.

There are two steps for the model selection: 1) Determine the optimal training periods for each candidate model. 2) Under the optimal training periods, find the best performing model. For this part of the analysis, we evaluated our models based on point predictions.

For each of the four models, we test different training periods for the main model ($t_{m}$) and the features model ($t_{f}$). Each prediction is evaluated by PAE and AE. In this study, we weighted them equally in model selection. The rule for selecting the best training period is described below:

$$L\left( t_{m},t_{f} \right)= \sum_{i} (\frac{{AE}_{t_{m},t_{f},i}}{max\{{AE}_{i}\}} + \frac{{PAE}_{t_{m},t_{f},i}}{max\{{PAE}_{i}\}}) , i=1, 2, 3, 4$$

Where $L\left( t_{m},t_{f} \right)$ is an aggregated loss function of hyperparameters $t_{m}$ and $t_{f}$; ${AE}_{t_{m},t_{f},i}$ and ${PAE}_{t_{m},t_{f},i}$ are average absolute error and average percentage absolute error at forecasting window $i;$ $max\{{AE}_{i}\}$ and $max\{{PAE}_{i}\}$ are maximum errors corresponding to all the combinations of ${(t}_{m},t_{f})$. We select $t_{m}$ and $t_{f}$ that minimize the $L\left( t_{m},t_{f} \right)$ (Supplementary Table 2).

Supplementary Table 2: Summary of training periods selection.

| **Input features** | **Time Period** | $\boldsymbol{t}_{\boldsymbol{m}}$ | $\boldsymbol{t}_{\boldsymbol{f}}$ |
| --- | --- | --- | --- |
| Epidemiological data | August 2020 to February 2021 | 60 | 45 |
| Epidemiological data | February 2021 to August 2021 | 30 | 30 |
| Epidemiological, and mobility data | August 2020 to February 2021 | 60 | 75 |
| Epidemiological, and mobility data | February 2021 to August 2021 | 40 | 40 |
| Epidemiological, mobility, and survey data | August 2020 to February 2021 | 45 | 75 |
| Epidemiological, mobility, and survey data | February 2021 to August 2021 | 30 | 30 |
| Epidemiological, mobility, survey, and climate data | August 2020 to February 2021 | 60 | 45 |
| Epidemiological, mobility, survey, and climate data | February 2021 to August 2021 | 40 | 40 |

Once the training periods were determined, we further evaluated the performances between different inputs. The results of comparing these four models with the CDC ensemble model from August 2020 to August 2021 are shown in Appendix Figure 9 and 10 reveal the break down performance before and after February 2021. Both PAE and AE are used to evaluated performance, and the value for each bar in the plots is the average over all states and time.


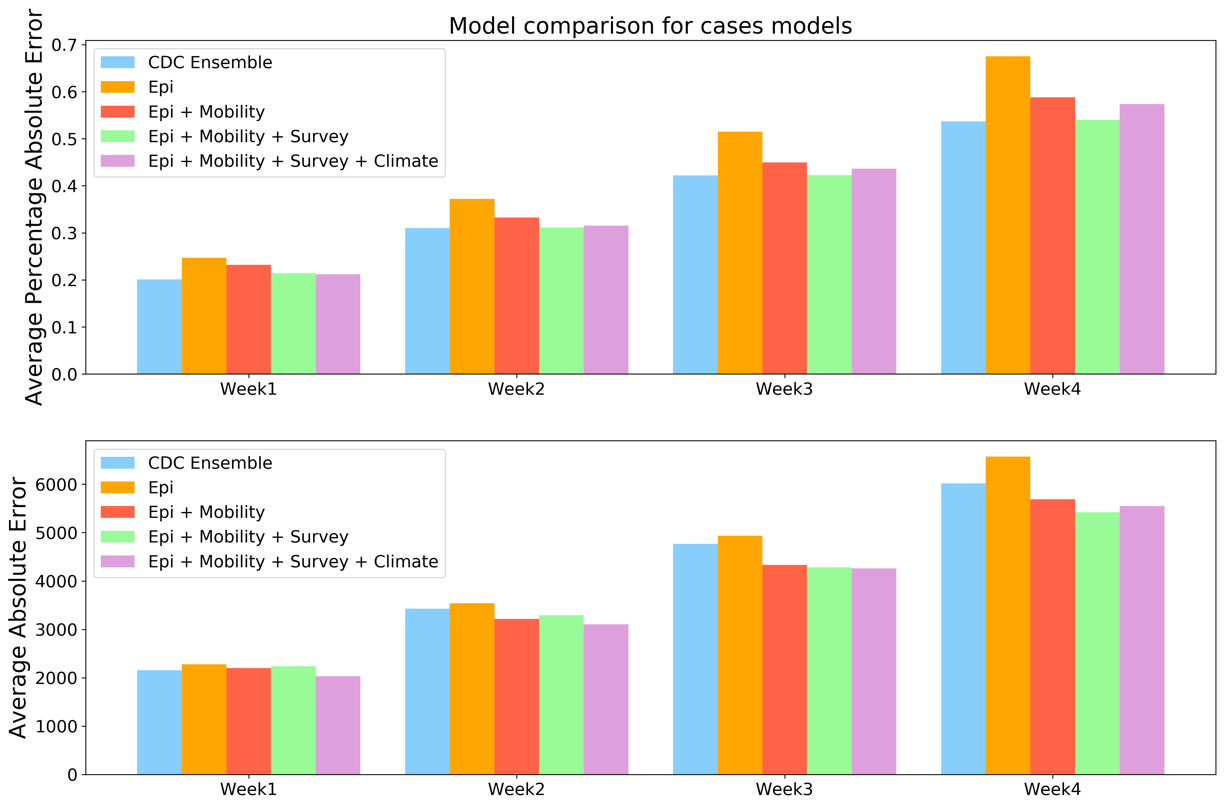


Supplementary Figure 9: Model comparison for cases models by the mean PAE and the mean AE for the period between August 2020 to September 2021. The y-axis represents the average absolute error for 1-4 weeks cases’ prediction results across the entire study period.


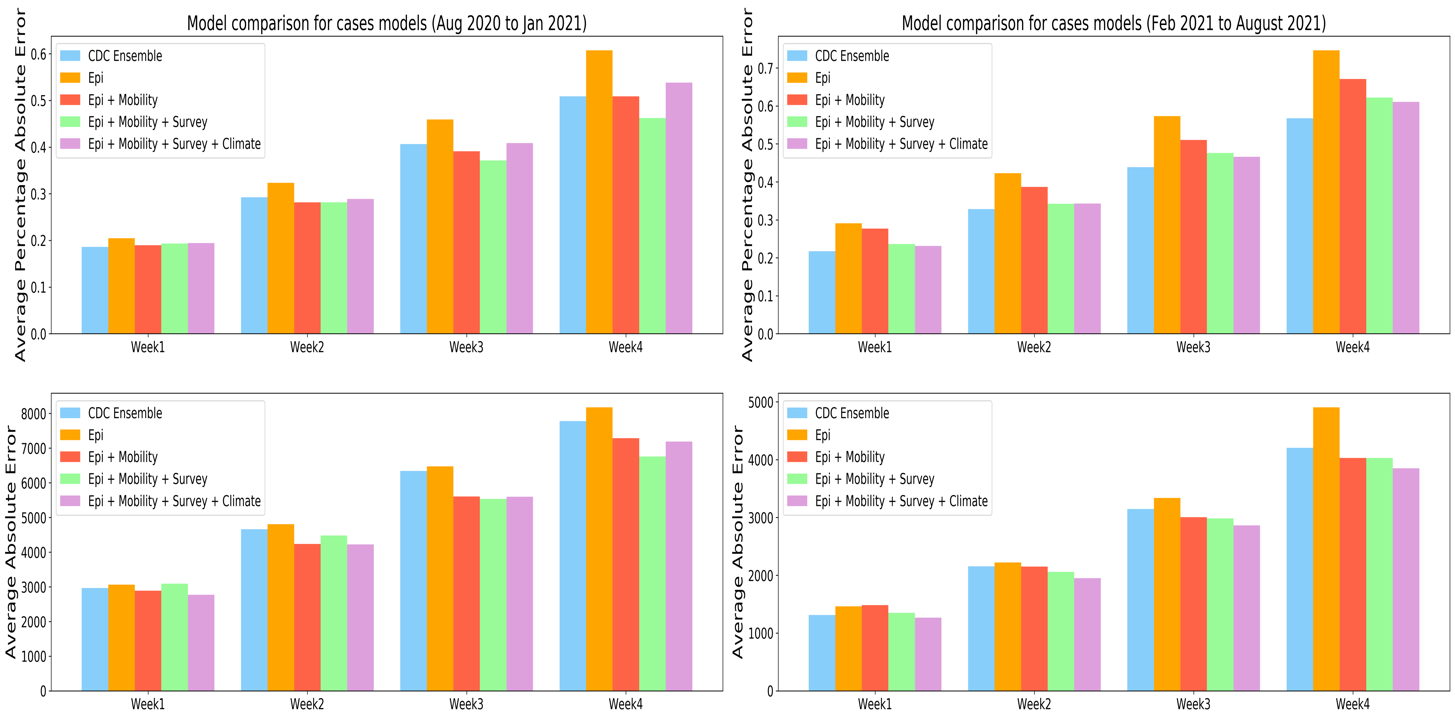


Supplementary Figure 10: Model comparison for cases models by the mean PAE and the mean AE during two periods: from August 2020 to February 2021, and from February 2021 to August 2021. The y-axis represents the average absolute error for 1-4 weeks cases’ prediction results across selected period.

## **2.6 Feature Importance**

Integrated Gradient (IG) is an interpretability technique for deep neural networks, which calculates the gradient of the model’s prediction with respect to its input features ^30^. IG requires no adjustment to the model architecture and could provide visualization of feature importance to the model predictions. The formulation of IG for a specific feature $i$ is as follow:

$${IG}_{i}\left( x \right)∷=(x_{i} - x_{i}^{'})\int_{\alpha=0}^{1} \frac{\partial F(x^{'}+ \alpha(x- x^{'}))}{\partial x_{i}}$$

where $x$ are all the input data, $x^{'}$ represent baseline values, $\alpha$ is an interpolation constant and $F$ is the function of deep network.

We apply the IG for the model with genomic surveillance data to highlight the contribution of adding variant-specific time series. For each feature, we sum the feature contribution over the input sequence length to get the total contribution. The contribution of each feature varies across time and space. Hence, we present the results when introducing a new variant, switching of dominant variant, and a new variant becoming dominant.


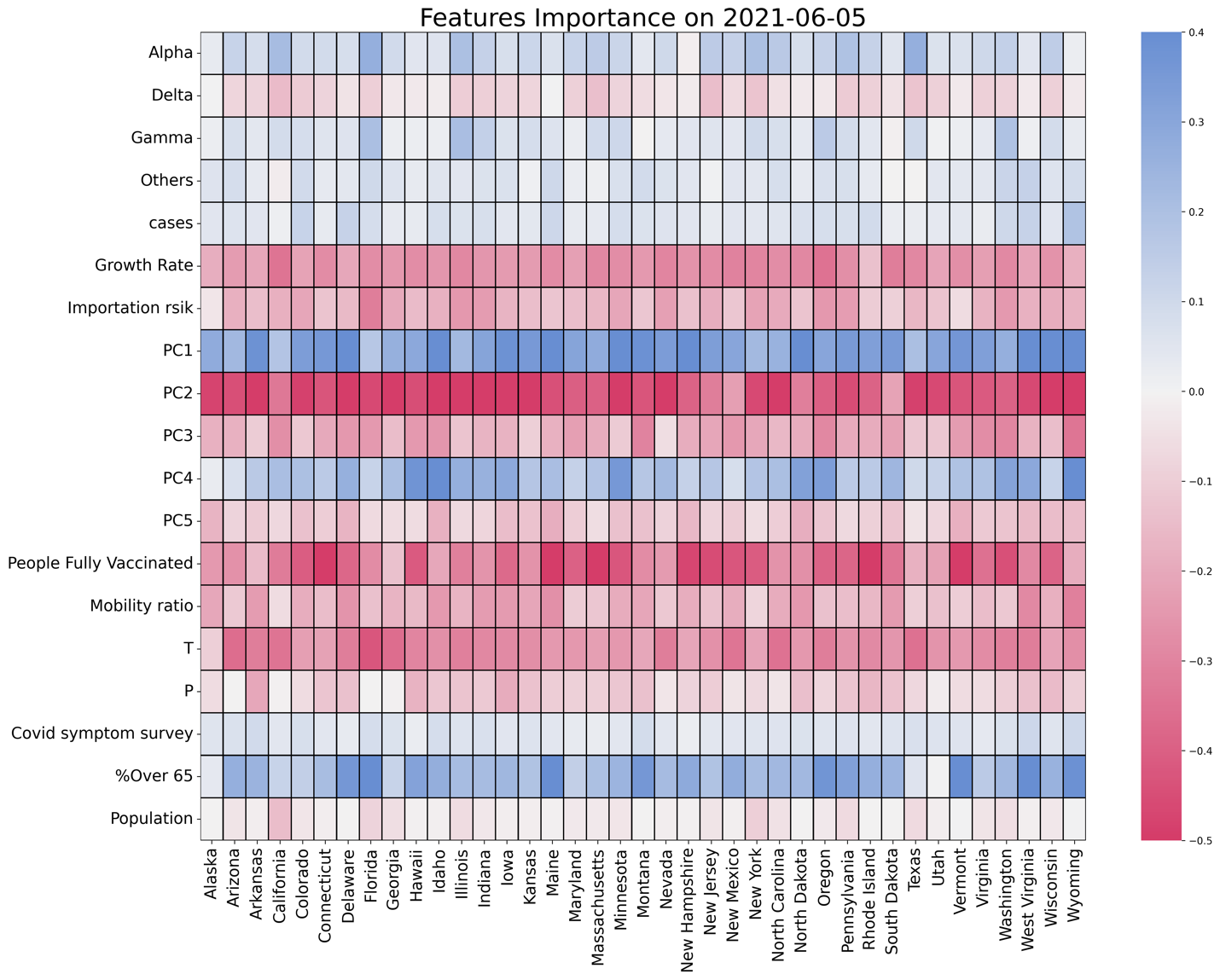


Supplementary Figure 11: Feature Importance for the Multi-stage LSTM model on 2021-06-05 when the average frequency for the Delta variant is 14% (initial introduction of the Delta variant). The Y axis represents features, and the X axis indicates corresponding states. The color bar represents the contribution of each feature. The blue color indicates a positive impact, and the red indicates a negative impact.


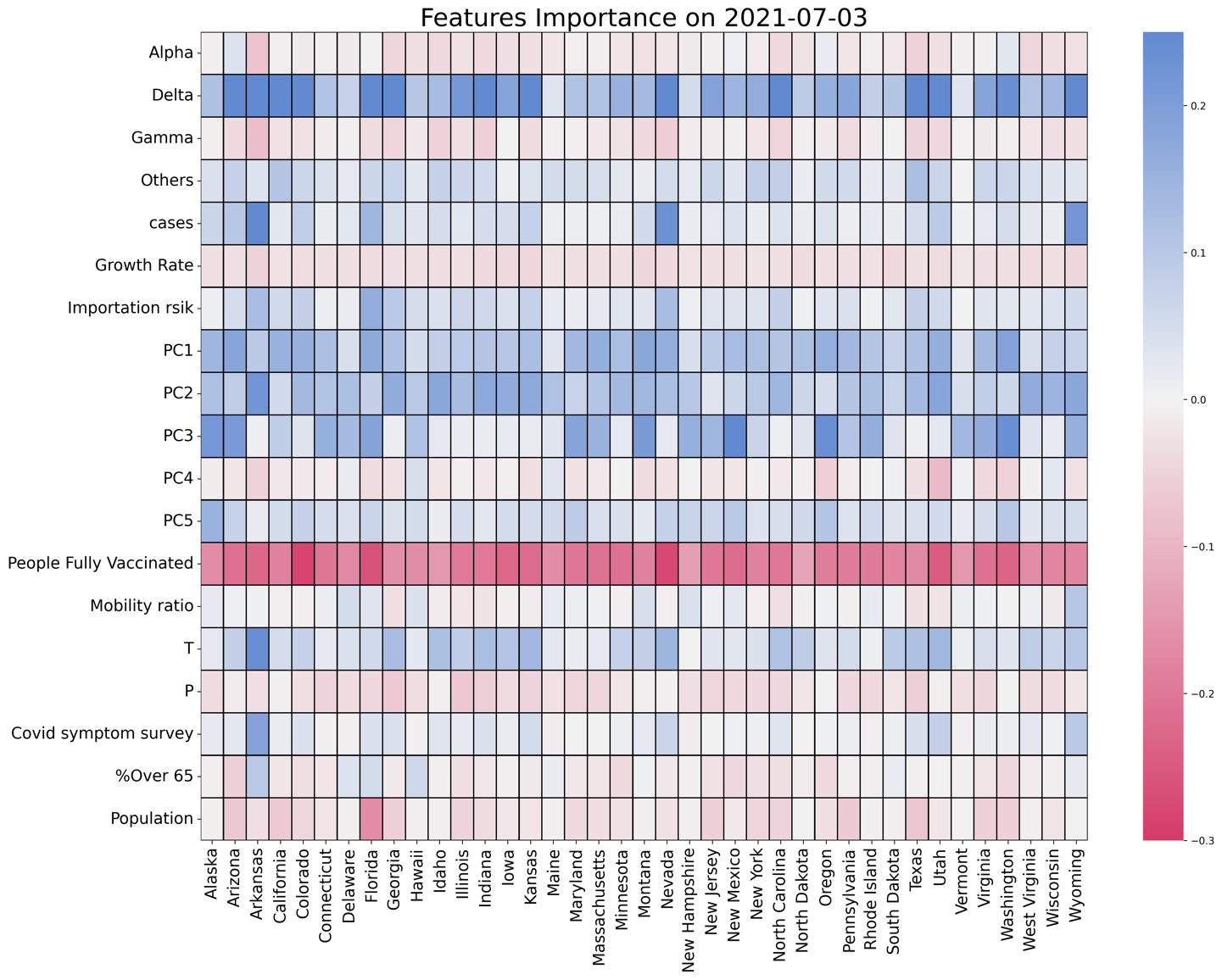


Supplementary Figure 12: Feature Importance for the Multi-stage LSTM model on 2021-07-03 when the average frequency for the Delta variant is 66% (period transition from Alpha to Delta becoming dominant). The Y axis represents features, and the X axis indicates corresponding states. The color bar represents the contribution of each feature. The blue color indicates a positive impact, and the red indicates a negative impact.


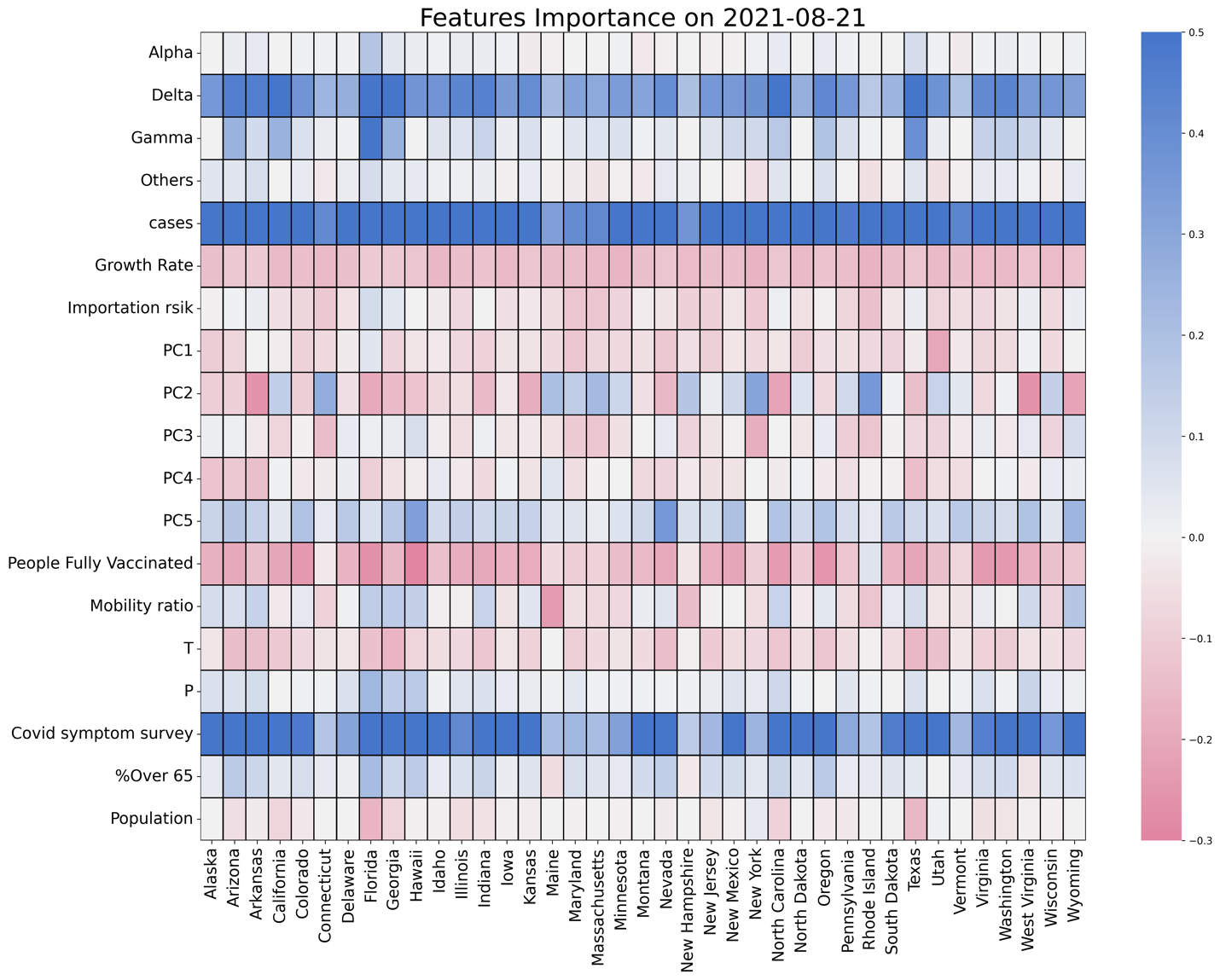


Supplementary Figure 13: Feature Importance for the Multi-stage LSTM model on 2021-08-21 when the average frequency for the Delta variant is 97% (the Delta variant reaching maximum proportion). The Y axis represents features, and the X axis indicates corresponding states. The color bar represents the contribution of each feature. The blue color indicates a positive impact, and the red indicates a negative impact.


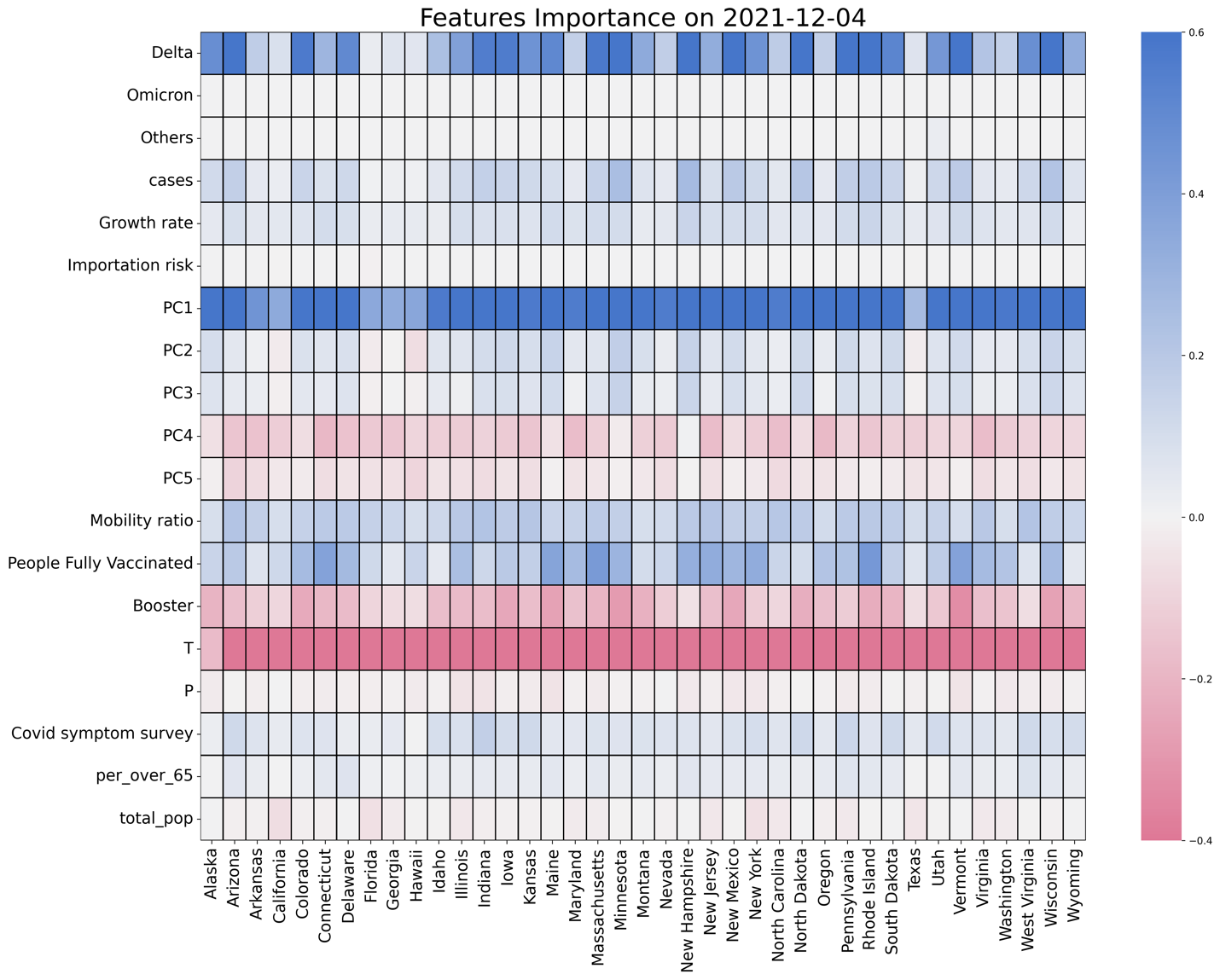


Supplementary Figure 14: Feature Importance for the Multi-stage LSTM model on 2021-12-04 when the average frequency for the Omicron variant is 1% (initial introduction of the Omicron variant). The Y axis represents features, and the X axis indicates corresponding states. The color bar represents the contribution of each feature. The blue color indicates a positive impact, and the red indicates a negative impact.


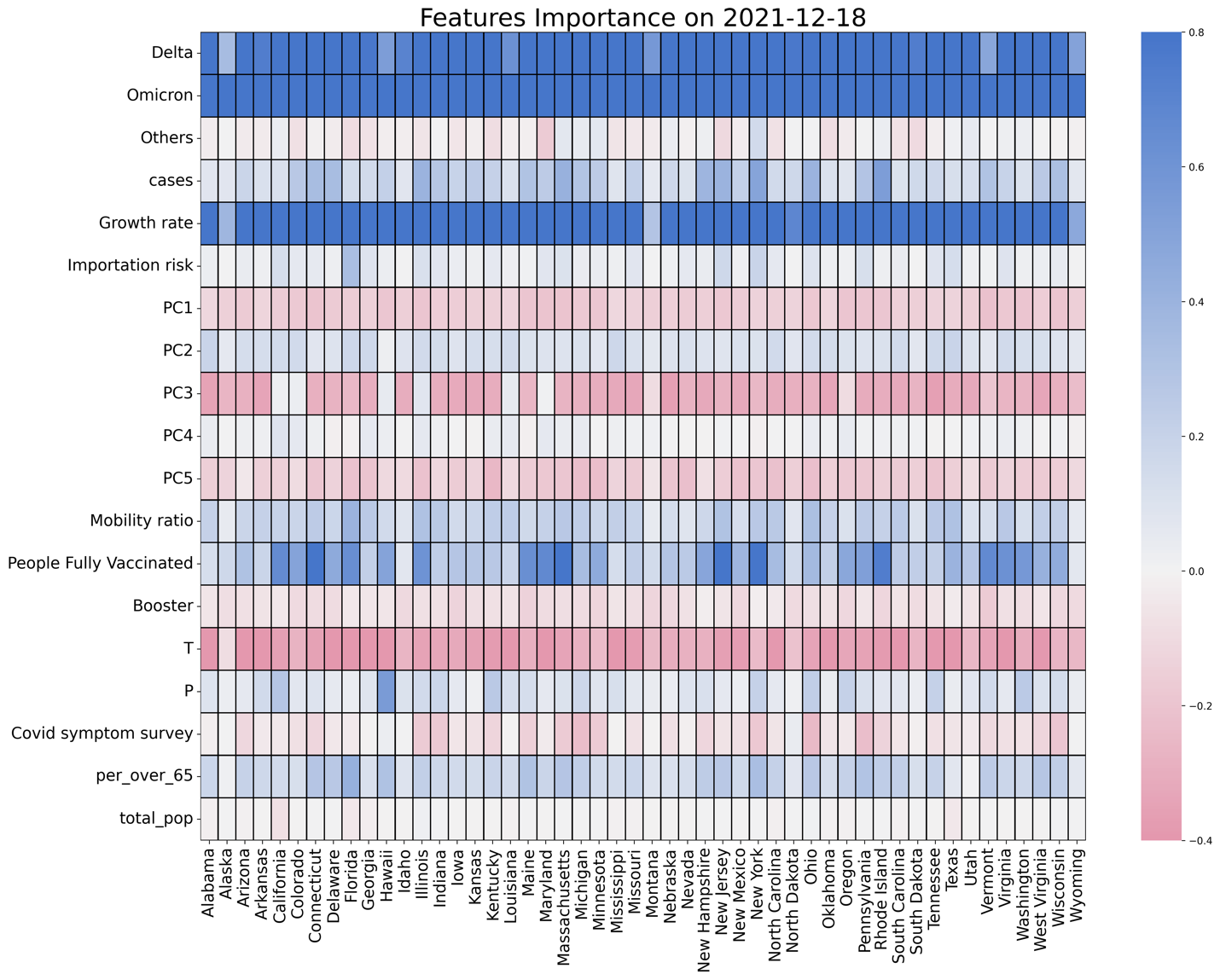


Supplementary Figure 15: Feature Importance for the Multi-stage LSTM model on 2021-12-18 when the average frequency for the Omicron variant is 41% (period of transition from Delta to Omicron becoming dominant). The Y axis represents features, and the X axis indicates corresponding states. The color bar represents the contribution of each feature. The blue color indicates a positive impact, and the red indicates a negative impact.


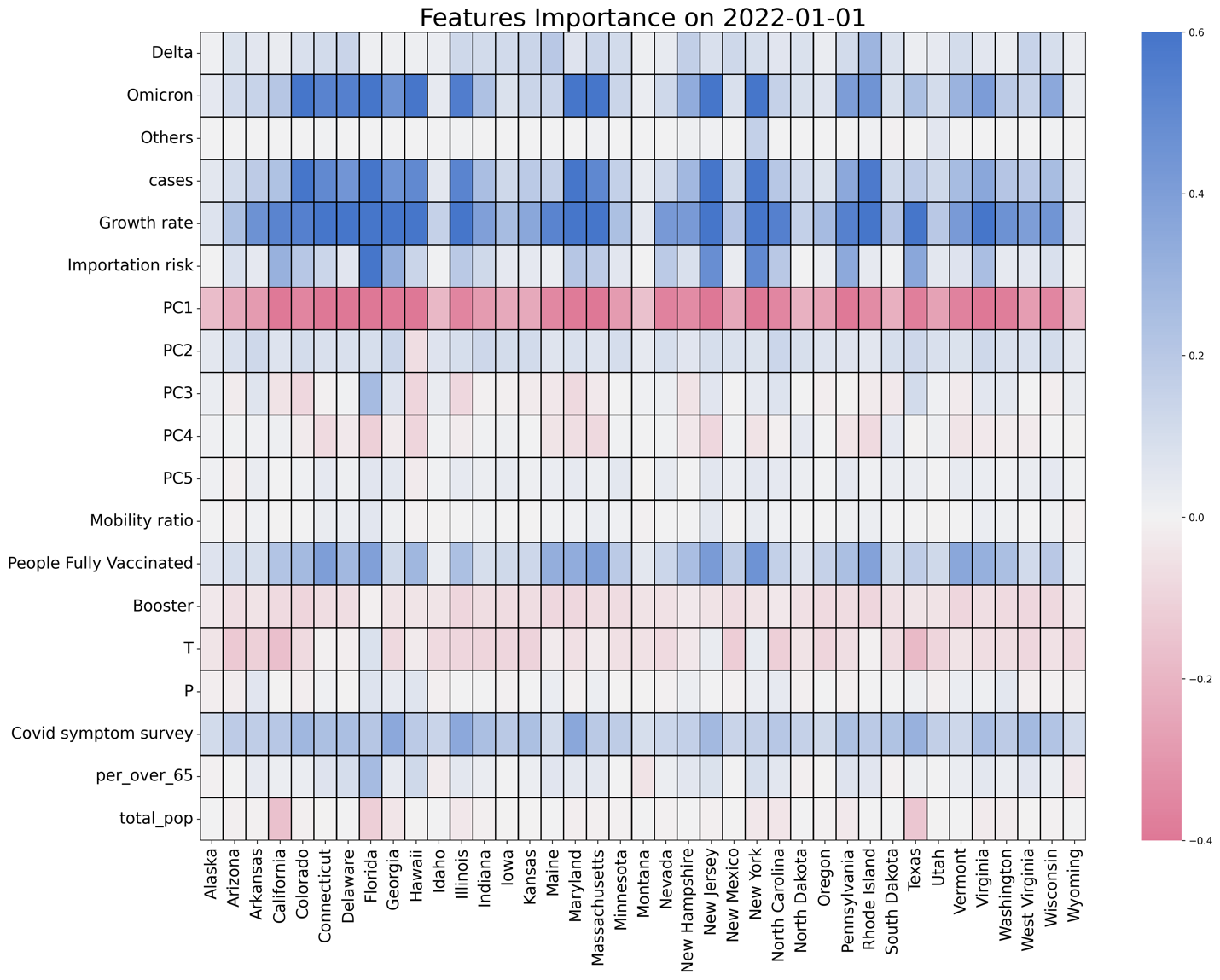


Supplementary Figure 16: Feature Importance for the Multi-stage LSTM model on 2022-01-01 when the average frequency for the Omicron variant is 85% (the Omicron variant reaching maximum proportion). The Y axis represents features, and the X axis indicates corresponding states. The color bar represents the contribution of each feature. The blue color indicates a positive impact, and the red indicates a negative impact.

# **3. Supplementary results**

## **3.1 Model performance across time by AE and WIS**

Appendix Figure 17, and 18 illustrate the relative performance of the LSTM against the CDC ensemble model for each of the 52-week periods evaluated, for 1 to 4 week forecast windows, based on AE and WIS respectively. Each pair of bar plots represents PAE distribution for all the states at a given week, where the green bar represents the error distribution for the multi-stage LSTM model, and the yellow bar represents the error distribution for the CDC ensemble model. The red curve represents the weekly reported cases at the national level.

**
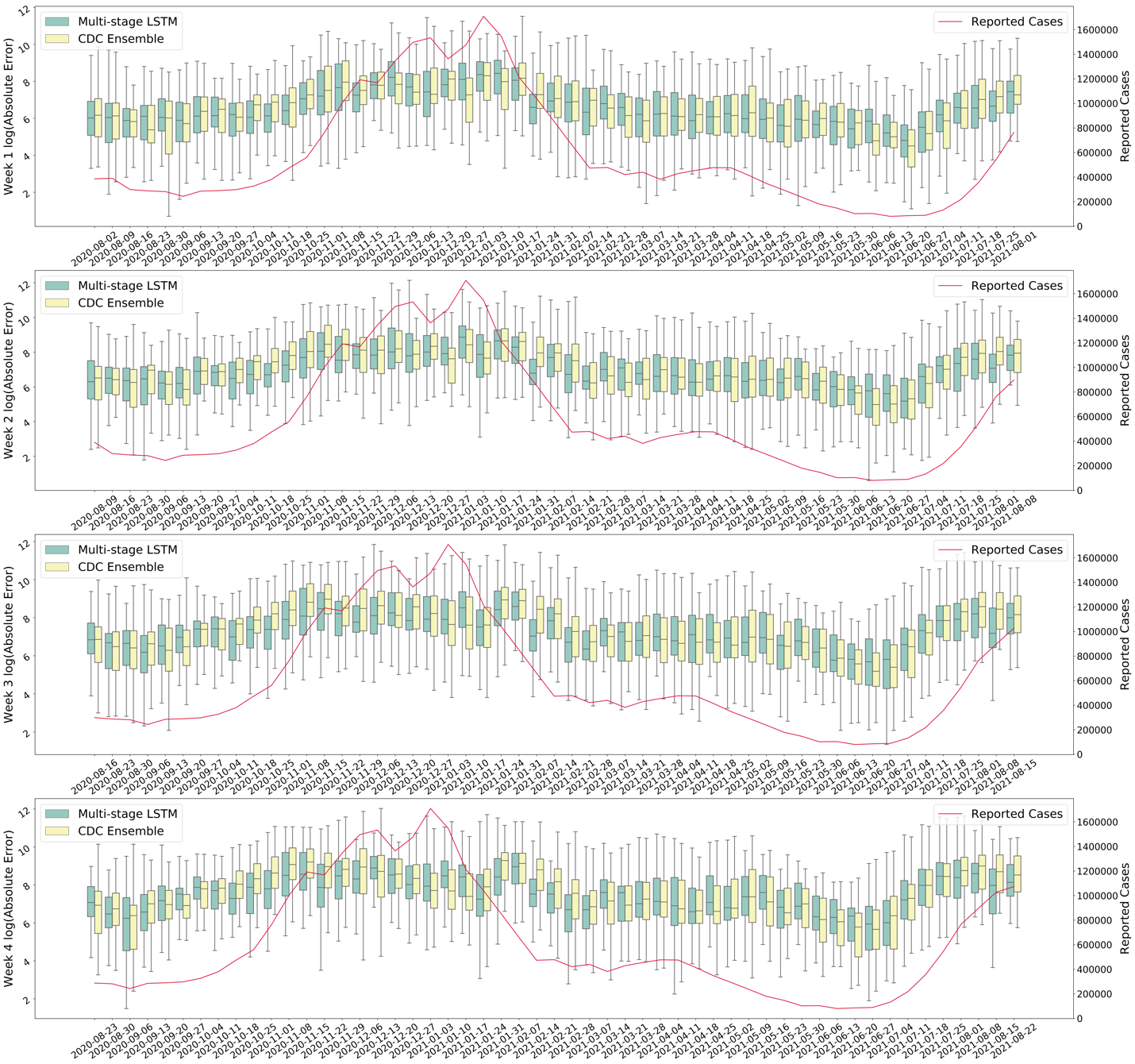
**

Supplementary Figure 17: Comparison of model performance (AE) between the multi-stage LSTM Model and the CDC ensemble model. For better visualize the results, we normalize AE by taking the log. The left y-axis represents the log AE for 1-4 weeks cases’ prediction results and right y-axis represents national level reported cases.

**
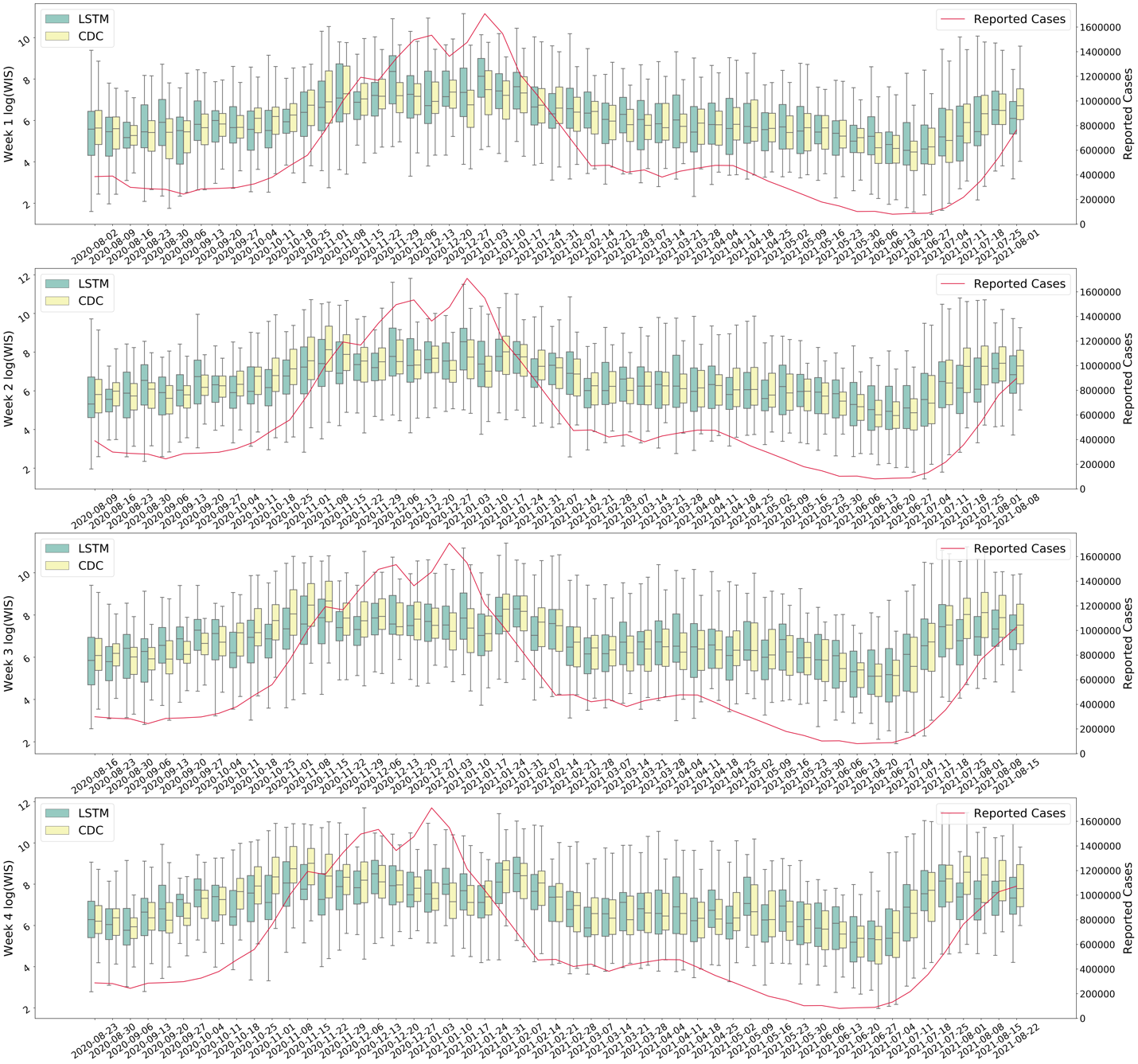
**

Supplementary Figure 18: Comparison of model performance (WIS) between the multi-stage LSTM Model and the CDC ensemble model. For better visualize the results, we normalize WIS by taking the log. The y-axis represents the log WIS for 1-4 weeks deaths’ prediction results.

## **3.2 Model performance across states by AE and WIS**

The Pearson’s correlation coefficient for raw WIS and the state-level population is 0·96. Thus, we normalized the WIS by population to remove population bias. For visualization purpose we convert AE into logarithm scale. The color scales represent the magnitude of each error metric; the scales are fixed as (1,10) and (6,11) for WIS and normalized log(AE), respectively. Hence, the deeper the color, the larger the error for the state.

**
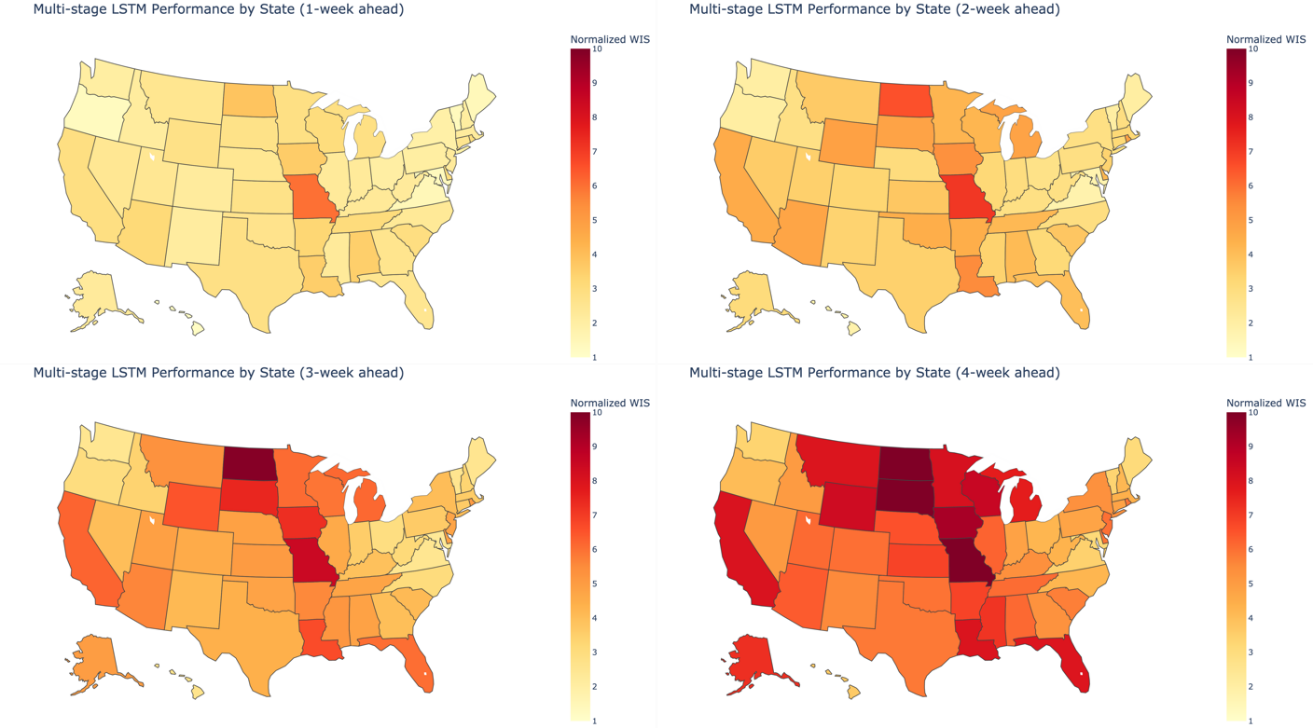
**

Supplementary Figure 19: Average model performance at the state level by normalized WIS. The color scales represent the magnitude of the error metric; the scales of normalized WIS are fixed in 1–10 range. The deeper color corresponds to larger error.

**
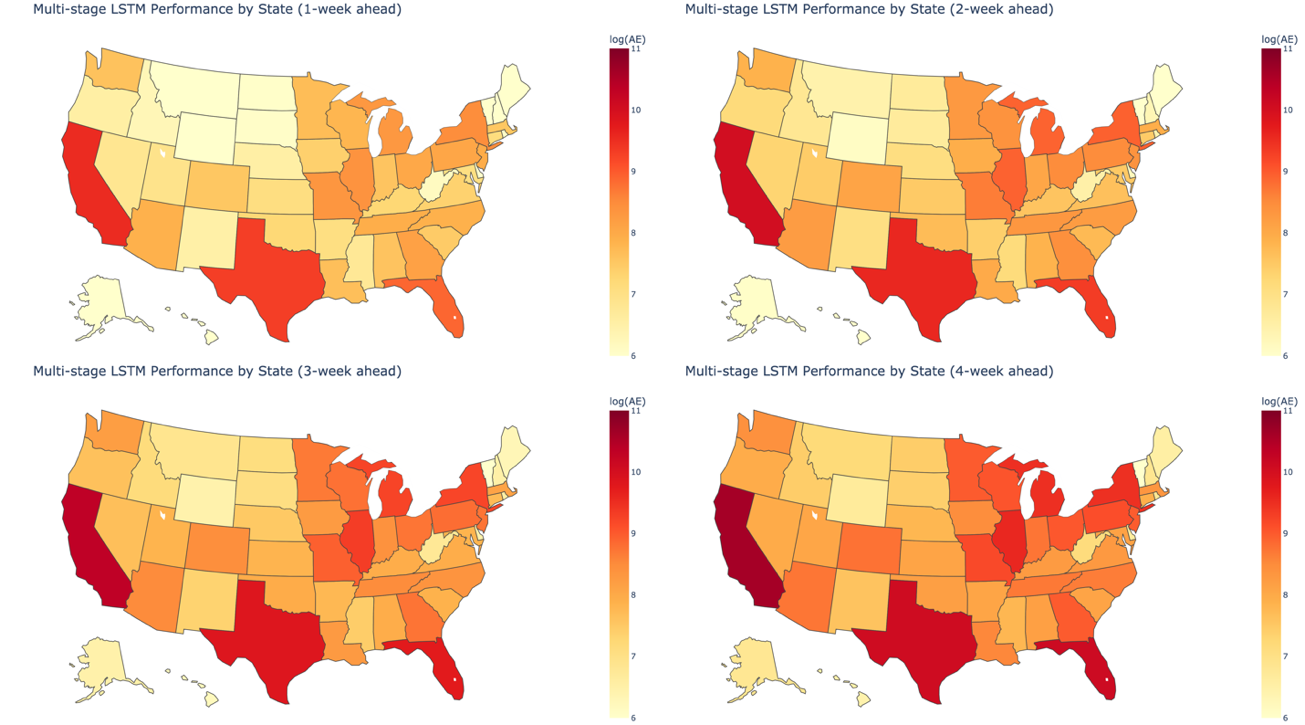
**

Supplementary Figure 20: Average model performance at the state level by logarithm of AE. The color scales represent the magnitude of the error metric; the scales of logarithm AE are fixed in 6–11 range. The deeper color corresponds to larger error.

## **3.3 Reported Cases Trend by Region**

To better understand the presence of spatial patterns in model performance as illustrated in Figures 3, we generate and compare state-level confirmed case trends between each region.

We first group states into each HHS region as shown in Appendix Figure 21 below. Most of the midwestern states are included in region 5, region 7, and region 8.


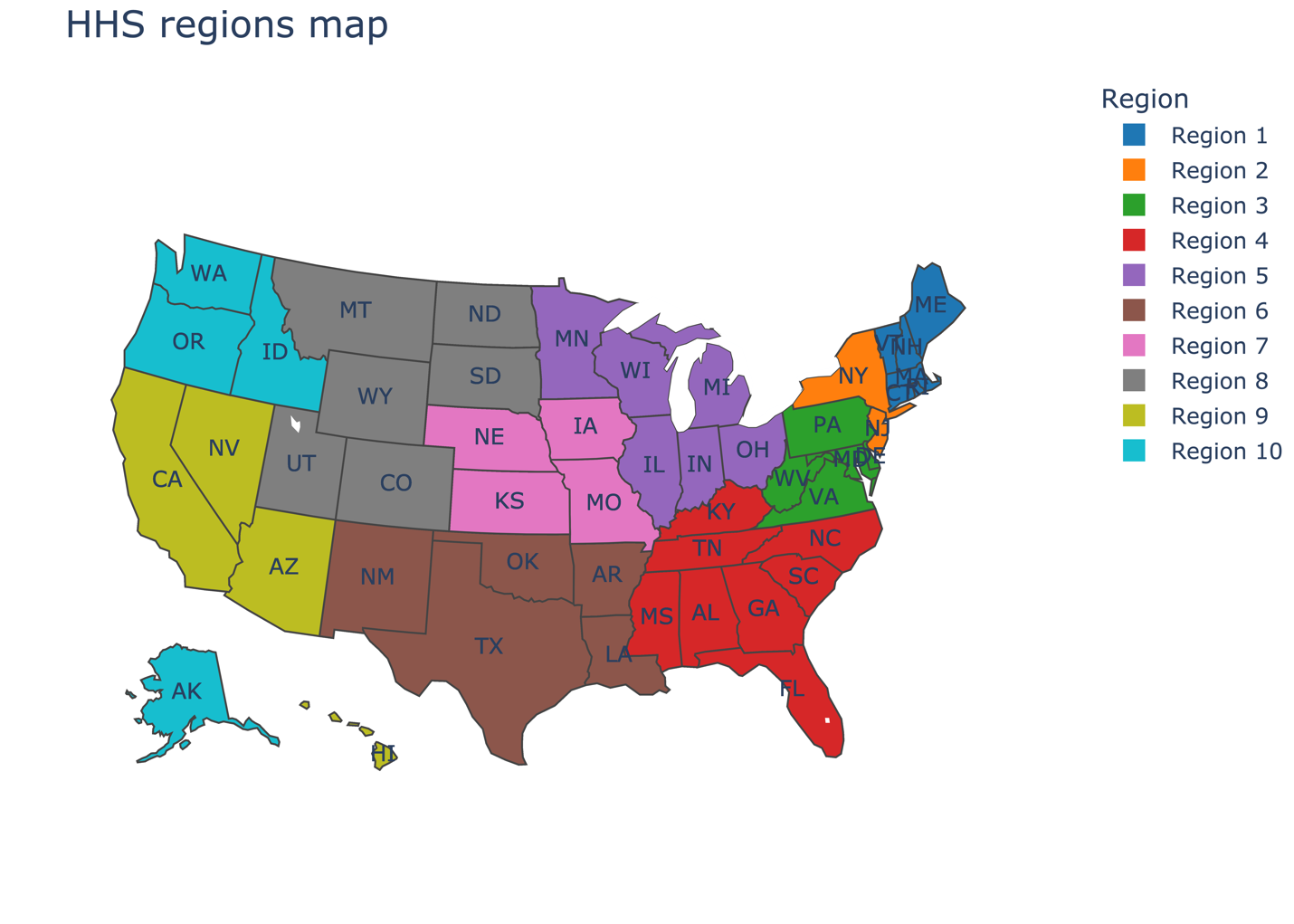


Supplementary Figure 21: HHS regions map.

We define a trend variable $T_{i}^{t}$ for each state $i$ at each week $t$ using the weekly confirmed cases from August 2020 to August 2021 for each state as follows:

$$T_{i}^{t}= \frac{C_{i}^{t}}{max(C_{i}^{1}, \ldots, C_{i}^{n})}$$

where $C_{i}^{t}$ is the number of confirmed cases for state $i$ at week $t$, and $max(C_{i}^{1}, \ldots, C_{i}^{n})$ is the maximum weekly confirmed cases over the selected weeks 1 to $n$, for state $i$. $T_{i}^{t}\in[0, 1]$ represents how reported cases for state $i$ at week $t$ compare to the highest reported cases over the entire period.

For each HHS region $j$, we calculate the average trendline $\bar{T}_{j}^{t}$ based on all state trends within the region:

$$\bar{T}_{j}^{t}=\frac{\sum_{i\in j} T_{i}^{t}}{N_{j}}$$

where $N_{j}$ is the number of states within HHS region $j$. This variable represents the normalized reported cases trend for each HHS region (i.e. when the most of states within a region show decreasing or increasing trend). The normalized $\bar{T}_{j}^{t}$ variable enables better comparison of trends across regions since $\bar{T}_{j}^{t}$ $\in[0, 1]$.The results of $\bar{T}_{j}^{t}$ are shown in Appendix Figure 22.


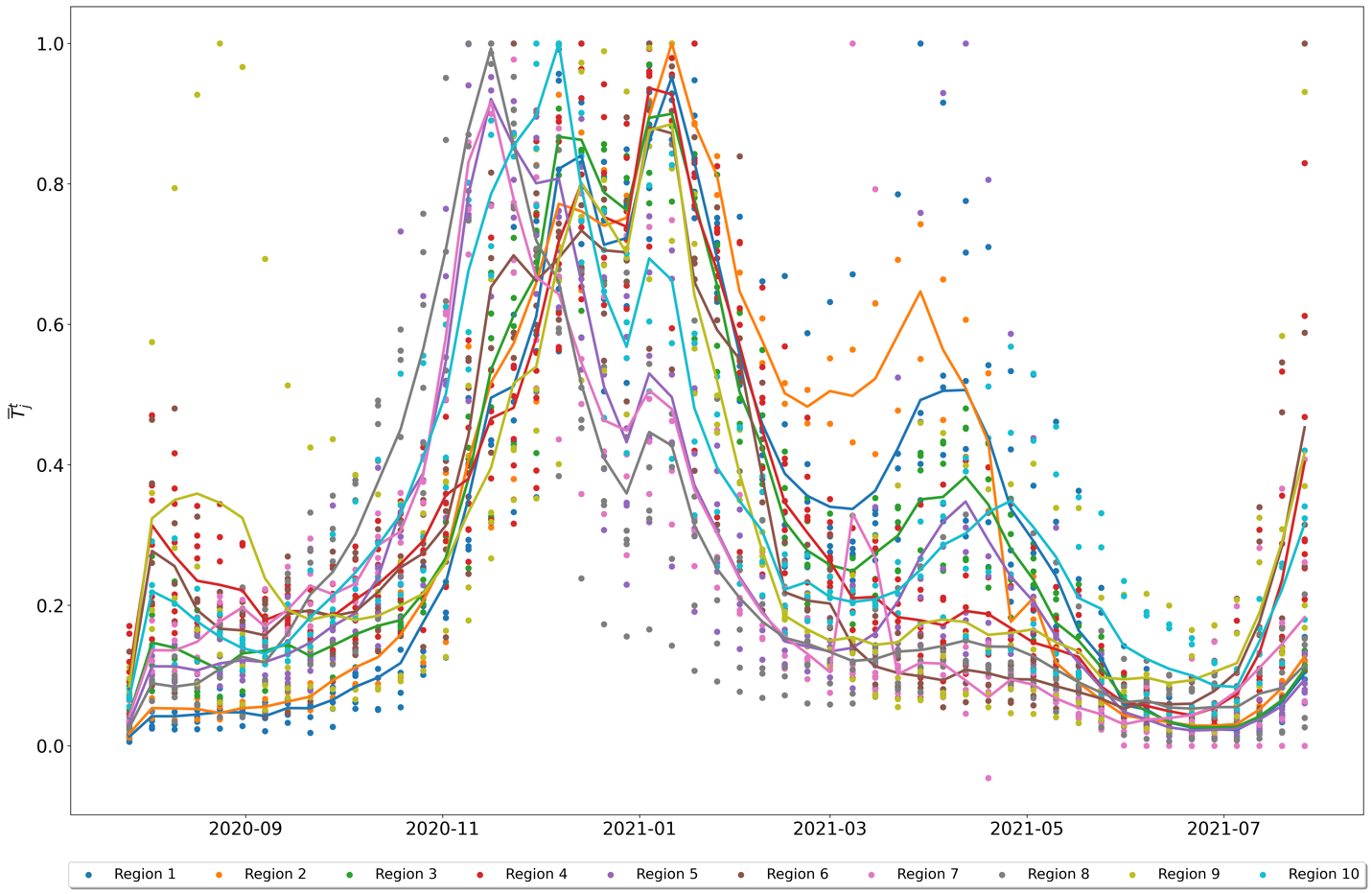


Supplementary Figure 22: Weekly confirmed cases trend $\bar{T}_{j}^{t}$ by HHS regions.

Most of the midwestern states are included in region 5, region 7, and region 8. This plot illustrates different transmission patterns for regions 5, 7 and 8, relative to the rest of the country between November 2020 and February 2021. Specifically, while most of regions have upward $\bar{T}_{j}^{t}$, the three regions mentioned above have decreasing trends. This result could be a possible explanation for why the forecasts for the midwestern states is less accurate relative to the rest of the country (as shown in Figure 3 and Appendix Figure 19).

## **3.4 An example of classification for different outbreak phases**


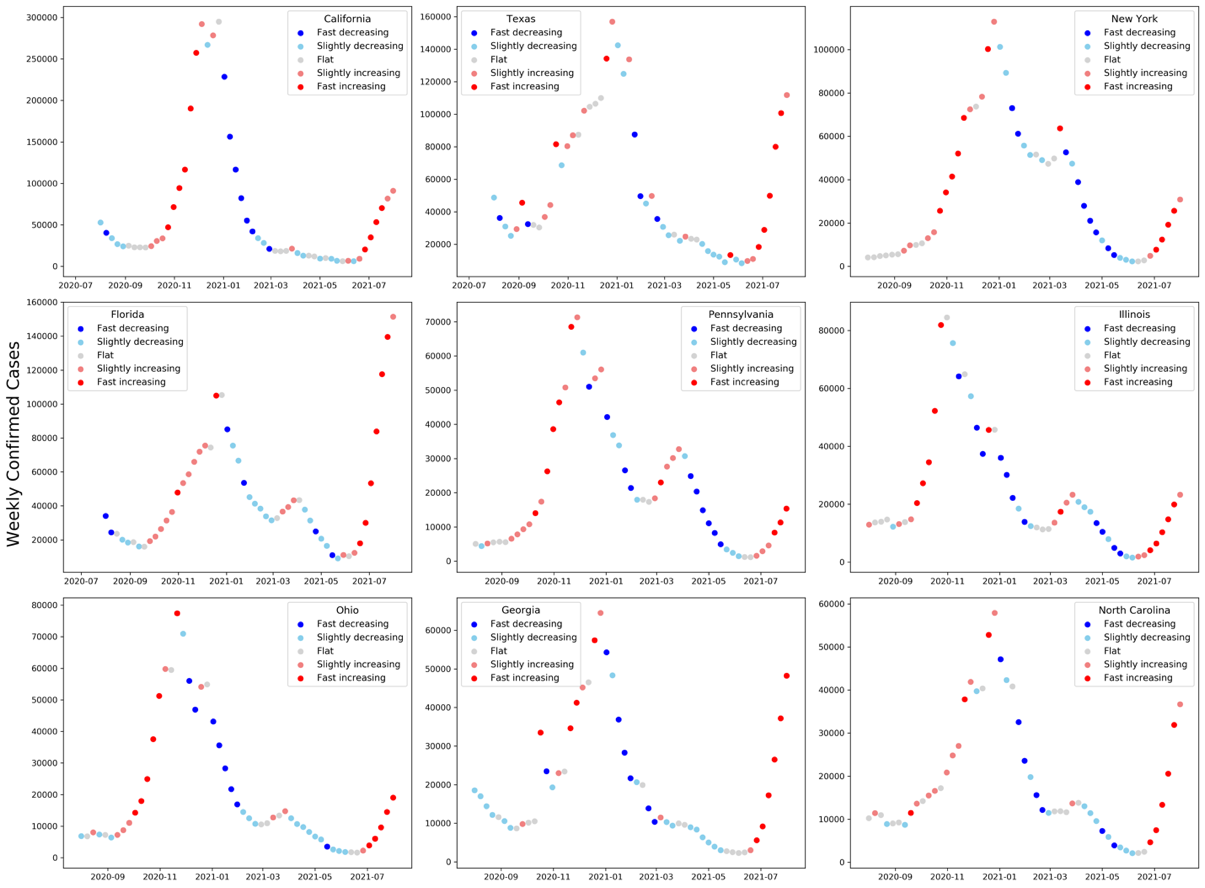


Supplementary Figure 23: Week group based on weekly growth rate for nine selected states. The y-axis represents the weekly reported cases and the color of the dots indicates the cluster group for the given week.

## **3.5 Model Performance by Outbreak Phase**


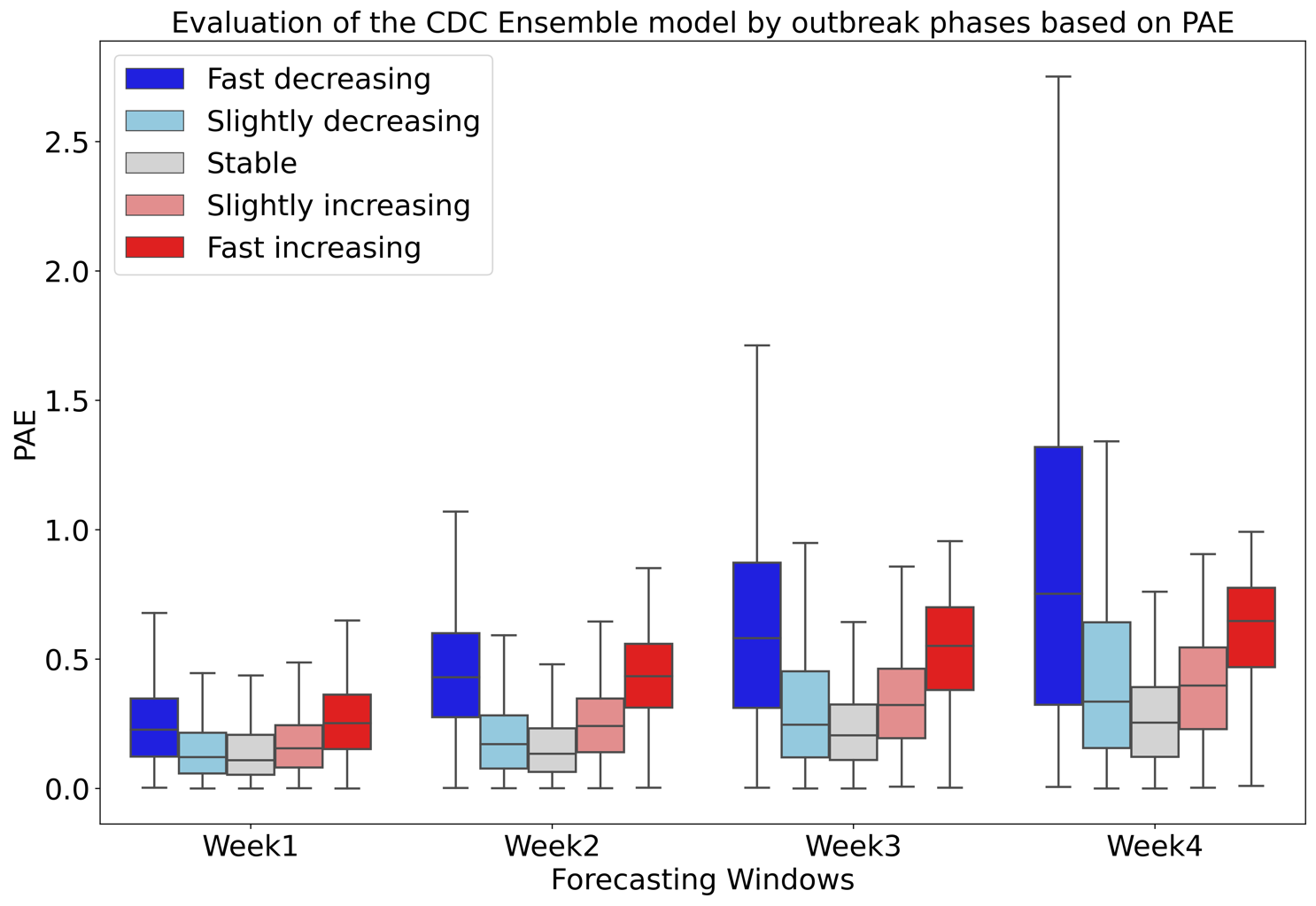


Supplementary Figure 24: Evaluation of the CDC Ensemble model by outbreak phases based on PAE. The colors represent different outbreak phases, and each bar represents the distribution of PAE in corresponding outbreak phases.


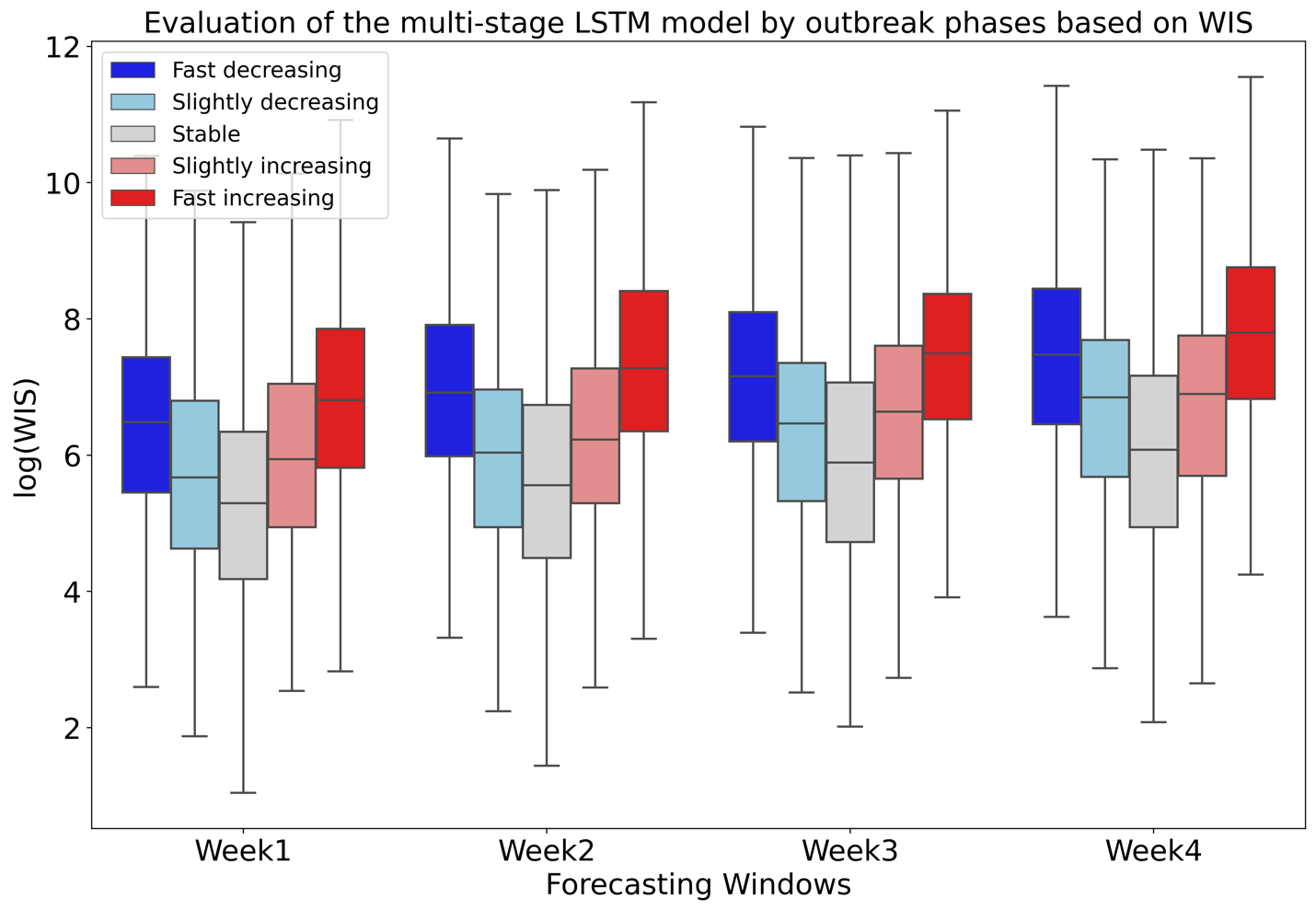


Supplementary Figure 25: Evaluation of the multi-stage LSTM model by outbreak phases based on WIS. The colors represent different outbreak phases, and each bar represents the distribution of WIS in corresponding outbreak phases.


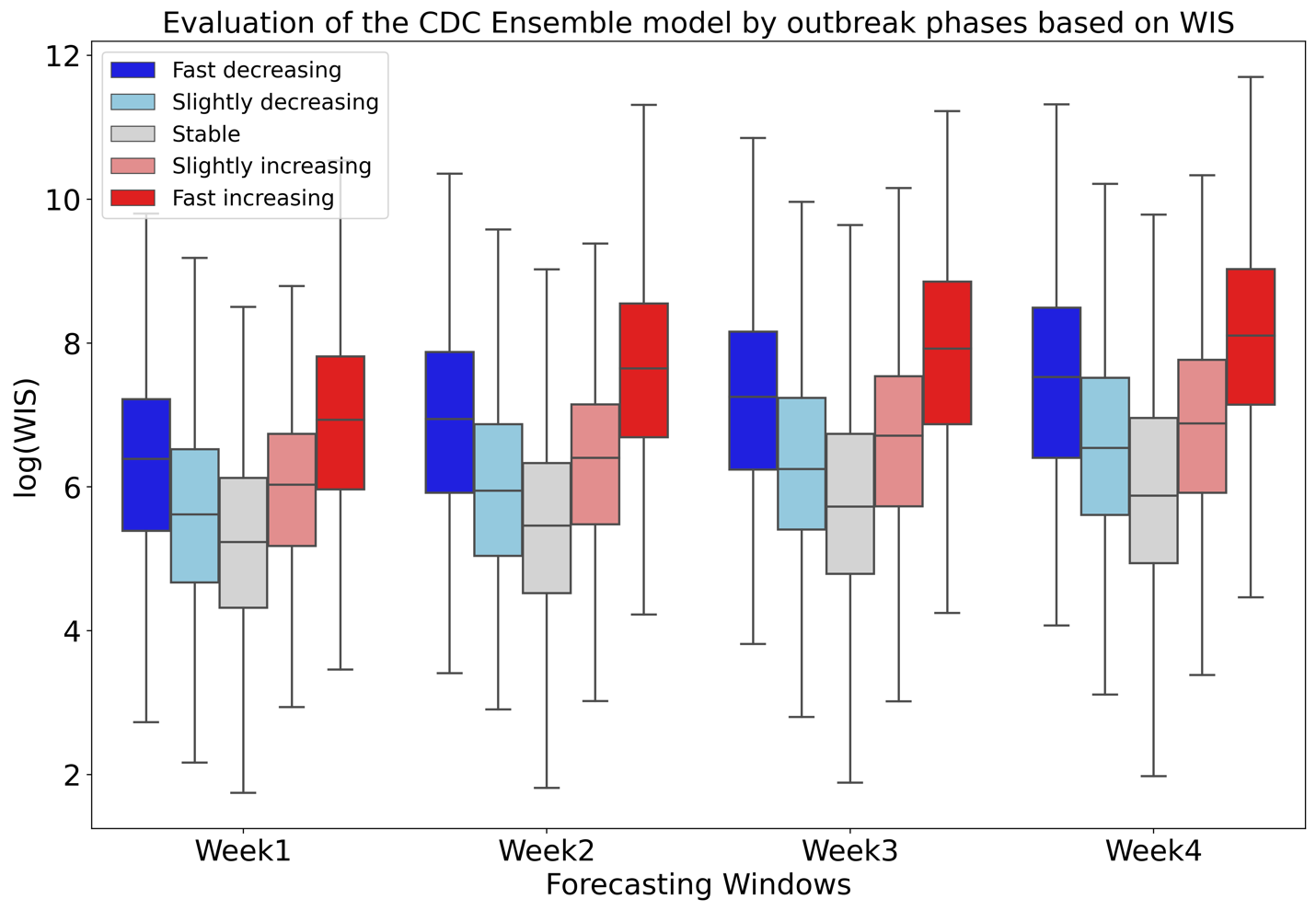


Supplementary Figure 26: Evaluation of the CDC Ensemble model by outbreak phases based on WIS. The colors represent different outbreak phases, and each bar represents the distribution of WIS in corresponding outbreak phases.

## **3.6 Compare model performance by outbreak with the CDC Ensemble model**

In addition to phase-based evaluation of our model directly, we also quantify our model performance relative to the CDC ensemble model for each outbreak phase. For each outbreak phase group $i$ and forecasting window $j$, we define a probability $P_{PAE}^{i,j}$ to indicate the frequency that multi-stage LSTM model outperforms the CDC ensemble model:

$$P_{PAE}^{i,j}= \frac{{\{PAE}_{LSTM}^{i,j}< {PAE}_{CDC}^{i,j}\}}{N_{i,j}}$$

where ${\{PAE}_{LSTM}^{i,j}< {PAE}_{CDC}^{i,j}\}$ represents the number of times that the multi-stage LSTM model has smaller PAE than the CDC ensemble within phase group $i$ and at forecasting window $j$, $N_{i,j}$ represents the total number of predictions assigned to one of five quantile outbreak phase group $i at forecasting window j$. Results of $P_{PAE}^{i,j}$ are illustrated in Appendix Figure 27, which illustrates the probability of LSTM performing better than the CDC Ensemble model based on PAE under different outbreak phases. The colors represent different outbreak phases and the y axis plots the $P_{PAE}^{i,j}$. The grey dash line indicates a probability of 0·5. The results based on AE are equivalent to PAE, hence we only present the analysis based on PAE and WIS.


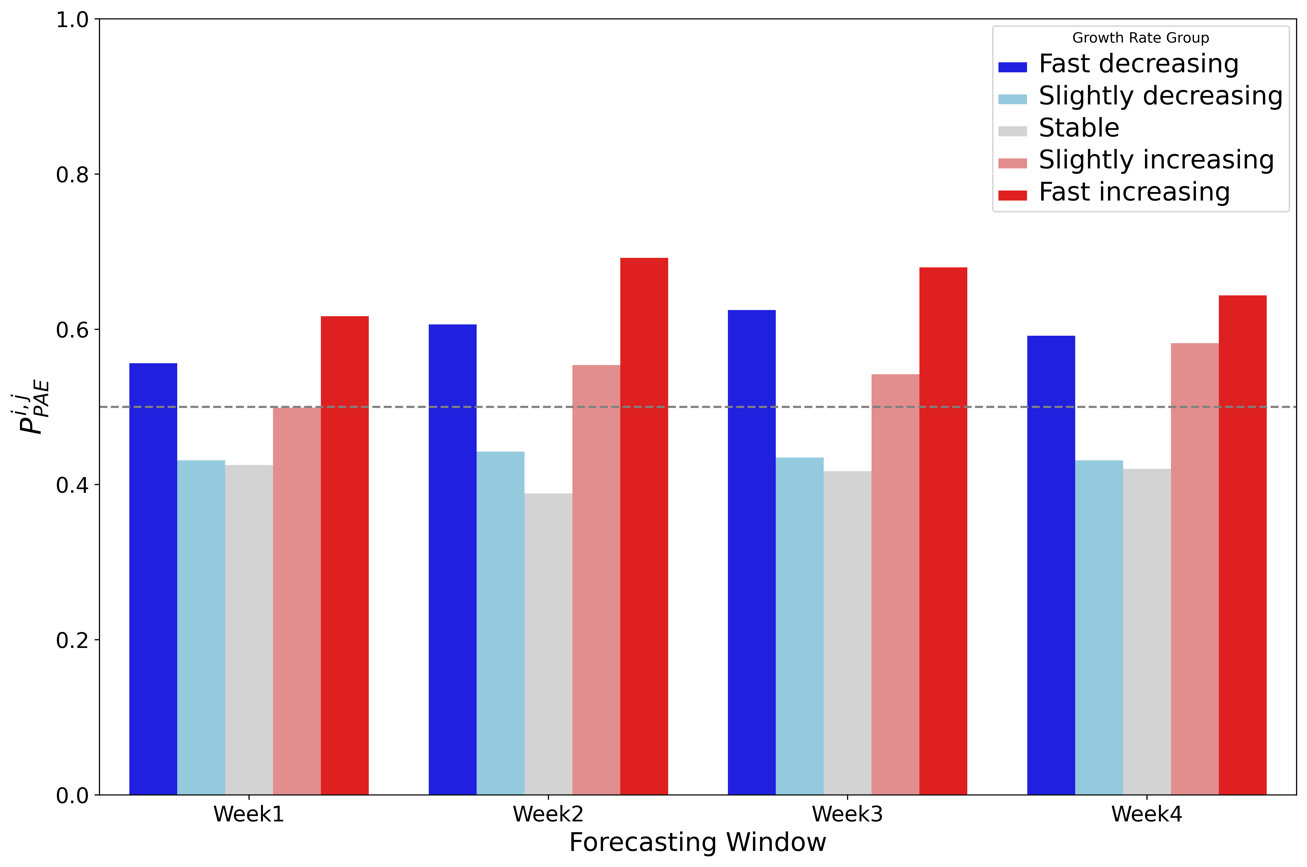


Supplementary Figure 27: The probability of LSTM performing better than the CDC Ensemble model based on PAE under different outbreak phase. The colors represent different outbreak phase and y axis plots the probability that the multi-stage LSTM outperforms the CDC ensemble under each phase group. The grey dash line indicates a probability of 0·5.

Similar to the definition of $P_{PAE}^{i,j}$, we define $P_{WIS}^{i,j}$ as the probability that multi-stage LSTM model outperforms the CDC ensemble model based on WIS:

$$P_{WIS}^{i,j}= \frac{{\{WIS}_{LSTM}^{i,j}< {WIS}_{CDC}^{i,j}\}}{N_{i,j}}$$

where ${\{WIS}_{LSTM}^{i,j}< {WIS}_{CDC}^{i,j}\}$ represents the number of times that the multi-stage LSTM model have smaller WIS than the CDC ensemble within phase group $i$ and at forecasting window $j$, $N_{i,j}$ represents the total number of predictions assigned to quantile outbreak phase group $i at forecasting window j$. Results of $P_{WIS}^{i,j}$ are illustrated in Appendix Figure 28, which illustrates the probability of LSTM performing better than the CDC Ensemble model based on WIS under different outbreak phases.

**
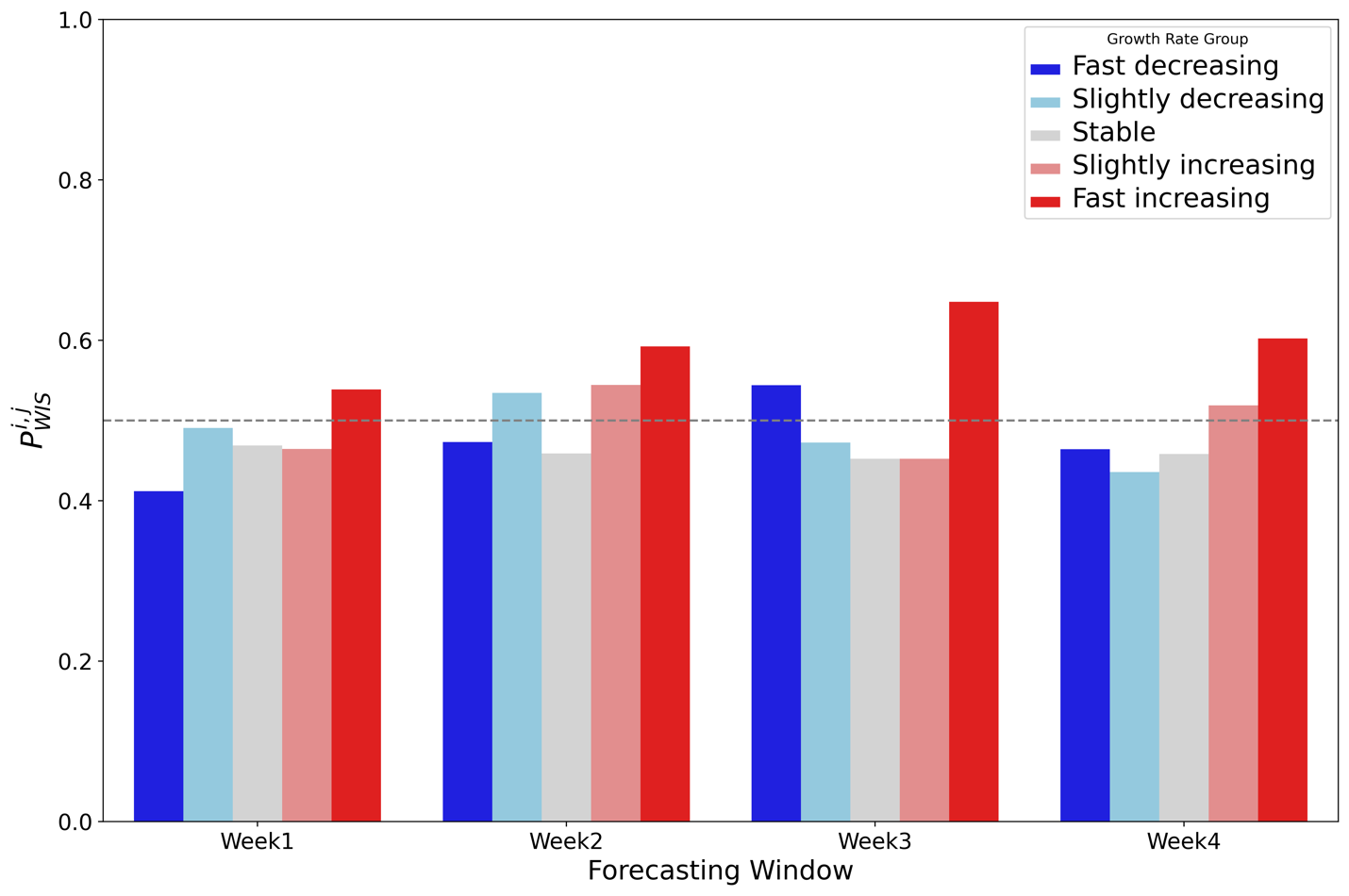
**

Supplementary Figure 28: The probability of LSTM performing better than the CDC Ensemble model based on WIS under different outbreak phase. The colors represent different outbreak phase and y axis plots the probability that the multi-stage LSTM outperforms the CDC ensemble under each phase group. The grey dash line indicates a probability of 0·5.

## **3.7 Comparing model performance after adding genomic cases data by AE and WIS**

Appendix Figure 29 and 30 illustrate the results for three different models based on AE and WIS: (a) Multi-stage LSTM model without variant cases data, (b) Multi-stage LSTM model with variant cases data and (c) CDC Ensemble model. The x-axis is the week that the predictions are made on. Each pair of bar plots represents PAE distribution for the selected states at a given week, where the green bar represents the error distribution for the multi-stage LSTM model without variant cases data, purple bar represents the error distribution for the multi-stage LSTM model with variant cases data, and the yellow bar represents the error distribution for the CDC ensemble model.

**
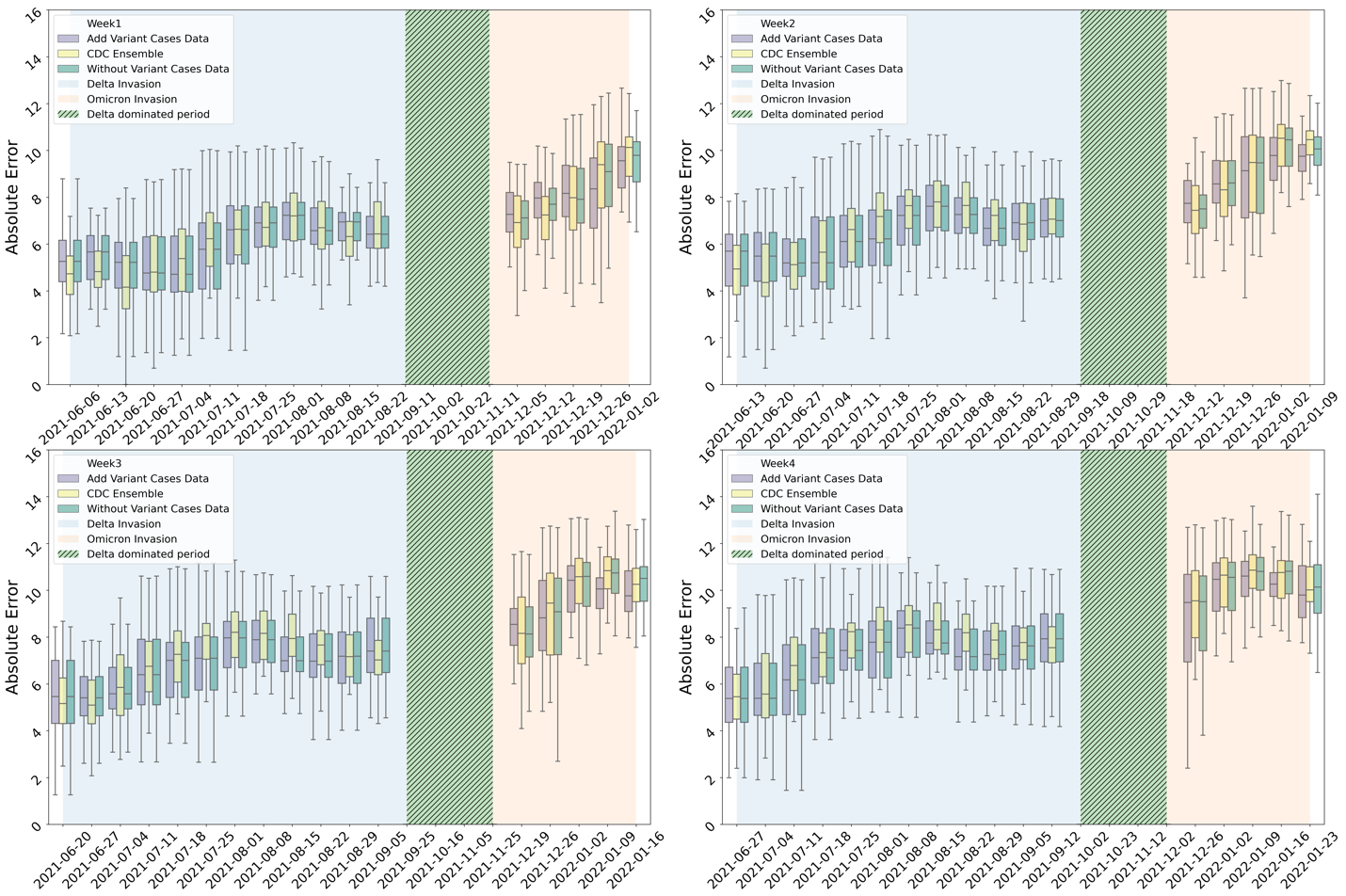
**

Supplementary Figure 29: Model performance based on absolute error in log scale for three different models: (a) Multi-stage LSTM model without variant cases data, (b) Multi-stage LSTM model with variant cases data and (c) CDC Ensemble model. The x-axis is the week that the predictions are made on. Each pair of bar plots represents PAE distribution for the selected states at a given week, where the green bar represents the error distribution for the multi-stage LSTM model without variant cases data, purple bar represents the error distribution for the multi-stage LSTM model with variant cases data, and the yellow bar represents the error distribution for the CDC ensemble model. The blue region represents the period of Delta invasion, the shaded green region represents Delta dominated period (proportion of Delta reaches 100%), and the orange region represents the period of Omicron invasion.


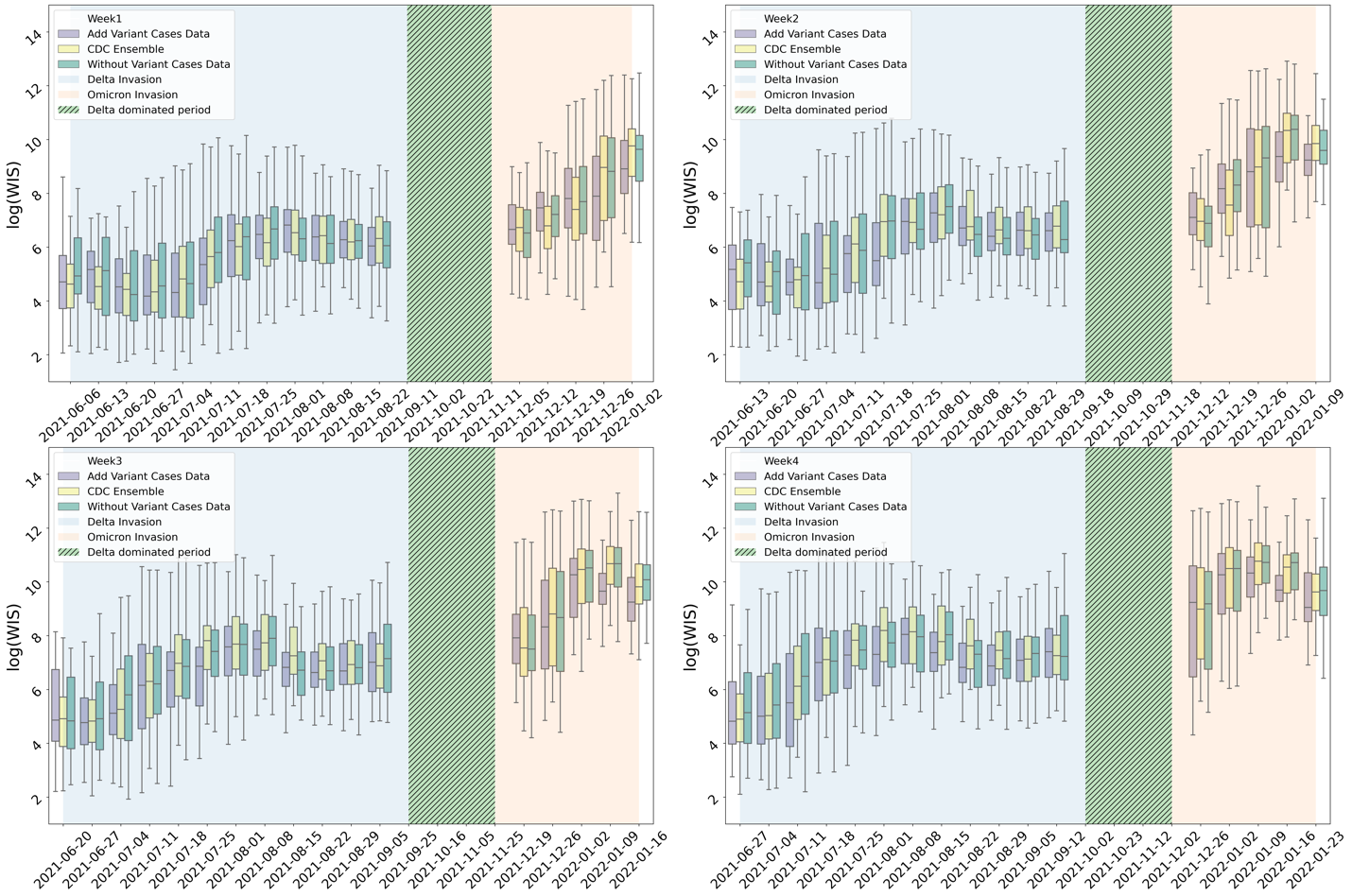


Supplementary Figure 30: Model performance based on log(WIS) for three different models: (a) Multi-stage LSTM model without variant cases data, (b) Multi-stage LSTM model with variant cases data and (c) CDC Ensemble model. The x-axis is the week that the predictions are made on. Each pair of bar plots represents PAE distribution for the selected states at a given week, where the green bar represents the error distribution for the multi-stage LSTM model without variant cases data, purple bar represents the error distribution for the multi-stage LSTM model with variant cases data, and the yellow bar represents the error distribution for the CDC ensemble model. The blue region represents the period of Delta invasion, the shaded green region represents Delta dominated period (proportion of Delta reaches 100%), and the orange region represents the period of Omicron invasion.

## **3.8 Results of weekly deaths forecasting**

We also apply the models for all epidemiological weeks from September 2020 to September 2021, each week we make weekly death predictions for the next 4 weeks. Appendix Figure 31-33 compares the model performance of the Multi-stage LSTM model with the CDC ensemble model at the state-level based on different error metric. Each pair of bar plots represents error distribution for all the states at given week, where the green bar represents the error distribution for the Multi-stage LSTM model and the yellow bar represents the error distribution for the CDC ensemble model. The red curve indicates the weekly reported deaths at the national level.

**
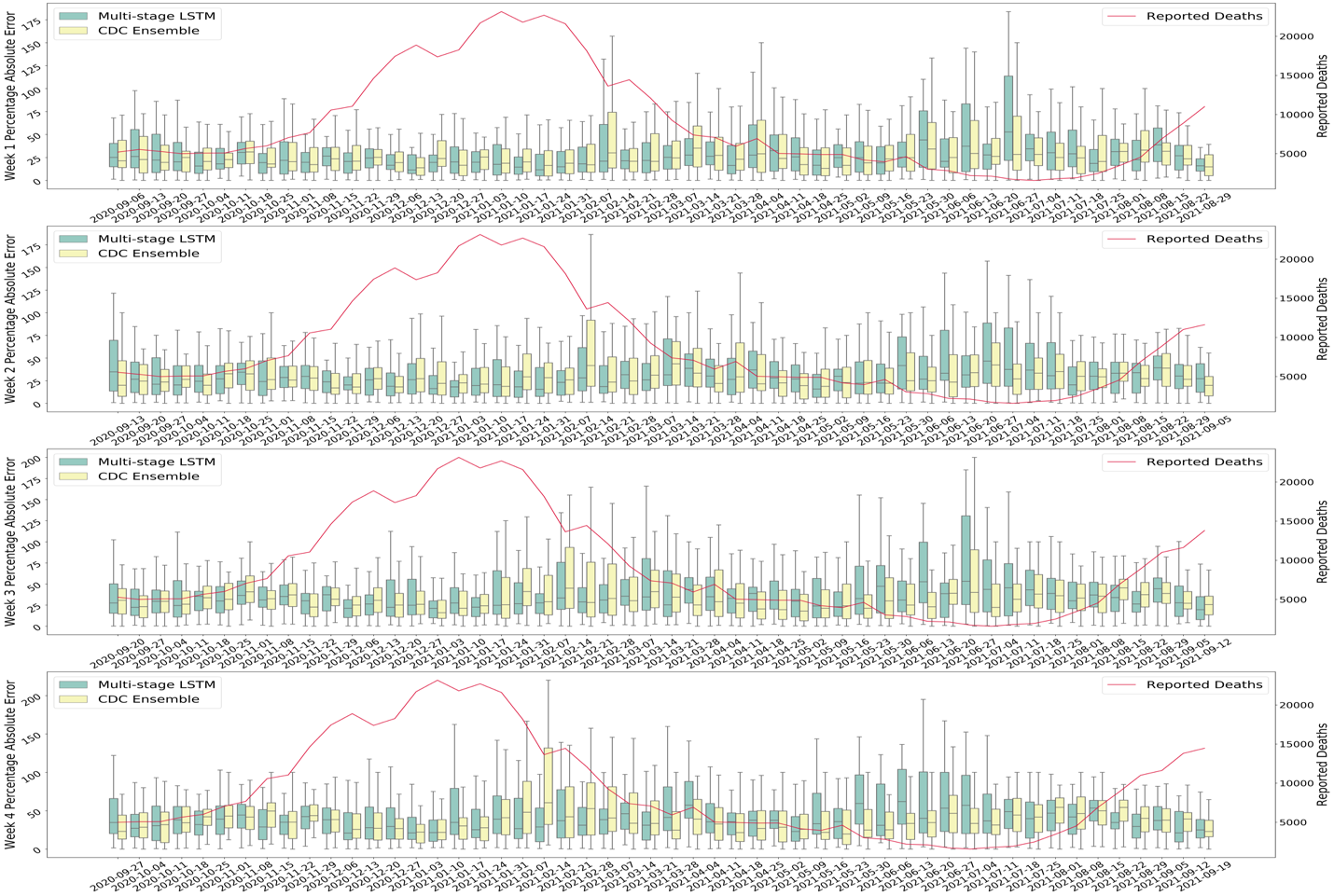
**

Supplementary Figure 31: Comparison of Deaths performance between the multi-stage LSTM Model and the CDC ensemble model based on PAE. The y-axis represents the PAE for 1-4 weeks deaths’ prediction results. Each pair of bar plots represents PAE distribution for all the states at a given week, where the green bar represents the error distribution for the multi-stage LSTM model, and the yellow bar represents the error distribution for the CDC ensemble model. The red curve represents the weekly reported deaths at the national level. The left y-axis represents the PAE by different forecasting windows and right y-axis represents national level reported deaths.


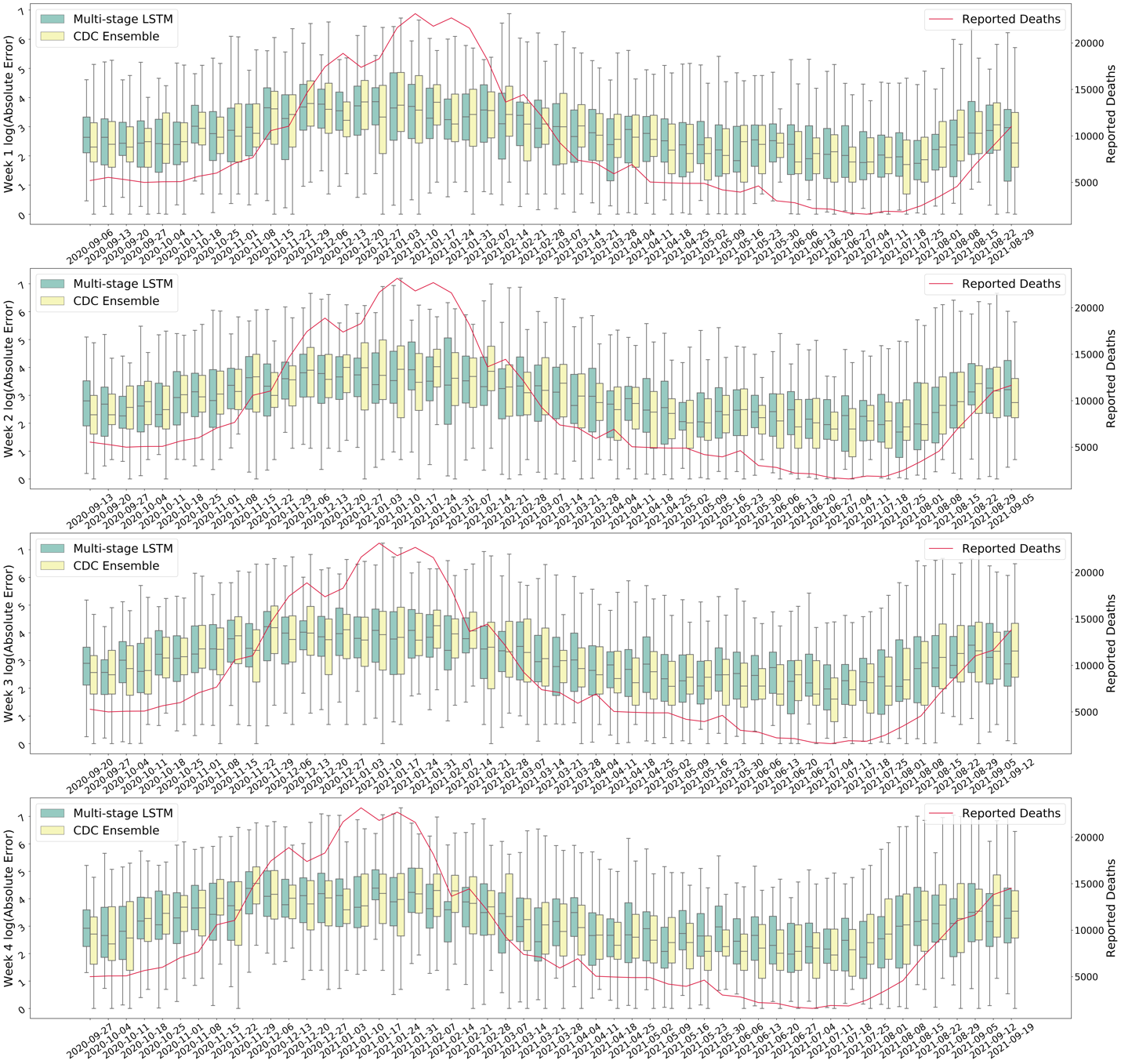


Supplementary Figure 32: Comparison of Deaths performance between the multi-stage LSTM Model and the CDC ensemble model based on log AE. The y-axis represents the AE for 1-4 weeks deaths’ prediction results. The y-axis represents the log(AE) for 1-4 weeks deaths’ prediction results. Each pair of bar plots represents log(AE) distribution for all the states at a given week, where the green bar represents the error distribution for the multi-stage LSTM model, and the yellow bar represents the error distribution for the CDC ensemble model. The red curve represents the weekly reported deaths at the national level. The left y-axis represents the log(AE) by different forecasting windows and right y-axis represents national level reported deaths.


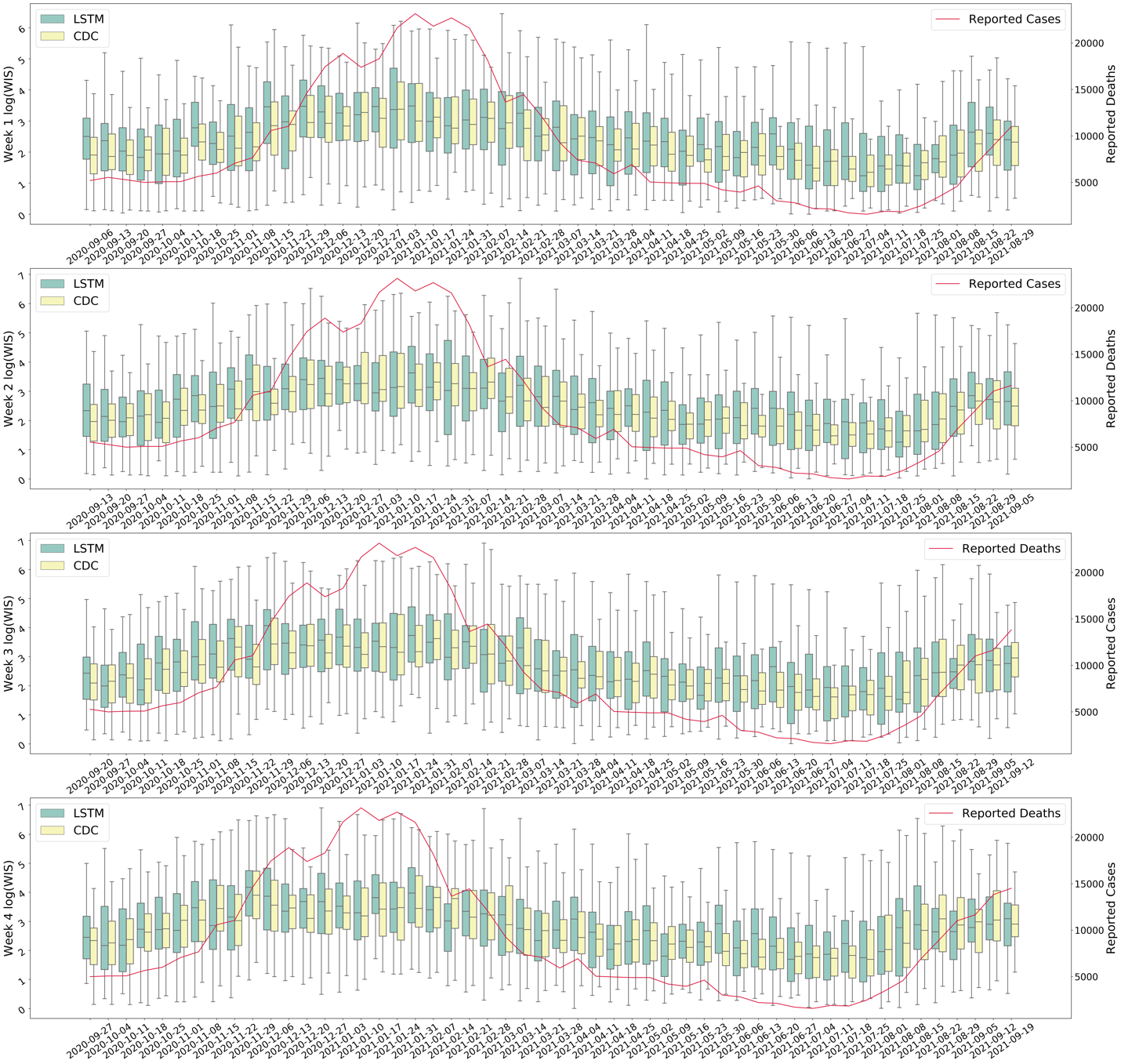


Supplementary Figure 33: Comparison of Deaths performance between the multi-stage LSTM Model and the CDC ensemble model based on log WIS. The y-axis represents the log WIS for 1-4 weeks deaths’ prediction results. The y-axis represents the log(WIS) for 1-4 weeks deaths’ prediction results. Each pair of bar plots represents log(WIS) distribution for all the states at a given week, where the green bar represents the error distribution for the multi-stage LSTM model, and the yellow bar represents the error distribution for the CDC ensemble model. The red curve represents the weekly reported deaths at the national level. The left y-axis represents the log(WIS) by different forecasting windows and right y-axis represents national level reported deaths.

For model selection for deaths prediction model, same as cases prediction model, we assign data into four categories and add hospitalization as part of the epidemiological data. The model performance based on different input data is shown in Appendix Figure 34.

**
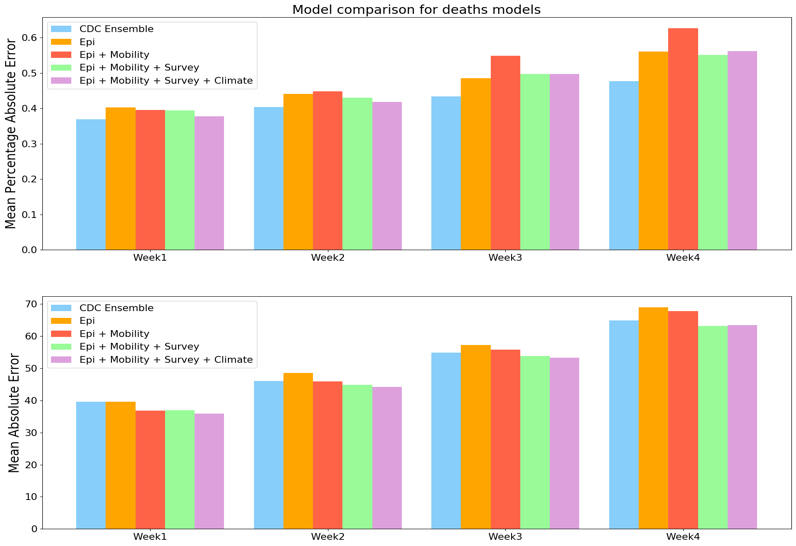
**

Supplementary Figure 34: Model comparison for deaths models by mean PAE and mean AE for the period between September 2020 to September 2021. The y-axis represents the average absolute error for 1-4 weeks deaths’ prediction results across selected period.

# **Reference**

1 Friedman J, Liu P, Troeger CE, *et al.* Predictive performance of international COVID-19 mortality forecasting models. *Nature communications* 2021; **12**: 1–13.

2 Vaid S, Cakan C, Bhandari M. Using machine learning to estimate unobserved COVID-19 infections in North America. *The Journal of bone and joint surgery American volume* 2020.

3 Anastassopoulou C, Russo L, Tsakris A, Siettos C. Data-based analysis, modelling and forecasting of the COVID-19 outbreak. *PloS one* 2020; **15**: e0230405.

4 Dong E, Du H, Gardner L. An interactive web-based dashboard to track COVID-19 in real time. *The Lancet Infectious Diseases* 2020; **20**: 533–4.

5 COVID-19 data. 2022; published online June 13. https://github.com/govex/COVID-19 (accessed June 14, 2022).

6 Dialysis COVID-19 Vaccination Data Dashboard | NHSN | CDC. 2022; published online Jan 31. https://www.cdc.gov/nhsn/covid19/dial-vaccination-dashboard.html (accessed June 14, 2022).

7 U.S. Department of Health & Human Services (HHS). HHS.gov. https://www.hhs.gov/index.html (accessed March 21, 2022).

8 David C. Farrow, Logan C. Brooks, Ryan J. Tibshirani, Roni Rosenfeld. Delphi Epidata API. GitHub. 2015. https://github.com/cmu-delphi/delphi-epidata (accessed March 21, 2022).

9 Badr HS, Du H, Marshall M, Dong E, Squire MM, Gardner LM. Association between mobility patterns and COVID-19 transmission in the USA: a mathematical modelling study. *The Lancet Infectious Diseases* 2020; **20**: 1247–54.

10 Ilin C, Annan-Phan S, Tai XH, Mehra S, Hsiang S, Blumenstock JE. Public mobility data enables COVID-19 forecasting and management at local and global scales. *Sci Rep* 2021; **11**: 13531.

11 Chang S, Pierson E, Koh PW, *et al.* Mobility network models of COVID-19 explain inequities and inform reopening. *Nature* 2021; **589**: 82–7.

12 Guan G, Dery Y, Yechezkel M, Ben-Gal I, Yamin D, Brandeau ML. Early detection of COVID-19 outbreaks using human mobility data. *PLOS ONE* 2021; **16**: e0253865.

13 Limitations of using mobile phone data to model COVID-19 transmission in the USA - The Lancet Infectious Diseases. https://www.thelancet.com/journals/laninf/article/PIIS1473-3099(20)30861-6/fulltext (accessed March 22, 2022).

14 Gatalo O, Tseng K, Hamilton A, Lin G, Klein E. Associations between phone mobility data and COVID-19 cases. *The Lancet Infectious Diseases* 2021; **21**: e111.

15 Places Data Curated for Accurate Geospatial Analytics | SafeGraph. https://www.safegraph.com (accessed Oct 14, 2021).

16 Weekly Patterns | SafeGraph Docs. SafeGraph. https://docs.safegraph.com/docs/weekly-patterns (accessed March 22, 2022).

17 Core Places | SafeGraph Docs. https://docs.safegraph.com/docs/core-places (accessed March 22, 2022).

18 Pearson K. LIII. On lines and planes of closest fit to systems of points in space. *The London, Edinburgh, and Dublin philosophical magazine and journal of science* 1901; **2**: 559–72.

19 McDonald D, Bien J, Green A, Hu AJ, Tibshirani R. Replication Data for: Can Auxiliary Indicators Improve COVID-19 Forecasting and Hotspot Prediction? 2021; published online Nov 8. DOI:10.5683/SP3/UW4VTC.

20 Reinhart A, Brooks L, Jahja M, *et al.* An open repository of real-time COVID-19 indicators. *PNAS* 2021; **118**. DOI:10.1073/pnas.2111452118.

21 Kerr GH, Badr HS, Gardner LM, Perez-Saez J, Zaitchik BF. Associations between meteorology and COVID-19 in early studies: Inconsistencies, uncertainties, and recommendations. *One Health* 2021; **12**: 100225.

22 Malki Z, Atlam E-S, Hassanien AE, Dagnew G, Elhosseini MA, Gad I. Association between weather data and COVID-19 pandemic predicting mortality rate: Machine learning approaches. *Chaos, Solitons & Fractals* 2020; **138**: 110137.

23 Sera F, Armstrong B, Abbott S, *et al.* A cross-sectional analysis of meteorological factors and SARS-CoV-2 transmission in 409 cities across 26 countries. *Nat Commun* 2021; **12**: 5968.

24 Badr HS, Zaitchik BF, Kerr GH, *et al.* Unified real-time environmental-epidemiological data for multiscale modeling of the COVID-19 pandemic. *medRxiv* 2021.

25 Dowd JB, Andriano L, Brazel DM, *et al.* Demographic science aids in understanding the spread and fatality rates of COVID-19. *Proc Natl Acad Sci U S A* 2020; **117**: 9696–8.

26 Bureau UC. State Population by Characteristics: 2010-2019. Census.gov. https://www.census.gov/data/tables/time-series/demo/popest/2010s-state-detail.html (accessed March 21, 2022).

27 Elbe S, Buckland‐Merrett G. Data, disease and diplomacy: GISAID’s innovative contribution to global health. *Global challenges* 2017; **1**: 33–46.

28 Hochreiter S, Schmidhuber J. Long short-term memory. *Neural computation* 1997; **9**: 1735–80.

29 Bracher J, Ray EL, Gneiting T, Reich NG. Evaluating epidemic forecasts in an interval format. *PLoS computational biology* 2021; **17**: e1008618.

30 Sundararajan M, Taly A, Yan Q. Axiomatic Attribution for Deep Networks. ; : 10.
